# Application of Feature-Based Molecular Networking for Comparative Metabolomics and Targeted Isolation of Stereoisomers from Algicolous Fungi

**DOI:** 10.3390/md20030210

**Published:** 2022-03-16

**Authors:** Bicheng Fan, Laura Grauso, Fengjie Li, Silvia Scarpato, Alfonso Mangoni, Deniz Tasdemir

**Affiliations:** 1 GEOMAR Centre for Marine Biotechnology (GEOMAR-Biotech), Research Unit Marine Natural Product Chemistry, GEOMAR Helmholtz Centre for Ocean Research Kiel, Am Kiel-Kanal 44, 24106 Kiel, Germany; bichengfan@hotmail.com (B.F.); fengjieli0620@hotmail.com (F.L.); 2 Dipartimento di Agraria, Università degli Studi di Napoli Federico II, 80055 Portici, Italy; laura.grauso@unina.it; 3 Dipartimento di Farmacia, Università degli Studi di Napoli Federico II, 80131 Napoli, Italy; silvia.scarpato@unina.it (S.S.); alfonso.mangoni@unina.it (A.M.); 4 Faculty of Mathematics and Natural Sciences, Kiel University, Christian-Albrechts-Platz 4, 24118 Kiel, Germany

**Keywords:** *Pyrenochaetopsis* sp., *Fucus vesiculosus*, feature-based molecular networking, pyrenosetin, decalinoylspirotetramic acid, stereoisomers, anticancer, malignant melanoma

## Abstract

Seaweed endophytic (algicolous) fungi are talented producers of bioactive natural products. We have previously isolated two strains of the endophytic fungus, *Pyrenochaetopsis* sp. FVE-001 and FVE-087, from the thalli of the brown alga *Fucus vesiculosus*. Initial chemical studies yielded four new decalinoylspirotetramic acid derivatives with antimelanoma activity, namely pyrenosetins A–C (**1**–**3**) from *Pyrenochaetopsis* sp. strain FVE-001, and pyrenosetin D (**4**) from strain FVE-087. In this study, we applied a comparative metabolomics study employing HRMS/MS based feature-based molecular networking (FB MN) on both *Pyrenochaetopsis* strains. A higher chemical capacity in production of decalin derivatives was observed in *Pyrenochaetopsis* sp. FVE-087. Notably, several decalins showed different retention times despite the same MS data and MS/MS fragmentation pattern with the previously isolated pyrenosetins, indicating they may be their stereoisomers. FB MN-based targeted isolation studies coupled with antimelanoma activity testing on the strain FVE-087 afforded two new stereoisomers, pyrenosetins E (**5**) and F (**6**). Extensive NMR spectroscopy including DFT computational studies, HR-ESIMS, and Mosher’s ester method were used in the structure elucidation of compounds **5** and **6**. The 3’*R*,5’*R* stereochemistry determined for compound **6** was identical to that previously reported for pyrenosetin C (**3**), whose stereochemistry was revised as 3’*S*,5’*R* in this study. Pyrenosetin E (**5**) inhibited the growth of human malignant melanoma cells (A-375) with an IC_50_ value of 40.9 μM, while **6** was inactive. This study points out significant variations in the chemical repertoire of two closely related fungal strains and the versatility of FB MN in identification and targeted isolation of stereoisomers. It also confirms that the little-known fungal genus *Pyrenochaetopsis* is a prolific source of complex decalinoylspirotetramic acid derivatives.

## 1. Introduction

Endophytic fungi inhabiting the seaweeds, also known as algicolous fungi, are a promising source of secondary metabolites with enormous chemical diversity and a wide range of biological activities [1,2,3]. More than 350 natural products belonging to various chemical families, e.g., polyketides, terpenoids, peptides and alkaloids, have been reported from macroalgae-derived fungi so far [4]. A prominent example is halimide, a diketopiperazine that derives from the seaweed-derived fungus *Aspergillus* sp. [5]. Plinabulin, the synthetic *tert*-butyl analogue of halimide, is currently undergoing phase III clinical trials for the treatment of non-small cell lung cancer [6], and testifies to the untapped potential of algicolous fungi for biomedical applications.

*Fucus vesiculosus* (Class Phaeophyceae, Order Fucales, Family Fucaceae) is one of the most widespread brown seaweeds in the shallow coasts of northern Europe, including the Baltic Sea [7]. Although the fungi are important members of the seaweed microbiome with evolutionary and ecological importance for the fitness of the seaweed holobiont [8,9], only a few studies describing the mycobiome of *Fucus* spp. are available in the literature [3,10,11,12,13,14,15]. Similarly, only a few *Fucus*-derived fungi have been subjected to chemical studies [3,11,16,17,18]. Hence, more and in-depth studies are necessary to identify the real potential of the endophytic fungi inhabiting the *Fucus* spp.

*Pyrenochaetopsis* is a relatively new fungal genus (introduced into family Cucurbitariaceae in 2010) closely related to genera *Phoma* and *Pyrenochaeta* [19]. The genus has started receiving research attention because of its many life styles, such as endophytic, saprophytic or plant pathogenic [19,20], and representation in almost all environments. *Pyrenochaetopsis* sp. have also been reported from the marine realm, e.g., from shrimp gut [21] and seaweeds [10,22]. Little is known about the secondary metabolite capacity of the genus *Pyrenochaetopsis*. The very first chemical study was published in 2017 on a soil-derived *Pyrenochaetopsis* sp. strain RK10-F058, and reported wakodecalines A and B, rare tricyclic decalin derivatives fused with a cyclopentanone ring and an *N*-methylated serine terminal group. Wakodecalines showed moderate antiplasmodial activity [23]; also isolated was the known decalin tetramic acid derivative, phomasetin, which moderately inhibited a few cancer cell lines and pathogenic bacteria or fungi [23].

In a previous study, we pinpointed the culture-dependent epiphytic and endophytic mycobiota of the Baltic brown alga *Fucus vesiculosus* and assessed their in vitro anticancer activity [10]. Of the 44 endophytic fungi isolated from the algal thallus, two were initially identified at order level (Pleosporales). Subsequent sequencing and phylogenetic tree analyses confirmed these fungi as two very close strains of *Pyrenochaetopsis* sp., namely FVE-001 and FVE-087 [16,17]. Both strains showed notable anticancer activity in initial bioassays [10]. For downstream studies, we applied a bioactive molecular networking approach on the SPE fractions of the chloroform subextract of *Pyrenochaetopsis* sp. strain FVE-001. This allowed us, effectively, to predict the novel and bioactive constituents of this strain with activity against a human melanoma cell line (A-375) at the initial fractionation stage. Targeted isolation studies on the most active fractions afforded three new tetracyclic decalinoylspirotetramic acid derivatives, pyrenosetins A–C (**1**–**3**) and the known decalin compound phomasetin, all exhibiting anti-melanoma activity [16]. A classical isolation approach adopted for the second *Pyrenochaetopsis* strain (FVE-087) yielded pyrenosetin D (**4**), plus the known decalins, wakodecalines A and B [17].

The discovery of pyrenosetin D with a very rare pentacyclic ring system [17] prompted us to reassess the metabolite profile of both *Pyrenochaetopsis* strains in a comparative manner by a feature-based molecular network (FB MN) through an untargeted metabolomics approach. The subsequent chemical work-up of this endophyte monitored by MN and antimelanoma activity yielded two new tetracyclic decalinoylspirotetramic acid stereoisomers, pyrenosetin E (**5**) and pyrenosetin F (**6**). Herein, we describe the FB-MN-assisted comparative metabolome analysis of *Pyrenochaetopsis* strains FVE-001 and FVE-087, followed by targeted purification, bioactivity and structure elucidation of two further tetracyclic decalin derivatives, pyrenosetin E (**5**) and pyrenosetin F (**6**), from *Pyrenochaetopsis* strain FVE-087.

## 2. Results

### 2.1. Strain Identification and Cultivation

The endophytic fungi FVE-001 (GenBank accession number: MH881440) and FVE-087 (GenBank accession number: MH881502) were isolated from the inner thallus of the brown alga *Fucus vesiculosus* collected at Kiel Fjord (Baltic Sea, Germany) as described [10,16,17]. Cultivation, extraction and Kupchan partition of both strains *Pyrenochaetopsis* sp. FVE-001 and FVE-087 have been outlined previously [16,17].

### 2.2. Comparative Metabolomics

The Kupchan chloroform (KC) subextracts of both fungi showed strong anticancer activity [16,17]. Initially, we probed the applicability of bioactivity-based MN to the SPE fractions obtained from the KC extract of *Pyrenochaetopsis* sp. strain FVE-001. However, in-depth assessments of the metabolome or chemical variations between the two *Pyrenochaetopsis* strains were not explored in our previous studies [16,17]. Here, we investigated the chemical profiles of the KC subextracts of both *Pyrenochaetopsis* strains using a UPLC-QToF-HRMS/MS based FB MN strategy. We focused on the KC subextracts, as the crude (EtOAc) fungal extracts had very low annotation rates and failed to identify any decalin derivatives [10], while the KC subextracts retained the bioactivity of the crude extracts that were highly enriched in decalin derivatives. The acquired MS^2^ data with the KC extracts was converted into .mzXML file format using the open-source data-converting tool MSconvert. The converted data were pre-processed with MZmine2 toolbox followed by an analysis of the Global Natural Products Social Molecular Network (GNPS) platform and visualized by Cytoscape [24].

Supplementary Figure S1 shows the overall chemical profile of the KC subextracts of both *Pyrenochaetopsis* strains in molecular networks. Of altogether 125 nodes that clustered into 10 chemical families, 102 corresponded to the FVE-087-KC subextract, while 90 nodes were mapped to that of FVE-001-KC (Figure S2). Notably, only 53.6% (67 of 125) of the nodes were shared by both subextracts, whereas 35 nodes were exclusive to the FVE-087-KC subextract, and 23 nodes to the FVE-001-KC subextract (Figure S2). This clearly showed the greater chemical diversity of *Pyrenochaetopsis* strain FVE-087.

**Figure 1:**
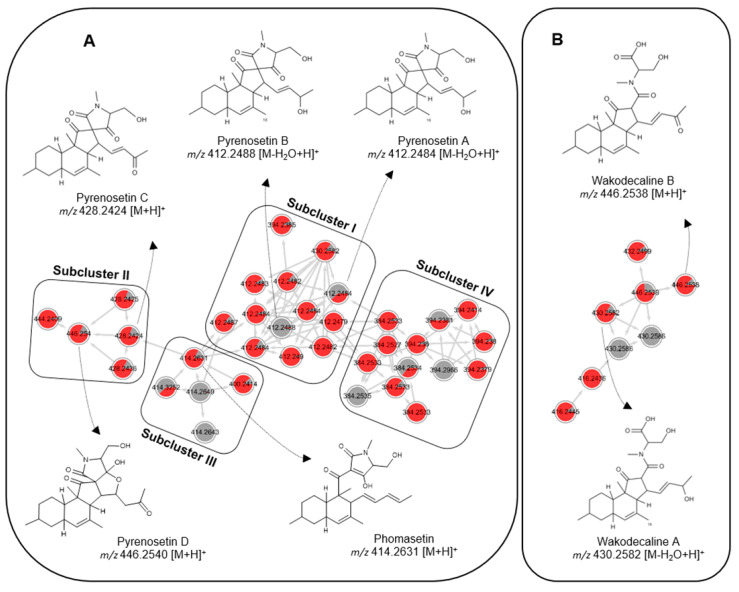
Molecular clusters observed in the KC subextracts of *Pyrenochaetopsis* sp. strain FVE-001 (gray) and strain FVE-087 (red). (**A**). Pyrenosetin/phomasetin cluster (**B**). Wakodecaline cluster.

Based on the comparison of the MS and MS/MS data of the pure compounds that we previously reported from this genus [16,17], two main clusters of the MN (**A** and **B**) appeared to be decalin derivatives (Figure 1, Table S1). The largest cluster, **A**, which contained 36 nodes in subclusters (**I**–**IV**), was annotated as the decalinoyltetramic acid family. Only three subclusters (**I**–**III**) were assigned to known compounds.

**Figure 2:**
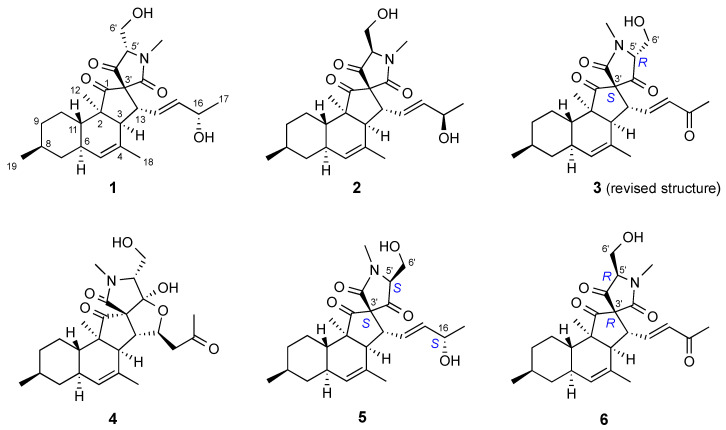
Structures of known pyrenosetins A–D (**1**–**4**) and new pyrenosetins E (**5**) and F (**6**).

Subcluster **I** (Figure 1) included 13 nodes, two of which were fully exclusive to *Pyrenochaetopsis* sp. strain FVE-087. Eleven nodes showed very similar [M-H_2_O+H]^+^ ions (from *m*/*z* 412.2479 to 412.2490) and similar HRMS/MS fragmentation patterns (Figure 1, Table S1); however, in the chromatogram, they represented individual peaks with different retention times (t_R_) ranging from 7.00 to 9.15 min. This suggested that they may be stereoisomers belonging to the same chemical family and could not be differentiated by conventional MS [25]. Based on the HRMS/MS fragmentation pattern and retention times, two nodes (*m*/*z* 412.2484 and 412.2488, [M-H_2_O+H]^+^) were identified as tetracyclic decalinoylspirotetramic acid derivatives pyrenosetin A (**1**, t_R_ 7.55 min) and B (**2**, t_R_ 7.78 min) (Figure 2), which we recently reported from *Pyrenochaetopsis* FVE-001 [16]. Other nodes could not be matched to any compound described before, hence subcluster **I** was identified to contain several putatively new stereoisomers of pyrenosetin A (**1**) and pyrenosetin B (**2**).

Subcluster **II** (Figure 1) comprised five nodes, three of which had *m*/*z* values of 428.2424, 428.2425, and 428.2436 ([M+H]^+^) and retention times at 7.88, 7.98, and 7.01 min, respectively, and were confidently annotated as pyrenosetin C (*m*/*z* 428.2424) and its stereoisomers based on their MS/MS fragmentation pattern (Table S1). A fourth node with *m*/*z* 446.2540 was annotated as the pentacyclic pyrenosetin D (**4**) [17]. The fifth node (*m*/*z* 444.2409), which was exclusive to *Pyrenochaetopsis* strain FVE-087, could not be assigned to any known metabolite.

Subcluster **III** (Figure 1) was also small and contained only five nodes, four of which exhibited similar *m*/*z* values (414.2631, 414.2643, 414.2649 and 414.3252) and almost identical retention times (approx. t_R_ 10.1 min). Notably, two nodes (*m*/*z* 414.2643 and 414.2649) were only found in *Pyrenochaetopsis* sp. strain FVE-001 KC extract. Their MS/MS fragmentation patterns suggested they belonged to phomasetin (*m*/*z* 414.2631, (M+H]^+^) type tricyclic decalinoyl tetramic acids [16]. Due to the very close retention times, it was difficult to deduce whether those nodes represented the same compound or overlapping peaks representing various stereoisomers.

We observed a fourth subcluster (**IV**) with 13 nodes with *m*/*z* values ranging between 384 and 294 (Figure 1). Subcluster **IV** was structurally very close to subcluster **I**. It was also the richest in terms of the number of nodes (eight) that were exclusively expressed in *Pyrenochaetopsis* sp. strain FVE-087. We were unable to annotate any of the nodes to any known compound by manual or GNPS-based dereplication strategies. This may indicate that subcluster **IV** contains potentially new metabolites with a different chemical subtype that is related to subcluster 1.

Cluster **B** consisted of eight nodes, three of which had *m*/*z* values 430.2582, 430.2586 and 430.2586 ([M-H_2_O+H]^+^) and were respectively annotated as wakodecaline A (a tricyclic decalinoyl tetramic acid with an *N*-methylated terminal serine moiety) and its two stereoisomers based on their MS/MS fragments. The other two nodes with *m*/*z* 446.2538 and 446.2539 ([M+H]^+^) shared the same planar structure with wakodecaline B based on their diagnostic MS/MS fragmentation patterns. The remaining four nodes in cluster **B** with *m*/*z* 432.2499, 416.2445, 416.2436 and 432.2499 could not be annotated, hence may represent potentially new wakodecaline derivatives.

Besides the clusters **A** and **B**, the global MN of both *Pyrenochaetopsis* strains (Figure S1) contained one large and several small clusters, some of which were fully specific for *Pyrenochaetopsis* sp. FVE-087, indicating the chemical wealth of this strain. However, we failed to find a match to any of the nodes in database analyses; hence, these clusters may belong to other, possibly new chemical families.

The results above clearly confirmed that strain FVE-087 had a higher chemical diversity, encouraging us to carry out an in-depth investigation on FVE-087-KC subextract. Due to their presence in larger amounts in bioactive KC fractions of *Pyrenochaetopsis* sp. FVE-087, we undertook a targeted isolation approach on putatively new compounds in subclusters **I** and **II**. Because of the low or no anticancer activity of phomasetin and wakodecalines in our previous studies [16,17], subcluster **III** and cluster **B** were not prioritized for in-depth chemical studies. The compounds in subcluster **IV**, or in the other unannotated clusters (Figure S1), had low quantities in the KC subextract that hampered their isolation.

### 2.3. Isolation and Structure Elucidation

The KC subextract of *Pyrenochaetopsis* sp. strain FVE-087 was fractionated by C18-SPE to yield 11 subfractions, and the antimelanoma activity was tracked to fractions 7–9. Most of the nodes detected in clusters **A** and **B** were identified in fraction F8. Reversed-phase HPLC purification of fraction F8 yielded the new compounds **5** and **6** (Figure 2).

Compound **5** was isolated as a colorless oil. HR-ESIMS analysis (Figure S11) of **5** returned a sodium adduct ion at *m*/*z* 452.2396 [M+Na]^+^, in agreement with the molecular formula C_25_H_35_NO_5_ requiring 9 double bond equivalents (DBEs). The FT-IR spectrum (Figure S12) indicated the presence of hydroxyl (*v*_max_ 3419 cm^−1^), carbonyl (*v*_max_ 1626 and 1731 cm^−1^) and aliphatic ether (*v*_max_ 1033 and 1053 cm^−1^) functions. The ^13^C NMR spectrum (Table 2, Figure S4) contained resonances belonging to four olefinic carbons (*δ*_C_ 127.8, 131.2, 132.1 and 136.8) and three carbonyl groups (*δ*_C_ 169.1, 204.8 and 209.6), accounting for five DBEs; hence, **5** had to be a tetracyclic compound. ^1^H NMR spectrum (CDCl_3_) (Table 1, Figure S3) revealed the presence of five methyl groups: two secondary (H_3_-17 *δ*_H_ 1.18, d, *J* = 6.3 Hz; H_3_-19 *δ*_H_ 0.89, d, *J =* 6.5 Hz,), one tertiary (H_3_-12 *δ*_H_ 0.98, s), one olefinic (H_3_-18 *δ*_H_ 1.70, s) and one *N*-methyl (H_3_-7′ *δ*_H_ 3.09, s). Three diastereotopic methylene protons, H_2_-7 (*δ*_H_ 0.85, m and 1.79, m), H_2_-9 (*δ*_H_ 0.97, m and 1.72, m), and H_2_-10 (*δ*_H_ 1.05, m and 1.43, m), and the oxymethylene protons (H_2_-6′ *δ*_H_ 3.87, m and *δ*_H_ 4.10, d, *J* = 11.7 Hz) were readily assigned by means of the DEPT-HSQC spectrum (Figure S5). Further detected were 10 complex methine protons (Table 1), including an (oxy)methine proton at *δ*_H_ 4.17 (H-16, m) and three olefinic methine protons belonging to H-14 (*δ*_H_ 5.82 dd, *J* = 15.4, 9.3 Hz), H-15 (*δ*_H_ 5.55, dd *J* = 15.4, 7.5 Hz), and H-5 (*δ*_H_ 5.22, br s) (Figure S3).

A careful inspection of the 1D and 2D NMR spectra (DEPT-HSQC, COSY and HMBC) suggested that **5** has an identical planar structure as pyrenosetin B (**2**) (Table 1 and Table 2, Figures S3–S7). The coupling constant pattern of axial protons H-7ax, H-9ax, H-10ax, and H-11ax demonstrated the *trans* junction of the A/B rings and the equatorial and β orientation of CH_3_-19. The NOESY correlations between H-6/H-8 and H-6/H_3_-12 determined the α orientation of CH_3_-12, while the strong NOESY correlation H-11/H-13 (Figure S8) established both the *cis* junction of the B/C rings and the α orientation of the side chain at C-13. Finally, the large coupling constant between H-14 and H-15 assigned the *E* geometry of the double bond at Δ^14(15)^. All of the configurations discussed so far were the same as in pyrenosetin B (**2**), so any difference between pyrenosetin E (**5**) and the latter compound must be located in the configurations of C-16, C-3’, or C-5’. Accordingly, the only slight differences between the ^1^H and ^13^C NMR data of the three compounds (Table 1 and Table 2) were observed in the spirotetramic acid region of the molecule.

**Figure 3:**
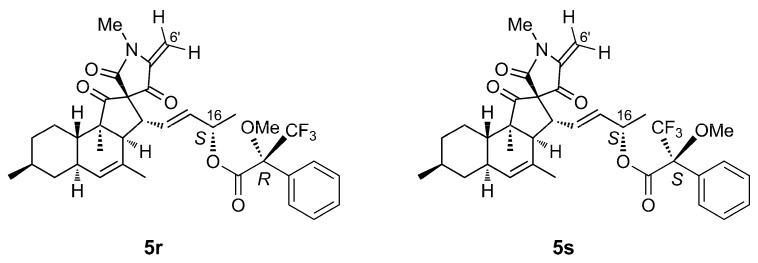
Structures of the Mosher esters **5r** and **5s** obtained from pyrenosetin E (**5**).

Configuration at C-16 was determined using Mosher’s method. The (*R*)- and (*S*)-MTPA esters of compound **5** were prepared using a reported procedure [26], and their ^1^H NMR spectra were acquired (Figures S9 and S10). The spectra showed that loss of water at C-6’ also occurred during the reaction, yielding the dehydrated esters **5r** and **5s** (Figure 3). Specifically, signals for H-5’ and for the two diastereotopic protons at C-6’ were absent in the ^1^H NMR spectra of **5r** and **5s**, being replaced by a pair of geminal vinyl protons (**5r**: *δ*_H_ 5.21 (d, *J =* 2.3 Hz) and 4.54 (d, *J =* 2.3 Hz); **5s**: *δ*_H_ 5.19 (d, *J =* 2.3 Hz) and 4.52 (d, *J =* 2.3 Hz)).

**Figure 4:**
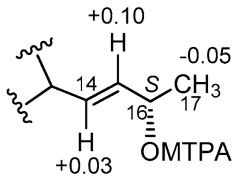
Δ*δ^SR^* (i.e., *δ_S_*–*δ_R_*) values (ppm) measured for *S-* and *R*-MTPA esters of pyrenosetin E (**5**).

This side reaction did not affect the interpretation of the experiment; in fact, it made it easier because a possible influence of a second MTPA group at position 6’ was avoided. The Δ*δ^SR^* values determined for the side-chain signals clearly indicated [26] the *S* absolute configuration of the secondary OH group at C-16 (Figure 4). The NOESY spectrum of **5** failed to reveal correlations diagnostic for the configuration at C-3’ and C-5’; even ^1^H-^13^C coupling constant analysis, which was used successfully for pyrenosetin D [17], was not helpful in this case. Therefore, we resorted to DFT prediction of chemical shifts.

The four stereoisomers of pyrenosetin E (**5**) with different configurations at C-3’ and C-5’ were studied. A set of conformers was generated for each stereoisomer by varying systematically the dihedral angles around the C-13/C-14, C-15/C-16, C-16/O-16, C-5’/C-6’, and C-6’/O-6’ bonds. The conformers were optimized by DFT at the B3LYP/6-31G(d,p) level using the SMD solvent model. Single-point calculations at higher level (TZVP basis set) provided a more accurate evaluation of the electronic energy of each conformer. Vibrational frequency calculations confirmed that all conformers were true energy minima and provided the Gibbs free energy of each conformer, which was used to calculate conformer populations through Boltzmann statistics. Calculation of the NMR isotropic shieldings at the mPW1PW91/6-311+G(d,p)/PCM level was performed for conformers populated by more than 1%. Boltzmann-averaged chemical shifts were used as input for the DP4+ analysis [27], which showed a 99.99% probability for the (3’*S*,5’*S*) configuration of pyrenosetin E (**5**) (Figure S21). Therefore, based on biogenetic grounds that the absolute configuration of the decalin part of the molecule is the same as in the other reported pyrenosetins, the full stereochemistry of pyrenosetin E (**5**) was determined as (2*R*,3*S*,6*R*,8*S*,11*S*,13*R*,16*S*,3’*S*,5’*S*).

Compound **6** was also obtained as a colorless oil. The molecular formula C_25_H_33_NO_5_ requiring 10 DBEs was assigned on the basis of a molecular ion *m*/*z* 428.2422 [M+H]^+^ observed in its HR-ESIMS spectrum (Figure S19). The ^1^H NMR spectrum of **6** (Figure S13) was similar to those of pyrenosetins B (**2**) and E (**5**), except for the disappearance of the oxymethine H-16 signal and significant downfield shift of the H-14 and H_3_-17 resonances (Table 1). The observation of an additional carbonyl signal at *δ*_C_ 198.8 (C-16, Table 2) and the HMBC correlations from H-16 to C-14, C-15 and C-17 (Figure S17) suggested that **6** contained a ketone group at C-16. Hence, we propose a planar structure for **6** that is identical to that of pyrenosetin C (**3**) (Figure 2). The coupling constant patterns and NOESY correlations (Figure S18) observed for **6** were very similar to those discussed for pyrenosetin E (**5**), indicating that stereochemistry of pyrenosetin F (**6**) at the chiral centers C-2, C-3, C-6, C-8, C-11, and C-13 was homologous to that of pyrenosetins E (**5**) and C (**3**). NOESY spectrum, however, lacked clear correlations for unambiguous identification of the relative configuration of C-3’ and C-5’ in the spirotetramic acid unit.

DFT prediction of chemical shifts of the four possible stereoisomers that differ in C-3’ and C-5’ configurations was, hence, performed using the protocol described above. Analysis of the results using DP4+ suggested a 92.6% probability for the (3’*R*,5’*R*) configuration of pyrenosetin F (**6**) (Figure S22). However, the (3’*R*,5’*R*) configuration matched that previously reported for pyrenosetin C (**3**) [16], meaning the reported stereochemistry of pyrenosetin C needed revision. Indeed, when the predicted NMR chemical shifts were analyzed against the experimental chemical shifts of pyrenosetin C (**3**) reported in ref. [16], the DP4+ probability for the (3’*R*,5’*R*) configuration of pyrenosetin C (**3**) was calculated as 0.00%, while the probability for the (3’*S*,5’*R*) configuration was calculated as 99.94% (Figure S23). A retrospective examination of structure elucidation of pyrenosetin C (**3**) showed that its spectroscopic data were compatible with the (3’*S*,5’*R*) configuration. Therefore, the full stereochemistry of pyrenosetin F (**6**) was determined as (2*R*,3*S*,6*R*,8*S*,11*S*,13*R*,3’*R*,5’*R*), while the stereochemistry of pyrenosetin C (**3**) was revised to (2*R*,3*S*,6*R*,8*S*,11*S*,13*R*,3’*S*,5’*R*), as shown in structure **3** (Figure 2).

Our previous research highlighted the anticancer activities of pyrenosetins against a malignant melanoma cell line (A-375) [16,17]. Compound **5** showed moderate activity against A-375 cells with an IC_50_ value of 40.9 μM. Compound **6** was inactive against A-375 even at the highest test concentrations (IC_50_ 200 μM), which may indicate the importance of the presence of an OH group at C-16.

## 3. Discussion

The genus *Pyrenochaetopsis* is widely distributed in various ecological niches, including soil, plant, airborne, and even human tissues [19,20,28]. It is also known from marine environments, such as the green alga *Flabellia petiolata* and the brown alga *Fucus vesiculosus* [16,22]. Being a newly introduced genus, the chemical machinery of the genus *Pyrenochaetopsis* has so far remained untapped. Indeed, the very first study on secondary metabolites of this genus was reported in 2017 by Nogawa et al. [23], who obtained the new decalins wakodecalines A and B, and the known phomasetin from a soil-derived *Pyrenochaetopsis* sp. (strain RK10-F058). Subsequent studies by our group on seaweed-derived *Pyrenochaetopsis* sp. yielded new pyrenosetins A–D (**1**–**4**), as well as phomasetin and wakodecalines A and B, totaling seven decalin derivatives from this genus [16,17]. Due to intriguing chemical constituents and the antimelanoma activity of the algicolous *Pyrenochaetopsis* sp., we decided to compare the metabolome profiles of the KC subextracts of both *Pyrenochaetopsis* strains FVE-001 and FVE-087, which are highly enriched in decalinoyltetramic acid derivatives. For this aim, we employed FB-MN, which showed that decalin types of compounds were highly abundant in both strains, and that strain FVE-087 is much richer in these compounds. Furthermore, as shown in Figure 1, FB-MN successfully grouped subtypes of decalin derivatives according to (i) the substitution level on their side chain (OH or keto group at C-16, pyrenosetins A–D), (ii) lack of spiropentanone group and substitution on the side chain (phomasetins), and (iii) the nature of the appendix (terminal *N*-methylated tetramic acid, or *N*-methylated serine moiety: wakodecalines). The recently developed MS^2^ based GNPS tool, FB MN, which can access several MS^1^ features such as retention times, indicated the presence of decalins as individual peaks in the chromatogram with almost identical MS data and fragmentation patterns, but with different retention times. This was very helpful for assessing the greater chemical space of the *Pyrenochaetopsis* strain FVE-087 and prioritization of isolation of some new stereoisomers, such as **5** and **6**.

FB MN analysis indicated the presence of many additional nodes (compounds) in the decalinoylspirotetramic acid clusters in both strains (including the entire subcluster **IV**) that did not match with any known compound in multiple databases. This suggests the presence of many potentially new decalinoylspirotetramic acid derivatives, especially in the KC subextract of *Pyrenochaetopsis* strain FVE-087, which we failed to isolate in sufficient amounts. MN-based untargeted metabolomics study also demonstrated the presence of additional molecular clusters with varying numbers of nodes (Figure S1) that could not be linked to any molecular family or individual compound, suggesting that the chemical diversity of *Pyrenochaetopsis* sp. is not limited to decalin type compounds. Indeed, the Oberlies group recently reported cytotoxic naphthoquinones (monomeric or dimeric) from another *Pyrenochaetopsis* sp. (strain MSX63693, origin not mentioned) [29], suggesting the wider chemical diversity of this underexplored filamentous fungus for future studies.

Decalin derivatives isolated from the genus *Pyrenochaetopsis* sp. possess multiple ring systems, often with a terminal tetramic acid ring. Wakodecalines are tricyclic compounds comprising a decalin and a spiropentanone ring, plus a terminal *N*-methylated serine moiety. Phomasetin lacks the spiro ring but possesses a terminal tetramic acid, hence also tricyclic. Pyrenosetins A–C obtained from *Pyrenochaetopsis* strain FVE-001 encompass a tetracyclic backbone bearing a cyclopentanone-fused decalin skeleton and an *N*-methylated spirotetramic acid ring [16]. Pyrenosetin D isolated from *Pyrenochaetopsis* strain FVE-087 is pentacyclic and contains an additional hemiacetal system [17]. Such a chemical scaffold is very rarely found in nature, and only a few fungal metabolites, e.g., fusarisetins A–D isolated from *Fusarium* sp. [30,31], altercrasins A–D isolated from *Alternaria* sp. [32,33], and diaporthichalasin isolated from *Diaporthe* sp. have similar skeletons [34]. As reported by Kato et al. [35], the decalin derivatives obtained from *Pyrenochaetopsis* sp. are mixed biosynthetic products of PKS and NRPS pathways (despite the established generic name of decalin that is typical for terpenes), most probably biosynthesized by an enzymatic intramolecular Diels–Alder cycloaddition. However, the biogenesis of these compounds has not been well-investigated. Until now, only one study reported the biosynthetic gene cluster analysis of the soil-derived *Pyrenochaetopsis* sp. RK10-F058 [35]. The previous research revealed the importance of the phm gene cluster in the biosynthesis of phomasetin [35]. However, the stereospecific biosynthesis of the tetramic acid ring and spiro center in pyrenosetins has received limited research interest so far. The purification of new stereoisomers in this report suggests that the tetramic acid ring and the spiro center could have different configurations during biosynthesis. Further exploration of pyrenosetins would be intriguing to understand their biosynthesis, particularly in terms of their true chemical diversity and bioactivity.

Determination of the stereochemical configuration of the decalinoylspirotetramic acid derivatives has been challenging, as NOESY spectroscopy often does not provide clear information. It was immediately clear that pyrenosetins E and F are stereoisomers of pyrenosetins A/B and C, respectively, by MS and NMR data or retention times, as suggested by FB MN analyses. However, the configuration at C-3’ and C-5’could not be determined directly from NMR data, because no clearly diagnostic NOE were present in their NOESY spectra. X-ray crystallography has been regarded as the best method for the unambiguous identification of their (absolute) configurations [30], but we were unable to grow high quality crystals of our compounds, which were isolated in very small amounts. Even ^1^H-^13^C coupling constants, which were used successfully for elucidation of the stereochemistry of pyrenosetin D [17], were not useful in this case. However, DFT prediction of NMR chemical shifts successfully solved this challenging stereochemical problem, plus, it provided convincing evidence to revise the stereochemistry of pyrenosetin C (**3**), showing once more the value of computational methods in elucidating the structure of complex natural products.

## 4. Materials and Methods

### 4.1. General Procedures

The specific rotation values of the compounds were measured on a Jasco P-2000 polarimeter (Jasco, Pfungstadt, Germany) at 20 °C. FT-IR spectra were recorded on a PerkinElmer Spectrum Two FT-IR spectrometer (PerkinElmer, Boston, MA, USA). The HR-mass spectra of the pure compounds were recorded on a microTOF II-High-performance TOF-MS system (Bruker^®^, Billerica, MA, USA) equipped with an electrospray ionization (ESI) source. The NMR spectra were acquired on a Bruker AV 600 spectrometer (Bruker^®^, Billerica, MA, USA). The residual solvent signals were used as internal reference: *δ*_H_ 7.26/*δ*_C_ 77.2 ppm (CDCl_3_). 4-Dimethyl-4-silapentane-1-sulfonic acid (DSS) was used as an internal standard. Tandem mass (MS/MS) spectrometry data were recorded on a Waters Xevo G2-XS QToF Mass Spectrometer (Waters^®^, Milford, MA, USA) connected to a Waters Acquity I-Class UPLC system (Waters^®^, Milford, MA, USA) in positive mode. Solid-phase extraction (SPE) was performed on a C18 cartridge (50 μm, 65 Å, Phenomenex, 411 Madrid Avenue, Torrance, CA, USA). The semi-preparative HPLC separations were performed on a VWR Hitachi Chromaster system (VWR International, Allison Park, PA, USA) consisting of a 5430 diode array detector (VWR International, Allison Park, PA, USA), a 5310 column oven, a 5260 autosampler, and a 5110 pump. The eluents used for HPLC separations were milli Q water (A) and MeCN (B). Routine HPLC separations were performed on a semi-preparative C18 monolithic column (Onyx, 100 × 10 mm, Phenomenex, Torrance, CA, USA) and an analytical synergi polar-RP 80 Å LC column (250 × 4.6 mm, Phenomenex, Torrance, CA, USA). The organic solvent used for MS/MS analysis was ULC/MS grade. Solvents used for purification were HPLC grade. An in-house Arium^®^ Water Purification System (Sartorius, Goettingen, Germany) was used for the preparation of milli Q water. Potato extract and dextrose used for fungal cultivation were purchased from Sigma-Aldrich (Schnelldorf, Germany) and Merck (Darmstadt, Germany), respectively. Agar was purchased from Applichem (Darmstadt, Germany).

### 4.2. Strain Identification, Cultivation and Kupchan Partition and Initial Bioassays

Strains *Pyrenochaetopsis* sp. FVE-001 (GenBank accession number: MH881440) and FVE-087 (GenBank accession number: MH881502) were obtained from *Fucus vesiculosus* sampled in Falckenstein Beach (54°23′22.6″ N, 10°11′26.4″ E, Kiel Fjord, Baltic Sea, Germany) [10]. The strain identification and cultivation have been described previously [16,17]. Briefly, both *Pyrenochaetopsis* strains were pre-cultured on potato dextrose agar (PDA: potato extract 4 g, dextrose 20 g, agar 15g for 1 L, pH 5.6). After pre-culturing, the conidia were inoculated in 500 mL cylindrical flasks containing 100 mL of medium (PDM: potato extract 4 g, dextrose 20 g for 1 L; pH 5.6) and incubated at 22 °C for 7 days on a rotary shaker at 120 rpm to prepare the seed. After 7 days of cultivation, 1 mL liquid seed was added into 2 L flasks that contained 800 mL PDM liquid medium. The large-scale fermentation was performed at 22 °C in the dark for 14 days on a rotary shaker (120 rpm). The large-scale cultivation broth was partitioned against the same volume of EtOAc twice at room temperature. After that, crude extract was dissolved in 90% MeOH and subjected to a modified Kupchan partition to yield three subextracts, *n*-hexane (KH), CHCl_3_ (KC), and aqueous MeOH (KM) [16,17]. All subextracts derived from both *Pyrenochaetopsis* sp. (FVE-001 and FVE-087) were tested for their bioactivity against five cancer cell lines and non-cancerous cell line HaCaT [16,17]. As described before, the KC subextracts from the two *Pyrenochaetopsis* sp. FVE-001 and FVE-087 showed the highest anticancer activities [16,17].

### 4.3. UPLC-QToF-MS/MS-Based Metabolome Analyses

The KC subextracts were analyzed on an ACQUITY UPLC I-Class System coupled to a Xevo G2-XS QToF Mass Spectrometer equipped with an electrospray ionization (ESI) source operating with a mass range of *m*/*z* 50–1600 Da. Each fraction was diluted and filtered through a 0.2 µm PTFE syringe filter to give a system equipped with an Acquity UPLC HSS T3 column (High Strength Silica C18, final concentration of 0.1 mg/mL) operating at 40 °C. Filtered samples were injected (injection volume: 0.5 µL) into the QToF (8 µm, 100 × 2.1 mm I.D., Waters^®^).

The chemical analysis was processed with a binary LC solvent system controlled by MassLynx^®^ (version 4.1) using mobile phase A (99.9%) water/0.1% formic acid (ULC/MS grade) and B 99.9% ACN/0.1% formic acid (ULC/MS grade), pumped at a rate of 0.6 mL/min with the following gradient: initial, 1% B; 0.0–11.5 min to 100% B; 11.5–12.5 min 100% B, and a column reconditioning phase to 1% B for 15 min. ESI conditions were set as follows: capillary voltage at 0.8 kV, sample cone voltage at 40.0 V, source temperature at 150 °C, desolvation temperature at 550 °C, cone gas flow in 50 L/h and desolvation gas flow in 1200 L/h. MS/MS setting was a ramp collision energy (CE): low CE from 6 eV to 60 eV and high CE from 9 eV to 80 eV. As a control, solvent (methanol) was injected. MassLynx^®^ (Waters^®^, V4.1) was used to analyze the achieved MS and MS^2^ data. Each run was repeated three times.

The raw data were converted to .mzXML file format using MSconvert (version 3.0.10051, Vanderbilt University, Nashville, TN, USA). The resulting .mzXML data were processed in MZmine2 (version 2.32) [36]. Mass lists were created using the mass detection module with a noise level of 100 and 10 counts for MS^1^ and MS^2^, respectively. The chromatogram was built using peak lists with a scan retention time from 0.00 min to 13.0 min, a minimum peak height of 100 counts, and an *m*/*z* tolerance of 0.05 Da or 30 ppm. Deconvolution process was applied to peak list by using the local minimum search algorithm with the following parameters: chromatographic threshold 0.01%, minimum relative height 0.01%, minimum absolute height 100 counts, minimum ratio of peak top/edge 2, peak duration range 0.01–3 min. For MS^2^ scan pairing, the *m*/*z* range was set to 0.08 Da and retention time range to 0.1 min. Furthermore, the deconvoluted peak lists were deisotoped as follows: *m*/*z* tolerance 0.05 or 30 ppm, retention time tolerance 0.01 min, and maximum charge 2. The alignment was performed on deisotoped peak lists using the Join aligner function in MZmine2 with an *m*/*z* tolerance of 0.05 Da or 30 ppm and a retention time tolerance of 0.02 min. Weights for *m*/*z* and retention time were both set to 50. The aligned peak list was filtered to exclude peaks derived from solvent and peaks with *m*/*z* lower than 141 Da. The peak IDs were lastly reset, and the peak list was exported as an .mgf file for GNPS analysis. The reset peak list was exported as a .CSV table file to assist the creation of molecular networks.

A molecular network was created with the Feature-Based Molecular Networking (FBMN) workflow on GNPS [24,37]. The mass spectrometry data were first processed with Mzmine2 [36], and the results were exported to GNPS for FBMN analysis. The data were filtered by removing all MS/MS fragment ions within +/− 17 Da of the precursor *m*/*z*. MS/MS spectra were window-filtered by choosing only the top 6 fragment ions in the +/−50 Da window throughout the spectrum. The precursor ion mass tolerance was set to 0.02 Da, and the MS/MS fragment ion tolerance to 0.02 Da. A molecular network was then created where edges were filtered to have a cosine score above 0.7 and more than 6 matched peaks. Further, edges between two nodes were kept in the network if and only if each of the nodes appeared in each other’s respective top 10 most similar nodes. Finally, the maximum size of a molecular family was set to 100, and the lowest scoring edges were removed from molecular families until the molecular family size was below this threshold. The spectra in the network were then searched against GNPS spectral libraries [24,38]. The library spectra were filtered in the same manner as the input data. All matches kept between network spectra and library spectra were required to have a score above 0.7 and at least 6 matched peaks. The DEREPLICATOR was used to annotate MS/MS spectra [39]. The molecular networks were visualized using Cytoscape software [40].

### 4.4. Fractionation and Purification

The KC subextract of *Pyrenochaetopsis* sp. FVE-087 was fractionated on a C18-SPE column eluting with 10% stepwise gradient of MeOH in water (0–100) to yield 11 fractions (F0–F10). Anticancer bioactivity was tracked to fractions F7–F9. The bioactive fraction 8 (F8, 369.6 mg) was chromatographed by RP-HPLC equipped with an Onyx monolithic C18 column using MeCN:H_2_O mixtures (40% isocratic MeCN over 28 min and gradual increase to 60% MeCN by 40 min, flow 3.0 mL/min) to yield 14 subfractions (F8-1 to 14). Pyrenosetin E (**5**) and pyrenosetin F (**6**) were tracked to F8-8 (7.4 mg) and purified by HPLC on an analytical synergi polar-RP 80 Å LC column. The elution of the fraction with MeCN:H_2_O (55:45, flow 1.0 mL/min) yielded pyrenosetin E (**5**, 1.5 mg) and pyrenosetin F (**6**, 0.9 mg) in pure state.

*Pyrenosetin E* (**5**): colorless oil, [α]^20^_d_ -6 (*c* 0.10, MeOH); IR (oil) *v*_max_ 3419, 2951–2850 (broad), 1731, 1626, 1456, 1408, 1371, 1053, 1033 cm^−1^; ^1^H NMR (CDCl_3_, 600 MHz) and ^13^C NMR (CDCl_3_, 150 MHz), Table 1 and Table 2; HR-ESIMS found *m*/*z* [M+Na]^+^ 452.2396, C_25_H_35_NO_5_Na, calculated for 452.2407.

*Pyrenosetin F* (**6**): colorless oil, [α]^20^_d_ +67 (*c* 0.10, MeOH); IR (oil) *v*_max_ 2919–2823 (broad), 1731, 1677, 1456, 1258, 1057, 1033 cm^−1^; ^1^H NMR (CDCl_3_, 600 MHz) and ^13^C NMR (CDCl_3_, 150 MHz), Table 1 and Table 2; HR-ESIMS found *m*/*z* 428.2422 [M+H]^+^, C_25_H_34_NO_5_, calculated for 428.2431.

### 4.5. Mosher’s Reaction

Pyrenosetin E (**5**) was converted to its 16*O*-(*R*)- and 16*O*-(*S*)-MTPA esters using (*S*)- and (*R*)- MTPA chloride, respectively, as described [26]. Briefly, compound **5** (0.3 mg, 1.2 μmol) was dissolved in pyridine (0.3 mL) and reacted with (*S*)-MTPA chloride (1.4 mg, 5.5 μmol) under stirring at room temperature for 12 h (loss of one molecule of water from **5** also occurred during the reaction). After the reaction, water (1.0 mL) and CH_2_Cl_2_ (1.0 mL) were added to the reaction mixture. The organic layer was recovered and evaporated under reduced pressure. The residue was purified by RP-HPLC eluted with an MeCN/H_2_O gradient (from 50:50 to 100:0 in 5 min, 100:0 in 5 to 13 min, and from 100:0 to 50:50 in 13 to 13.4 min, flow 1.0 mL/min) to afford the dehydrated 16*O*-(*R*)-MTPA ester **5r** (0.2 mg, t_R_ 8.7 min). The dehydrated 16*O*-(*S*)-MTPA ester **5s** (0.2 mg, t_R_ 8.7 min) was obtained in the same way using (*R*)-MTPA chloride. The two esters were dissolved in CDCl_3_ and analyzed by ^1^H-NMR spectroscopy (Figures S9 and S10).

*16O-(R)-MTPA ester of dehydrated pyrenosetin E* (**5r**): colorless oil; ^1^H NMR (CDCl_3_, 600 MHz): *δ* 7.50–7.35 (5H, m, phenyl protons), 5.89 (1H, dd, *J* = 15.4 and 9.8 Hz, H-14), 5.55 (1H, dd, *J* = 15.4 and 7.2 Hz, H-15), 5.39 (1H, quintet, *J* = 6.6 Hz, H-16), 5.23 (1H, br. s, H-5), 5.21 (1H, d, *J =* 2.3 Hz, H-6’a), 4.54 (1H, d, *J =* 2.3 Hz, H-6’b), 3.49 (3H, s, OMe), 3.38 (1H, dd, *J* = 11.3 and 10.0 Hz, H-13), 3.09 (3H, s, H_3_-7’), 2.69 (1H, d, *J* = 11.5 Hz, H-3), 1.71 (3H, s, H_3_-18), 1.33 (3H, d, *J* = 6.3 Hz, H_3_-17), 1.01 (3H, s, H_3_-12), 0.90 (3H, d, *J* = 6.5 Hz, H_3_-19).

*16O-(S)-MTPA ester of dehydrated pyrenosetin E* (**5s**): colorless oil; ^1^H NMR (CDCl_3_, 600 MHz): *δ* 7.50–7.35 (5H, m, phenyl protons), 5.92 (1H, dd, *J* = 15.4 and 9.7 Hz, H-14), 5.65 (1H, dd, *J* = 15.4 and 7.0 Hz, H-15), 5.41 (1H, quintet, *J* = 6.6 Hz, H-16), 5.25 (1H, br. s, H-5), 5.19 (1H, d, *J =* 2.3 Hz, H-6’a), 4.52 (1H, d, *J =* 2.3 Hz, H-6’b), 3.45 (3H, s, OMe), 3.39 (1H, dd, *J* = 11.3 and 9.8 Hz, H-13), 3.06 (3H, s, H_3_-7’), 2.69 (1H, d, *J* = 11.5 Hz, H-3), 1.71 (3H, s, H_3_-18), 1.28 (3H, d, *J* = 6.3 Hz, H_3_-17), 1.01 (3H, s, H_3_-12), 0.90 (3H, d, *J* = 6.5 Hz, H_3_-19).

### 4.6. Computational Studies

Models of the four stereoisomers at C3’ and C5’ of pyrenosetin E (**5**) and of the four stereoisomers at C3’ and C5’ of pyrenosetin F (**6**) were generated using the Builder module in the INSIGHT II/Discover package (BIOVIA, 5005 Wateridge Vista Drive, San Diego, CA 92121, USA). A preliminary conformational search was performed on the 3’*R*,5’*R* stereoisomer of pyrenosetin E using a described protocol [41] based on molecular dynamics (MD). Briefly, a 10-ns MD simulation was performed at 300 K in the CFF91 force field, giving 200 structures that were optimized in the same force field. Examination of the resulting structures showed that the tetracyclic nucleus of pyrenosetins is substantially rigid, with the *trans*-decalin in the chair/chair conformation, the tetramic acid nearly planar, and the cyclopentenone ring in a twist conformation with C-13 above the plane and C-3 below the plane; conformational changes only involved the two side chains C-14/C-17 and C-5’/C-6’.

Therefore, a set of conformers was generated for each stereoisomer using the Search Compare module of the INSIGHT II/Discover package, using the determined conformation of the tetracyclic nucleus (or its analogue with C-3’ inverted) as a starting point and varying systematically the dihedral angles around the C-13/C-14, C-15/C-16, C-16/O-16, C-5’/C-6’, and C-6’/O-6’ bonds. The eight sets of conformers generated in this way were used as input structures for density functional theory (DFT) calculations.

DFT calculations were performed using the program Gaussian 16 (Revision C.01, Gaussian Inc., Wallingford CT, USA). Structure optimization was performed at the B3LYP/6-31G(d,p) level of theory, using the SMD solvent model (CHCl_3_); Cartesian coordinates of the optimized structures of the six lowest-energy conformers of each of the stereoisomers studied are reported in Tables S6–S13. Prediction of NMR chemical shifts was achieved using the Gauge Invariant Atomic Orbitals (GIAO) method at the PBE0/6-311+G(d,p) level of theory, using the PCM solvent model for CHCl_3_; the results are reported in Tables S2 and S3. The weighted mean of the isotropic shieldings of individual conformers was calculated using Boltzmann statistics (*T* = 300 K) based on the Gibbs free energy of conformers. This was calculated by adding the thermal free energy obtained from a vibrational frequency calculation at the B3LYP/6-31G(d,p)/SMD level of theory and the electronic energy from a single point calculation at the higher B3LYP/TZVP/SMD level of theory. The results are reported in Tables S4 and S5.

### 4.7. Bioactivity Assessments

The bioactivity of the crude extracts, the subextracts and the SPE fractions of the KC subextracts from both *Pyrenochaetopsis* sp. were tested against five human cancer cell lines and the non-cancerous human keratinocyte line HaCaT (CLS, Eppelheim, Germany) as previously described [16]. For monitoring the isolations, malignant melanoma cell line A-375 (CLS, Eppelheim, Germany) was used. The bioassay results were evaluated by monitoring the metabolic activity using the CellTiterBlue Cell Viability Assay (Promega, Mannheim, Germany). The HaCaT cells were grown in RPMI medium, and A-375 cells in DMEM medium supplemented with 4.5 g/L D-Glucose and 110 mg/L sodium pyruvate. All media were supplemented with L-glutamine, 10% fetal bovine serum, 100 U/mL penicillin, and 100 mg/mL streptomycin. The cultures were maintained at 37 °C under a humidified atmosphere and 5% CO_2_. The cell lines were transferred every 3 or 4 days. For the experimental procedure, the cells were seeded in 96-well plates at a concentration of 10,000 cells per well. A stock solution of 40 mg/mL in DMSO was prepared for each extract. After 24 h incubation, the medium was removed from the cells, and 100 μL fresh medium containing the test samples was added. Each sample was prepared in duplicate once. Doxorubicin was used as a positive control, and 0.5% DMSO and growth media were used as negative controls. Following compound addition, plates were cultured at 37 °C for 24 h. Afterwards, the assay was performed according to the manufacturer’s instructions and measured using the microplate reader Tecan Infinite M200 at excitation of 560 nm and emission of 590 nm. For determination of the IC_50_ values of the pure compounds, a dilution series of the pure compounds was prepared and tested, as described before for the crude extract and fractions [10,16,17]. The IC_50_ values were calculated by Excel as the concentration that shows 50% inhibition of viability based on a negative control (no compound) and compared with the positive control (doxorubicin).

## Figures and Tables

**Table 1:** ^1^H NMR data of compounds **5** and **6**, in comparison to **2** and **3** (600 MHz, CDCl_3_).

C	Pyrenosetin E (5)	Pyrenosetin B (2)	Pyrenosetin F (6)	Pyrenosetin C (3)
	*δ*_H_, *J* in Hz	*δ*_H_, *J* in Hz	*δ*_H_, *J* in Hz	*δ*_H_, *J* in Hz
1	-	-	-	-
2	-	-	-	-
3	2.57, d (11.5)	2.73, d (11.4)	2.82, d (11.3)	2.66, d (11.3)
4	-	-	-	-
5	5.22, br s	5.22, br s	5.26, br s	5.28, br s
6	1.82, m	1.82, m	1.85, m	1.83, m
7 eq	1.79, m	1.80, m	1.82, m	1.82, m
7ax	0.85, q (11.9)	0.84, m	0.86, q (12.0)	0.88, m
8	1.42, m	1.44, m	1.45, m	1.44, m
9 eq	1.72, m	1.73, m	1.74, m	1.73, m
9 ax	0.97, dq (3.3, 12.2)	0.93, m	0.92, q (3.5, 12.4)	0.99, m
10 eq	1.43, m	1.40, m	1.37, m	1.41, m
10 ax	1.05, dq (3.0, 12.5)	1.07, dq (12.8, 3.4)	1.08, dq (3.3, 12.6)	1.04, m
11	1.56, dt (2.9, 12.0)	1.42, m	1.41, m	1.64, td (11.0, 2.7)
12	0.98, s	1.00, s	1.02, s	1.01, s
13	3.42, dd (11.5, 9.3)	3.26, dd (11.4, 9.4)	3.40, dd (11.3, 9.5)	3.57, dd (11.4, 9.8)
14	5.82, dd (15.4, 9.3)	5.97, dd (15.3, 9.4)	7.09, dd (16.1, 9.5)	6.85, dd (15.9, 9.8)
15	5.55, dd (15.4, 7.5)	5.50, dd (15.3, 8.0)	6.02, d (16.1)	6.18, d (15.9)
16	4.17, quintet (6.6)	4.18, m	-	-
17	1.18, d (6.3)	1.19, d (6.2)	2.22, s	2.22, s
18	1.70, s	1.69, br s	1.69, s	1.68, br s
19	0.89, d (6.5)	0.91, d (6.5)	0.91, d (6.5)	0.90, d (6.2)
2′	-	-	-	-
3′	-	-	-	-
4′	-	-	-	-
5′	3.85, m	3.94, dd (2.7, 1.9)	4.00, t (2.6)	3.61, dd (4.9, 2.7)
6′ a	4.10, br. d (11.7)	4.08, m	4.03, d (11.3)	4.10, m
6′ b	3.87, m	3.86, dd (12.4, 2.7)	3.84, d (11.3)	3.94, m
7′	3.09, s	3.07, s	3.06, s	3.11, s

**Table 2:** ^13^C NMR data of 5 and 6, compared to pyrenosetins B (2) and C (3) (150 MHz, CDCl_3_).

C	Pyrenosetin E (5) *δ*_C_	Pyrenosetin B (2) *δ*_C_	Pyrenosetin F (6) *δ*_C_	Pyrenosetin C (3) *δ*_C_
1	209.6 (C)	209.8 (C)	208.5 (C)	212.1 (C)
2	54.3 (C)	54.1 (C)	54.1 (C)	54.7 (C)
3	53.4 (CH)	52.8 (CH)	52.9 (CH)	53.6 (CH)
4	132.1 (C)	132.3 (C)	131.5 (C)	130.9 (C)
5	127.8 (CH)	127.6 (CH)	128.4 (CH)	128.8 (CH)
6	37.6 (CH)	37.6 (CH)	37.7 (CH)	37.6 (CH)
7	42.0 (CH_2_)	42.0 (CH_2_)	41.9 (CH_2_)	41.8 (CH_2_)
8	33.0 (CH)	32.9 (CH)	32.9 (CH)	32.9 (CH)
9	35.4 (CH_2_)	35.3 (CH_2_)	35.3 (CH_2_)	35.2 (CH_2_)
10	25.3 (CH_2_)	25.3 (CH_2_)	25.3 (CH_2_)	25.2 (CH_2_)
11	37.8 (CH)	38.0 (CH)	38.0 (CH)	37.4 (CH)
12	15.4 (CH_3_)	15.2 (CH_3_)	15.3 (CH_3_)	15.2 (CH_3_)
13	49.4 (CH)	51.0 (CH)	50.0 (CH)	50.6 (CH)
14	131.2 (CH)	130.5 (CH)	146.1 (CH)	144.4 (CH)
15	136.8 (CH)	137.8 (CH)	134.1 (CH)	133.9 (CH)
16	69.0 (CH)	69.0 (CH)	198.8 (C)	197.6 (C)
17	22.8 (CH_3_)	22.9 (CH_3_)	26.8 (CH_3_)	27.6 (CH_3_)
18	23.9 (CH_3_)	23.9 (CH_3_)	23.8 (CH_3_)	23.7 (CH_3_)
19	22.4 (CH_3_)	22.4 (CH_3_)	22.4 (CH_3_)	22.4 (CH_3_)
2′	169.1 (C)	168.6 (C)	167.7 (C)	167.8 (C)
3′	74.4 (C)	73.8 (C)	73.7 (C)	72.7 (C)
4′	204.8 (C)	205.0 (C)	204.1 (C)	206.4 (C)
5′	68.8 (CH)	69.4 (CH)	69.1 (CH)	69.8 (CH)
6′	57.9 (CH_2_)	58.3 (CH_2_)	58.4 (CH_2_)	60.3 (CH_2_)
7′	28.0 (CH_3_)	27.7 (CH_3_)	27.8 (CH_3_)	28.5 (CH_3_)

## Data Availability

The data may be available from the corresponding author.

## Supplementary Materials

The following are available online at https://www.mdpi.com/article/10.3390/md20030210/s1. Table S1: MN-based dereplication of the *Pyrenochaetopsis* sp. FVE-001 and FVE-087 KC subextracts. Tables S2–S13: Data regarding computational studies on compounds **5** and **6**. Figures S1 and S2: Global molecular network analyses and Euler diagram of KC subextracts of *Pyrenochaetopsis* strains. Figures S3–S20: Spectroscopic data for **5** and **6**. Figures S21–S23: DP4+ results for compounds **5**, **6** and **3**.
