# Supplementary material for: Application of Feature-Based Molecular Networking for Comparative Metabolomics and Targeted Isolation of Stereoisomers from Algicolous Fungi
Source: Mar Drugs. 2022 Mar 16;20(3):210. doi: 10.3390/md20030210 (PMC8948805; doi:10.3390/md20030210)
Supplement: Supplementary file 1 [file marinedrugs-20-00210-s001.zip › marinedrugs-1620886-supplementary.pdf]

Supplementary Information

Application of Feature-Based Molecular Networking for Comparative Metabolomics and Targeted Isolation of Stereoisomers from Algicolous Fungi

Bicheng Fan [1], Laura Grauso [2], Fengjie Li [1], Silvia Scarpato [3], Alfonso Mangoni[3], and Deniz Tasdemir [1,4]*

[1] GEOMAR Centre for Marine Biotechnology (GEOMAR-Biotech), Research Unit Marine Natural Products Chemistry, GEOMAR Helmholtz Centre for Ocean Research Kiel, Am Kiel-Kanal 44, 24106 Kiel, Germany; bichengfan@hotmail.com (B.F.); Fengjieli0620@hotmail.com (F.L.)

[2] Dipartimento di Agraria, Università degli Studi di Napoli Federico II, 80055 Portici, Italy; laura.grauso@unina.it

[3] Dipartimento di Farmacia, Università degli Studi di Napoli Federico II, 80131 Napoli, Italy; silvia.scarpato@unina.it (S.S.); alfonso.mangoni@unina.it (A.M.)

[4] Faculty of Mathematics and Natural Sciences, Kiel University, Christian-Albrechts-Platz 4, 24118 Kiel, Germany

* Correspondence: dtasdemir@geomar.de; Tel.: +49-431-600-4430

List of Figures:

Figure S1. Global molecular networking of KC subextracts of *Pyrenochaetopsis* sp. strain FVE-001 (grey) and *Pyrenochaetopsis* sp. strain FVE-087 (red). **A**). Cluster annotated as decalin derivative containing pyrenosetins and phomasetin. **B**). Cluster annotated as decalin derivative containing wakodecalines.

Figure S2. Euler diagram based on the global molecular network (MN in Figure S1). Grey: ions observed in the KC subextract of *Pyrenochaetopsis* sp. FVE-001. Red: ions observed in the KC subextract of *Pyrenochaetopsis* sp. FVE-087.

Figure S3. ^{1}H NMR spectrum of compound **5** (600 MHz, $CDCl_3$).

Figure S4. ^{13}C NMR spectrum of compound **5** (150 MHz, $CDCl_3$).

Figure S5. DEPT-HSQC spectrum of compound **5** (600M Hz, $CDCl_3$).

Figure S6. COSY spectrum of compound **5** (600 MHz, $CDCl_3$).

Figure S7. HMBC spectrum of compound **5** (600 MHz, $CDCl_3$).

Figure S8. NOESY spectrum of compound **5** (600 MHz, $CDCl_3$).

Figure S9. ^{1}H NMR spectrum of 16-(*R*)-MTPA ester of **5** (600M Hz, $CDCl_3$).

Figure S10. ^{1}H NMR spectrum of 16-(*S*)-MTPA ester of **5** (600M Hz, $CDCl_3$).

Figure S11. HR-ESIMS of compound **5.**

Figure S12. FT-IR spectrum of compound **5.**

Figure S13. ^{1}H NMR spectrum of compound **6** (600 MHz, $CDCl_3$).

Figure S14. ^{13}C NMR spectrum of compound **6** (150 MHz, $CDCl_3$).

Figure S15. DEPT-HSQC spectrum of compound **6** (600M Hz, $CDCl_3$).

Figure S16. COSY spectrum of compound **6** (600M Hz, $CDCl_3$).

Figure S17. HMBC spectrum of compound **6** (600M Hz, $CDCl_3$).

Figure S18. NOESY spectrum of compound **6** (600M Hz, $CDCl_3$).

Figure S19. HR-ESIMS spectrum of compound **6**.

Figure S20. FT-IR spectrum of compound **6**.

Figure S21. DP4+ results for compound **5**.

Figure S22. DP4+ results for compound **6**.

Figure S23. DP4+ results for compound **3**.

List of Tables:

Table S1. Putatively identified compounds by MN-based dereplication of KC subextracts of *Pyrenochaetopsis* sp. strains FVE-001 and FVE-087. Annotation considered the putative ID, retention time (t_R), *m/z* value, adduct, chemical structure, molecular formula, and MS/MS fragments.

Table S2. Experimental ^{13}C and ^{1}H NMR chemical shifts of pyrenosetins E (**5**) and Boltzmann-averaged ^{13}C and ^{1}H NMR isotropic shielding values of their four possible stereoisomers at C-3' and C-5'.

Table S3. Experimental ^{13}C and ^{1}H NMR chemical shifts of pyrenosetins F (**6**) and C (**3**) and Boltzmann-averaged ^{13}C and ^{1}H NMR isotropic shielding values of their four possible stereoisomers at C-3' and C-5'.

Table S4. Theoretical relative Gibbs free energies (kcal/mol) calculated at the B3LYP/TZVP/SMD($CHCl_3$)//B3LYP/6-31+G(d,p)/SMD($CHCl_3$) level, and populations according to the Boltzmann distribution (300 K) of populated (>1%) conformers of the four stereoisomers of pyrenosetins E (**5**) considered in this study.

Table S5. Theoretical relative Gibbs free energies (kcal/mol) calculated at the B3LYP/TZVP/SMD($CHCl_3$)//B3LYP/6-31+G(d,p)/SMD($CHCl_3$) level, and populations according to the Boltzmann distribution (300 K) of populated (>1%) conformers of the four stereoisomers of pyrenosetins F (**6**) considered in this study.

Table S6. Cartesian coordinates of the six lowest-energy conformers of the 3'*R*,5'*R* stereoisomer of pyrenosetins E at the B3LYP/TZVP/SMD($CHCl_3$)//B3LYP/6-31+G(d,p)/SMD($CHCl_3$) level.

Table S7. Cartesian coordinates of the six lowest-energy conformers of the 3'*S*,5'*R* stereoisomer of pyrenosetins E at the B3LYP/TZVP/SMD($CHCl_3$)//B3LYP/6-31+G(d,p)/SMD($CHCl_3$) level.

Table S8. Cartesian coordinates of the six lowest-energy conformers of the 3'*R*,5'*S* stereoisomer of pyrenosetins E at the B3LYP/TZVP/SMD($CHCl_3$)//B3LYP/6-31+G(d,p)/SMD($CHCl_3$) level.

Table S9. Cartesian coordinates of the six lowest-energy conformers of the 3'*S*,5'*S* stereoisomer of pyrenosetins E at the B3LYP/TZVP/SMD($CHCl_3$)//B3LYP/6-31+G(d,p)/SMD($CHCl_3$) level.

Table S10. Cartesian coordinates of the six lowest-energy conformers of the 3'*R*,5'*R* stereoisomer of pyrenosetins F at the B3LYP/TZVP/SMD($CHCl_3$)//B3LYP/6-31+G(d,p)/SMD($CHCl_3$) level.

Table S11. Cartesian coordinates of the six lowest-energy conformers of the 3'*S*,5'*R* stereoisomer of pyrenosetins F at the B3LYP/TZVP/SMD($CHCl_3$)//B3LYP/6-31+G(d,p)/SMD($CHCl_3$) level.

Table S12. Cartesian coordinates of the six lowest-energy conformers of the 3'*R*,5'*S* stereoisomer of pyrenosetins F at the B3LYP/TZVP/SMD($CHCl_3$)//B3LYP/6-31+G(d,p)/SMD($CHCl_3$) level.

Table S13. Cartesian coordinates of the six lowest-energy conformers of the 3'*S*,5'*S* stereoisomer of pyrenosetins F at the B3LYP/TZVP/SMD($CHCl_3$)//B3LYP/6-31+G(d,p)/SMD($CHCl_3$) level.

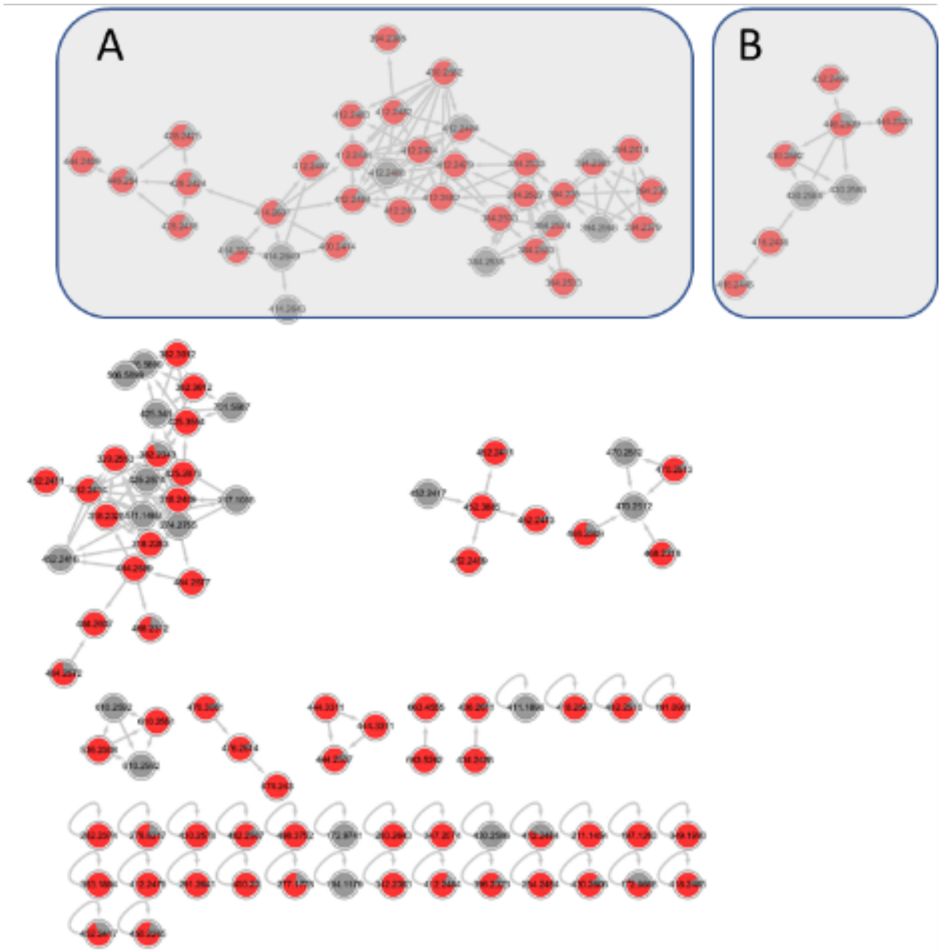

Figure S1. Global molecular networking of KC subextracts of *Pyrenochaetopsis* sp. strain FVE-001 (grey) and *Pyrenochaetopsis* sp. strain FVE-087 (red). **A**). Cluster annotated as decalin derivative containing pyrenosetins and phomasetin. **B**). Cluster annotated as decalin derivative containing wakodecalines.

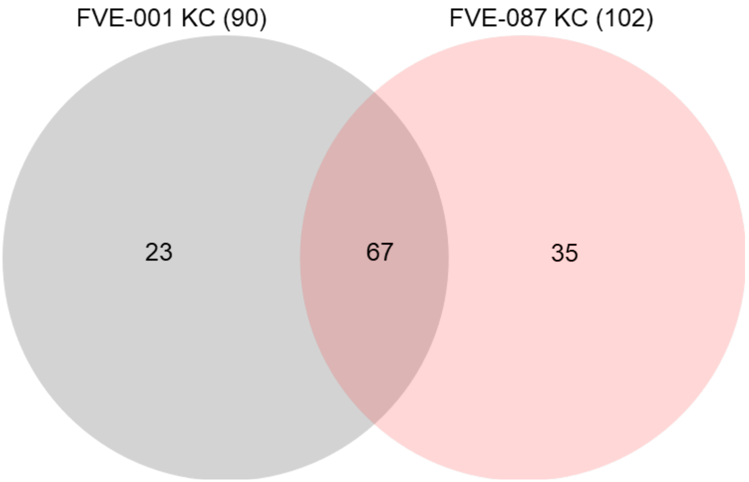

Figure S2. Euler diagram based on the global molecular network (MN in Figure S1). Grey: ions observed in the KC subextract of *Pyrenochaetopsis* sp. FVE-001. Red: ions observed in the KC subextract of *Pyrenochaetopsis* sp. FVE-087.

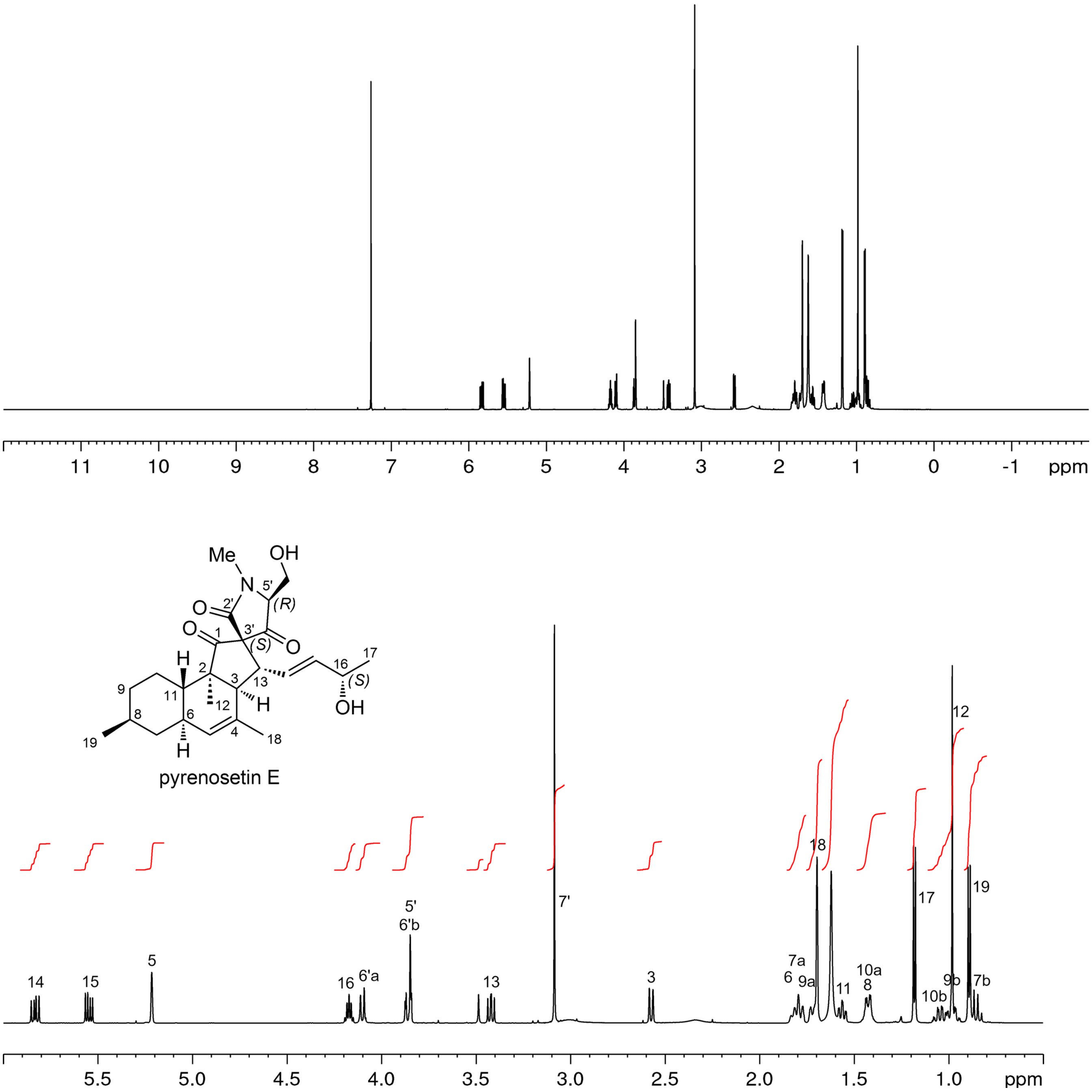

Figure S3. ^{1}H NMR spectrum of pyrenosetin E (**5**) (600 MHz, $CDCl_3$)

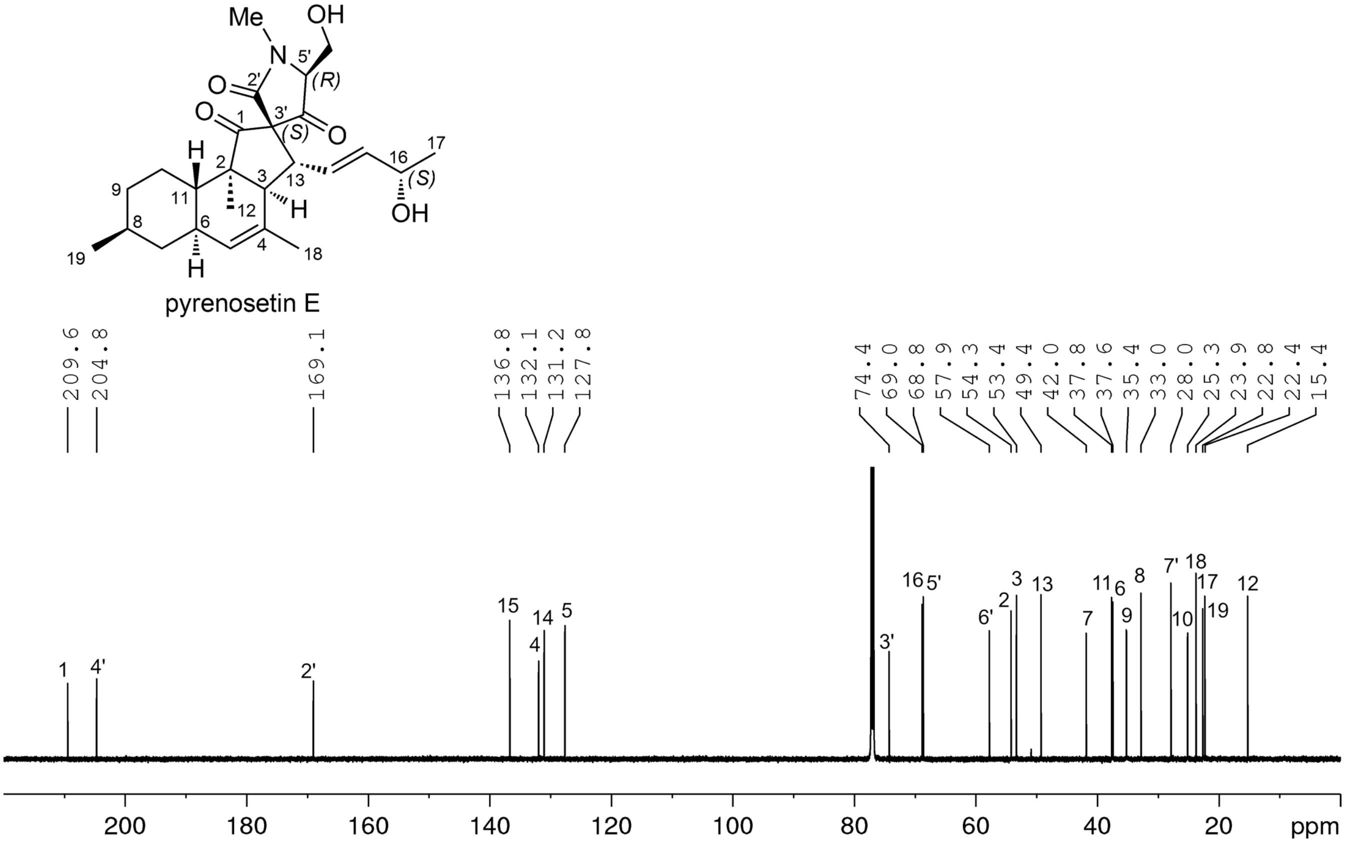

Figure S4. ^{13}C NMR spectrum of pyrenosetin E (**5**) (150 MHz, $CDCl_3$)

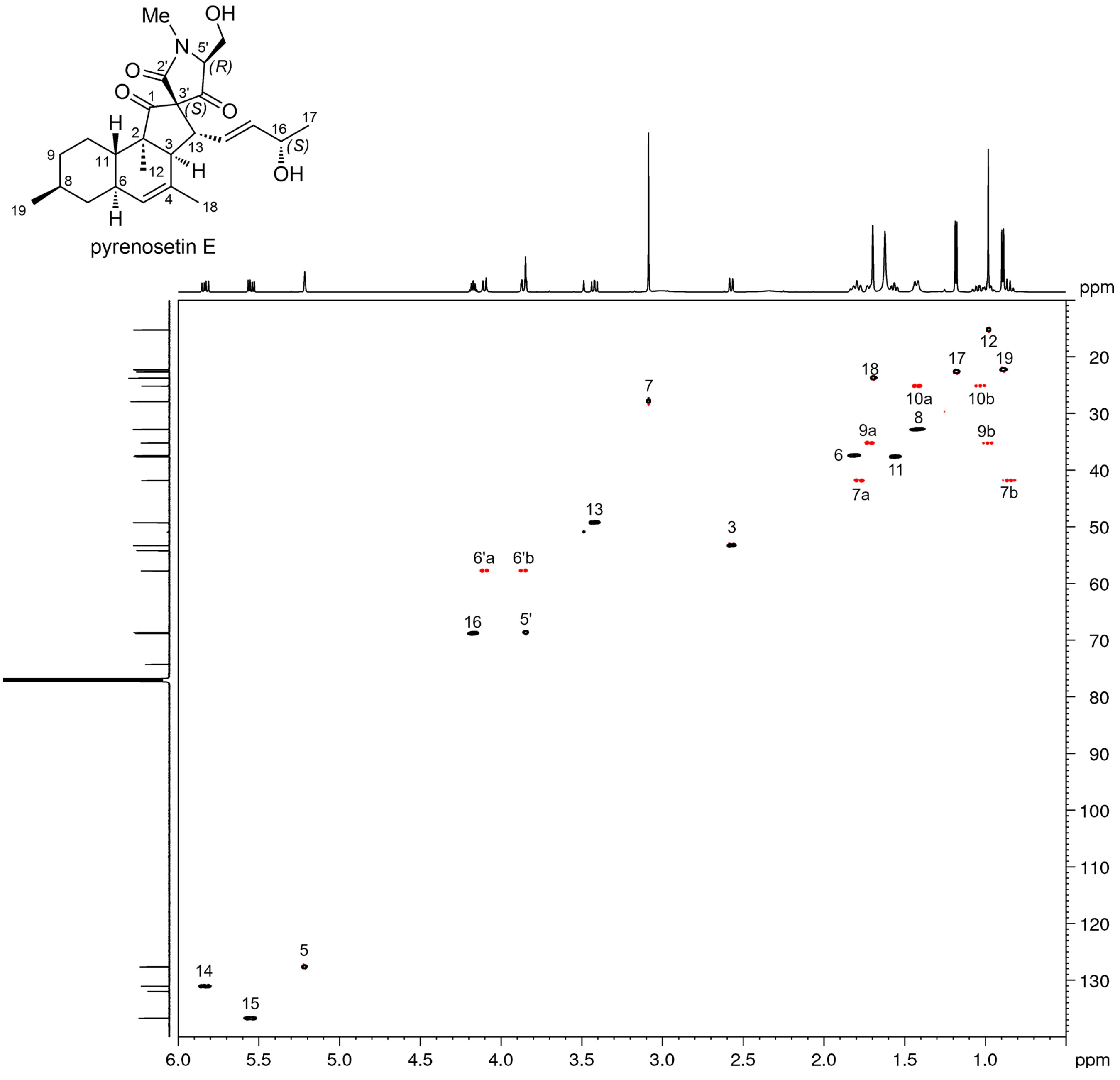

Figure S5. DEPT-HSQC spectrum of pyrenosetin E (**5**) (600 MHz, $CDCl_3$)

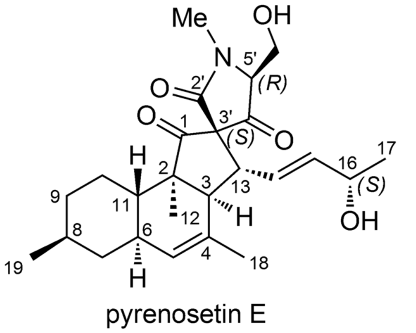

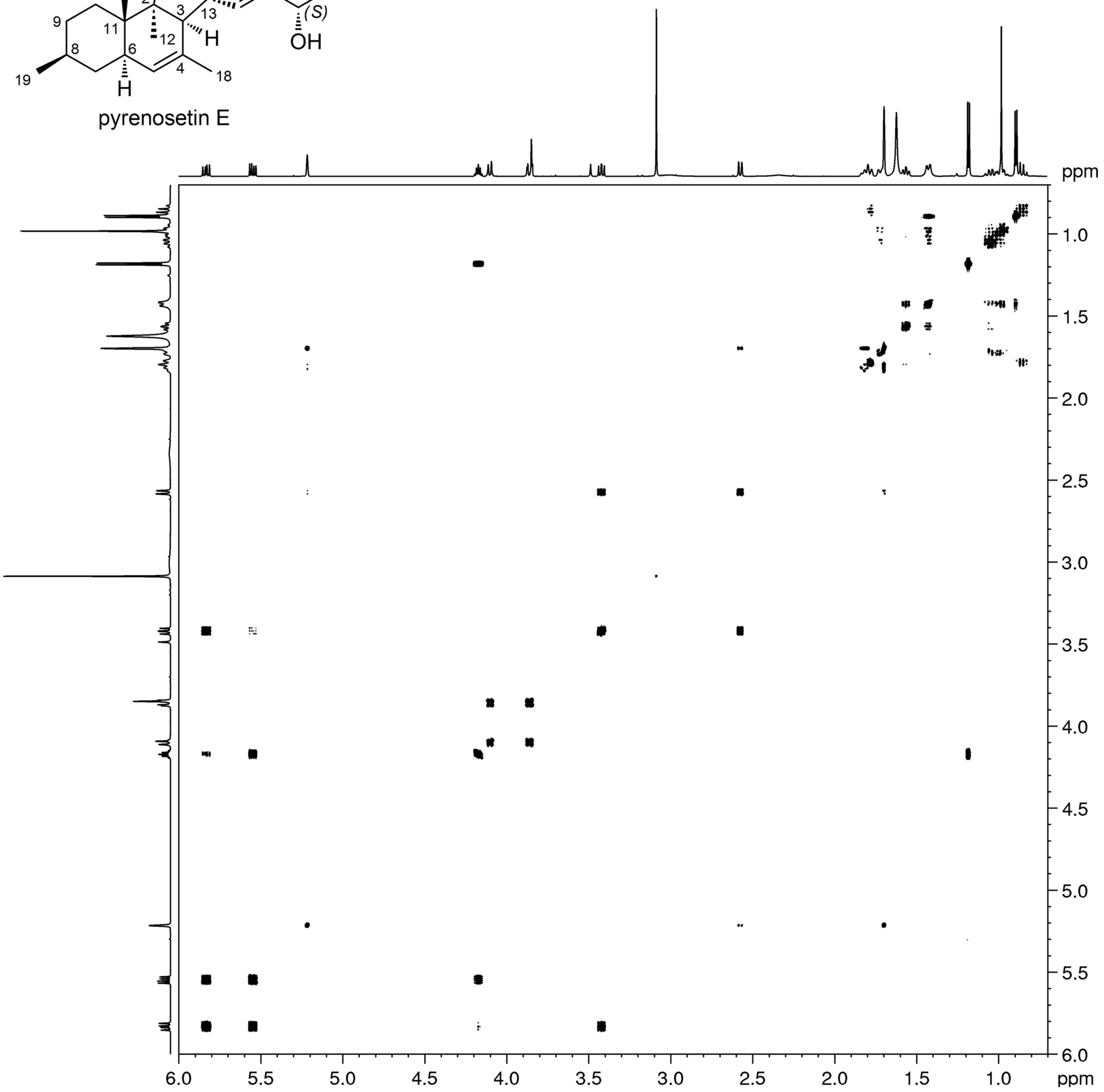

Figure S6. COSY spectrum of pyrenosetin E (**5**) (600 MHz, $CDCl_3$)

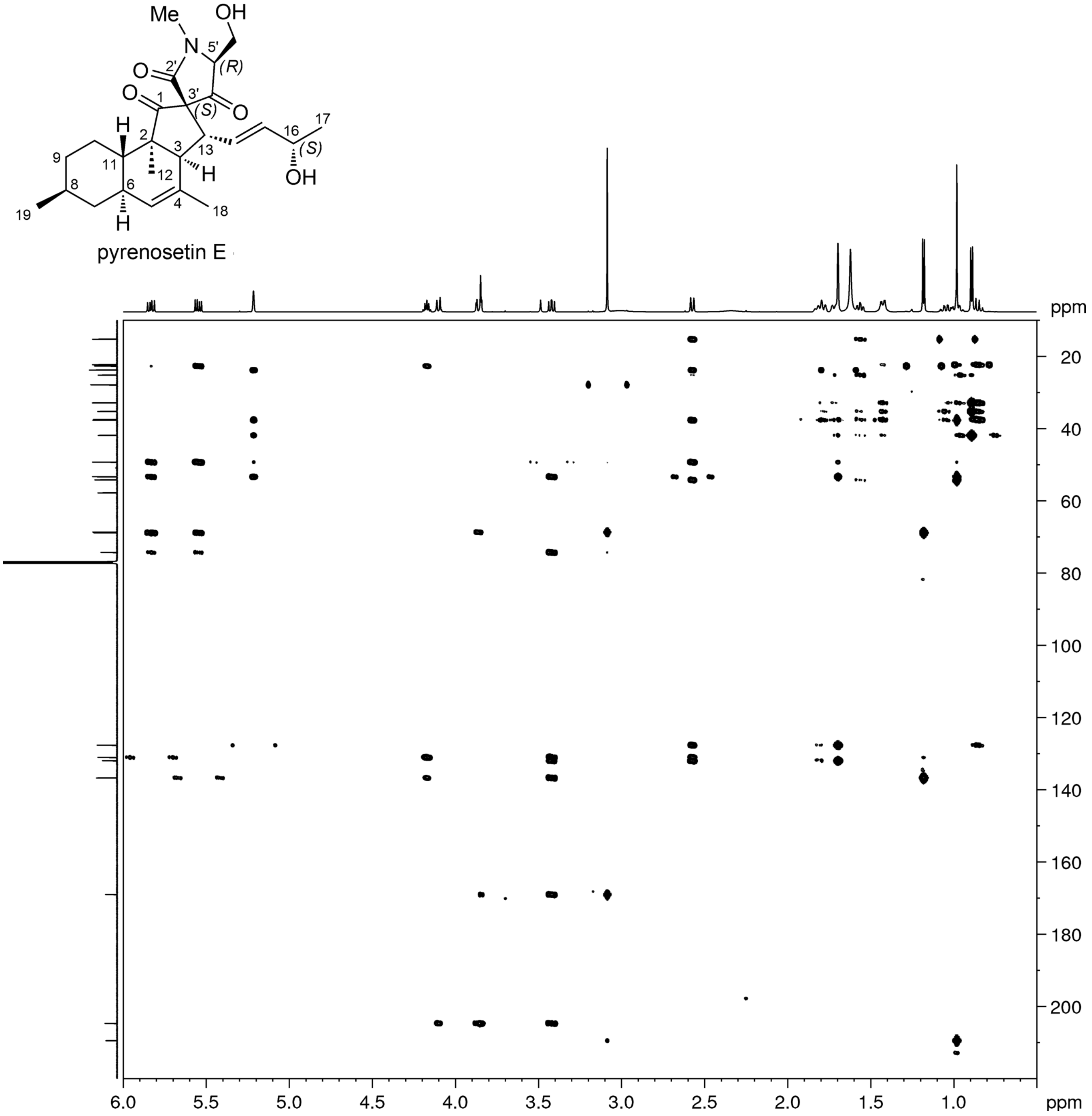

Figure S7. HMBC spectrum of pyrenosetin E (**5**) (600 MHz, $CDCl_3$)

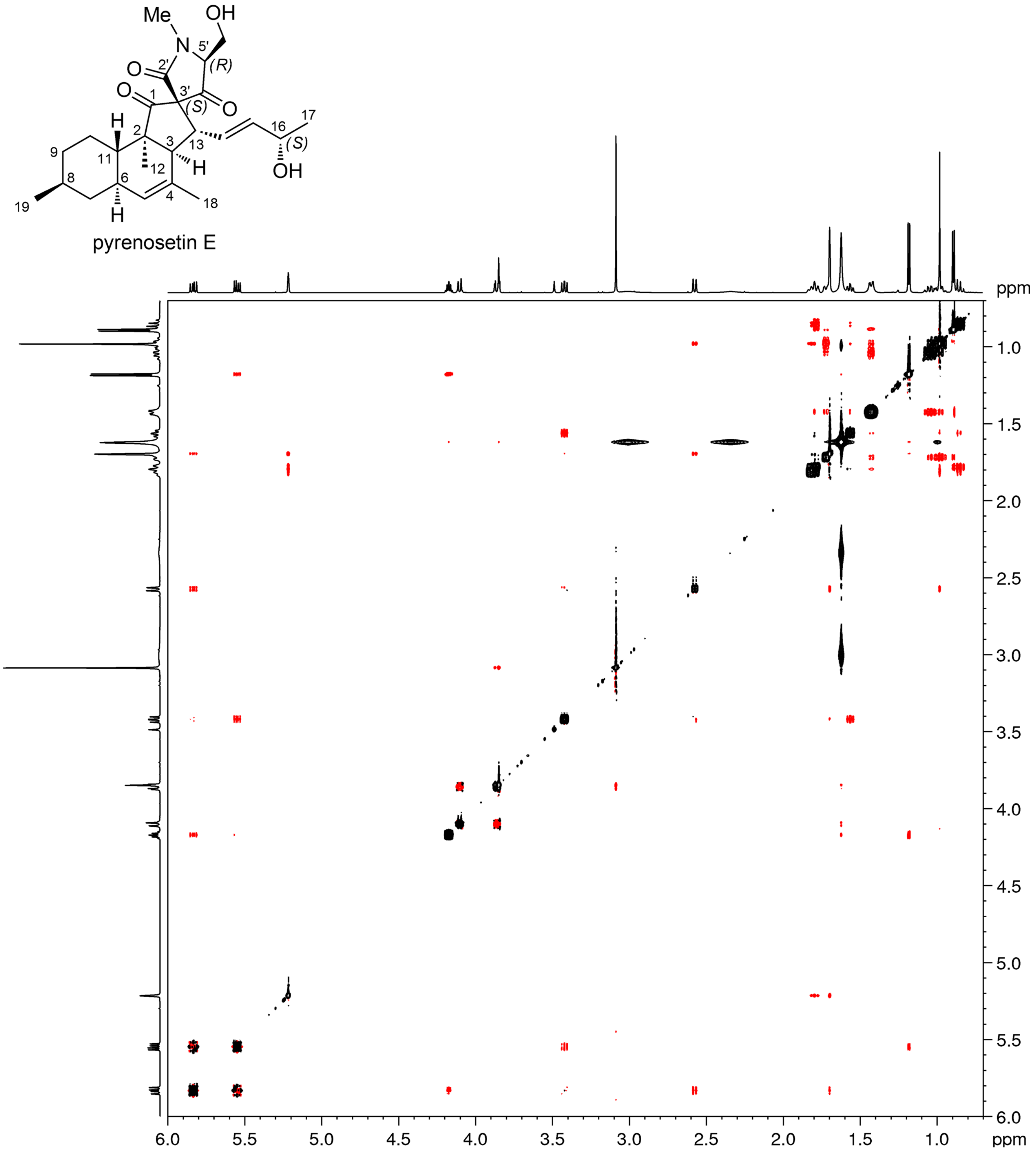

Figure S8. NOESY spectrum of pyrenosetin E (**4**) (600 MHz, $CDCl_3$)

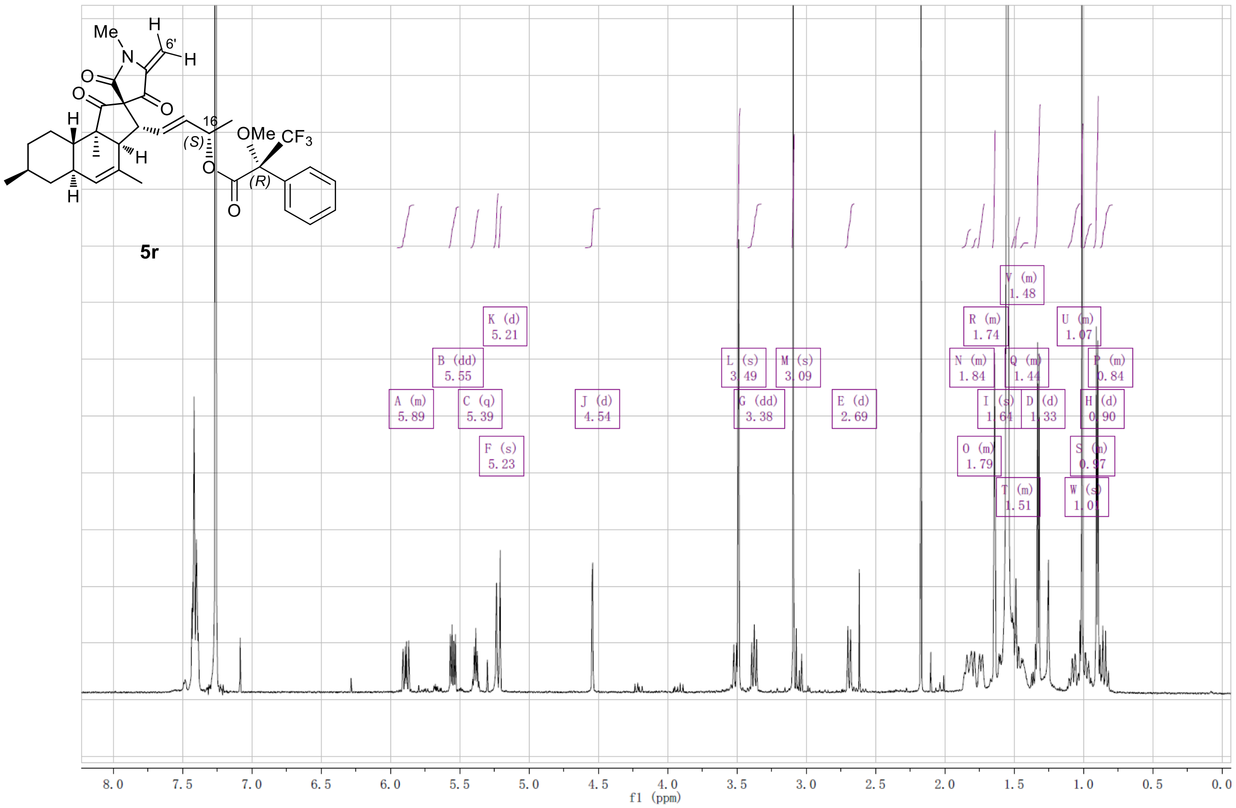

Figure S9. ^{1}H NMR spectrum of 16*O*-(*R*)-MTPA ester of dehydrated pyrenosetin E (**5r**) (600M Hz, $CDCl_3$)

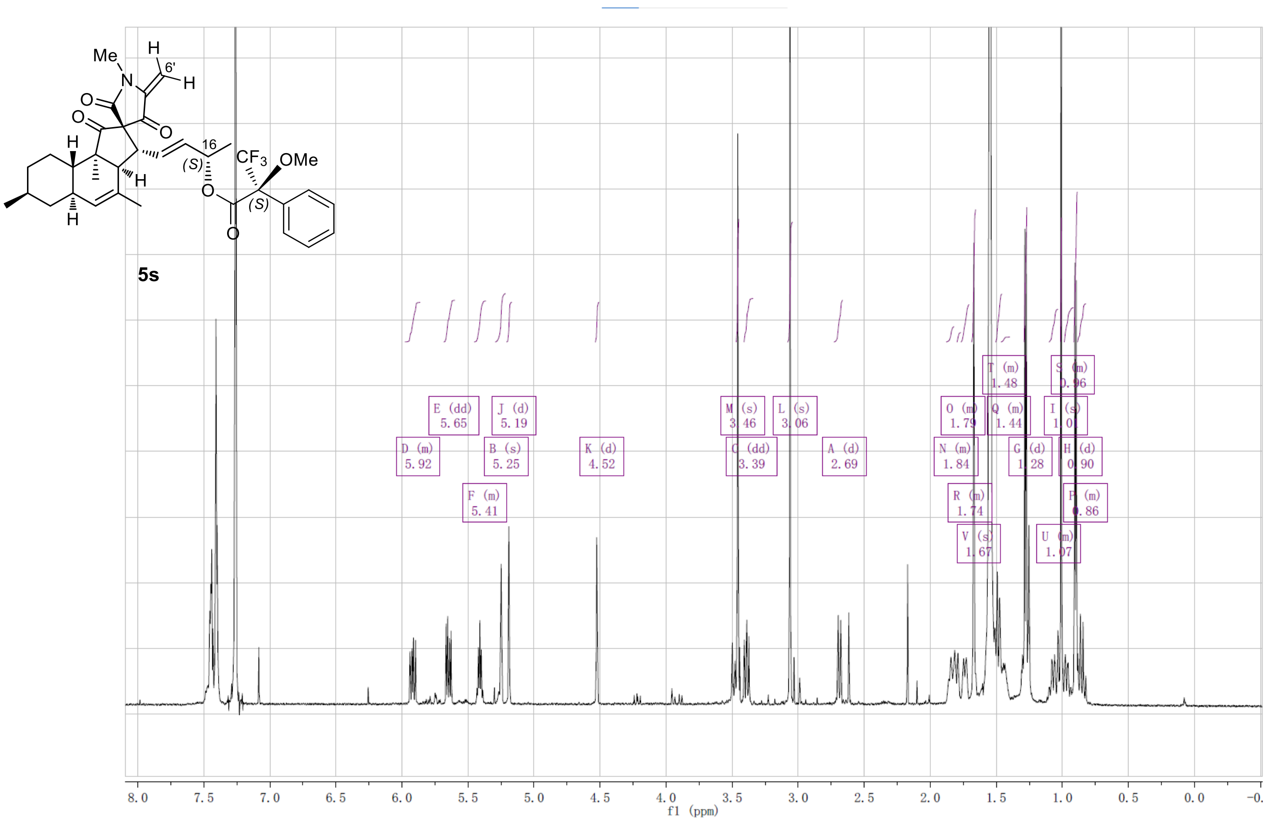

Figure S10. ^{1}H NMR spectrum of 16*O*-(*S*)-MTPA ester of dehydrated pyrenosetin E (**5s**) (600MHz, $CDCl_3$)

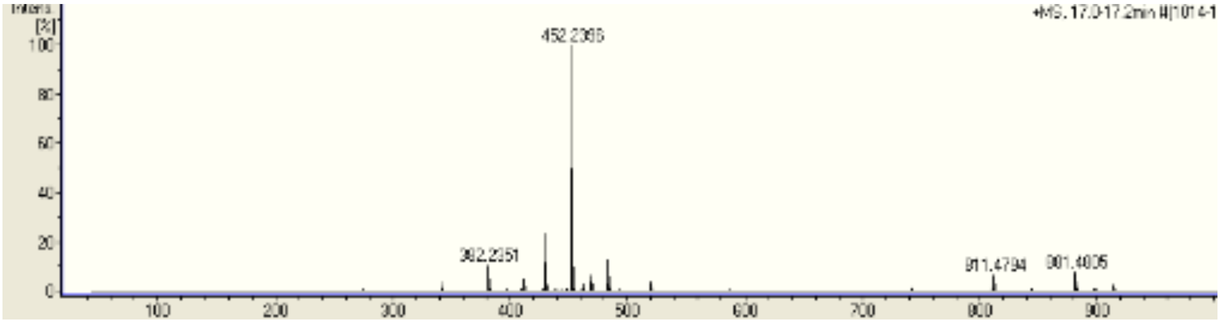

Figure S11. High-resolution ESI mass spectrum of pyrenosetin E (**5**) (positive ion mode).

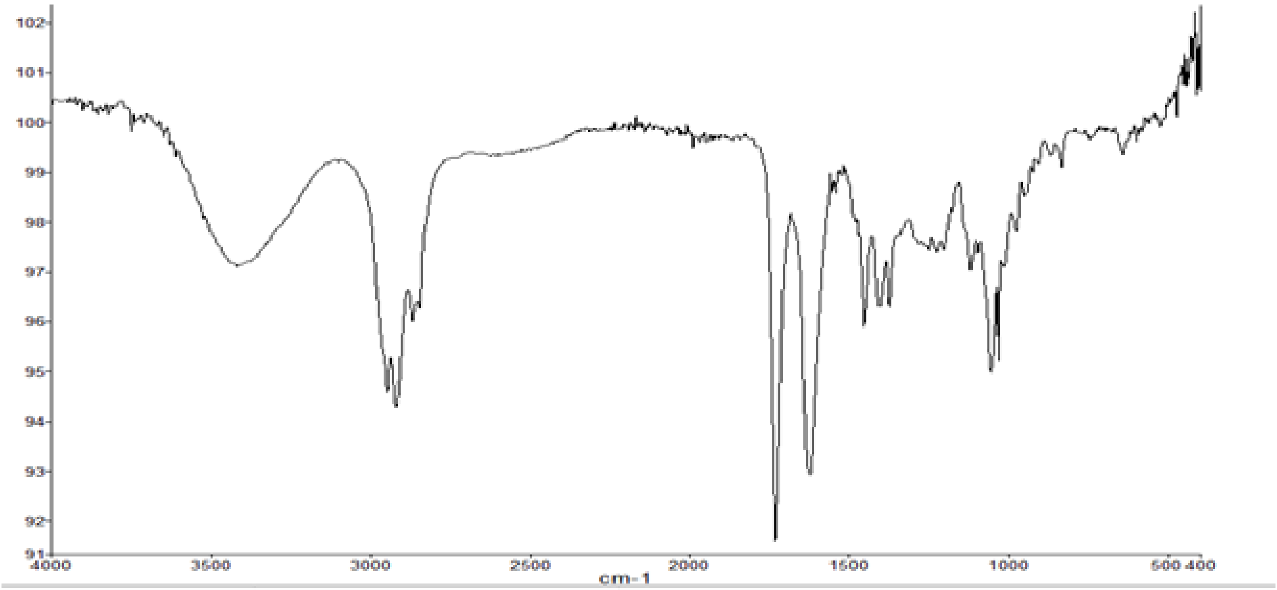

Figure S12. FT-IR spectrum of pyrenosetin E (**5**)

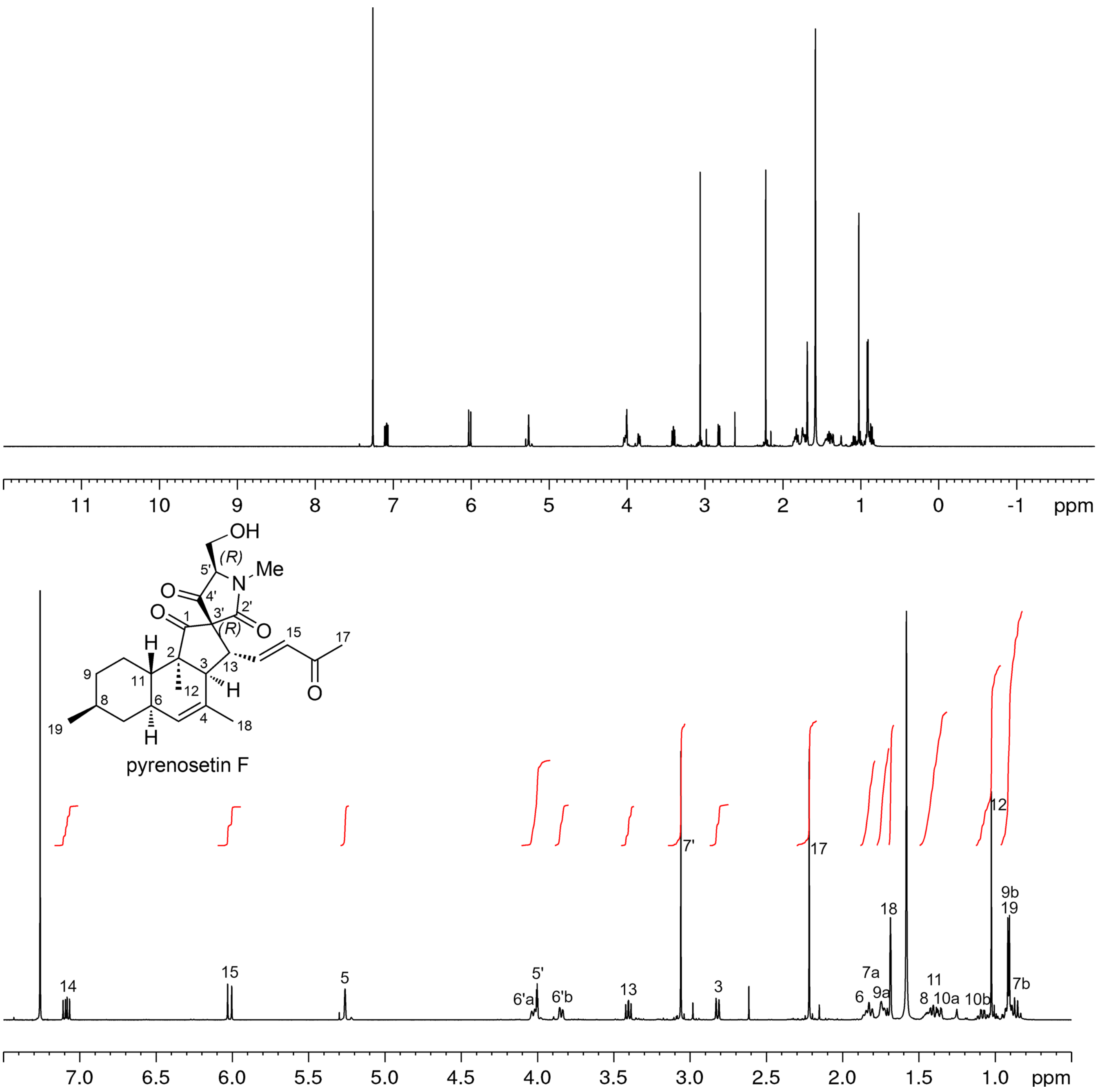

Figure S13. ^{1}H NMR spectrum of pyrenosetin F (**6**) (600 MHz, $CDCl_3$)

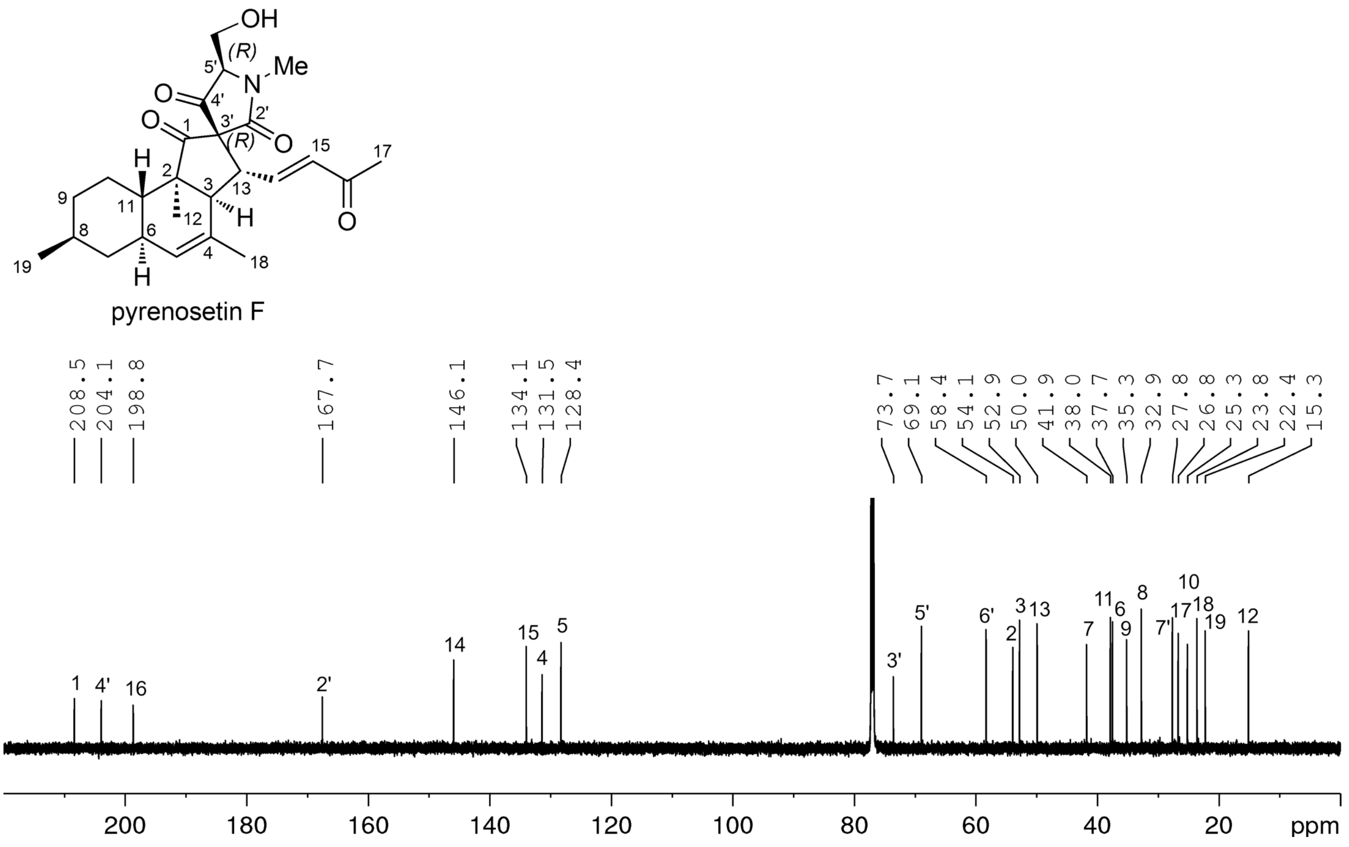

Figure S14. ^{13}C NMR spectrum of pyrenosetin F (**6**) (150 MHz, $CDCl_3$)

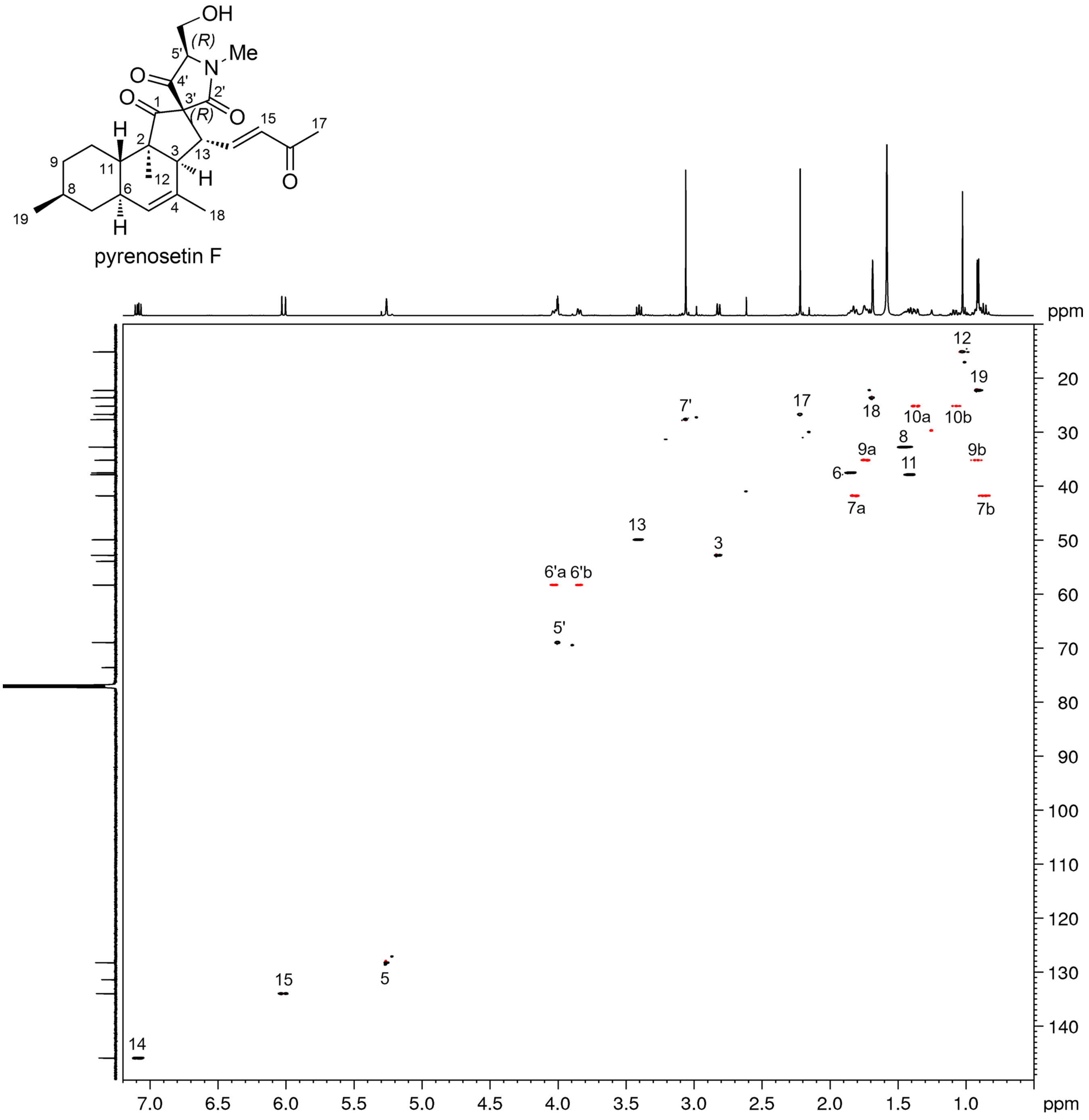

Figure S15. DEPT-HSQC spectrum of pyrenosetin F (**6**) (600 MHz, $CDCl_3$)

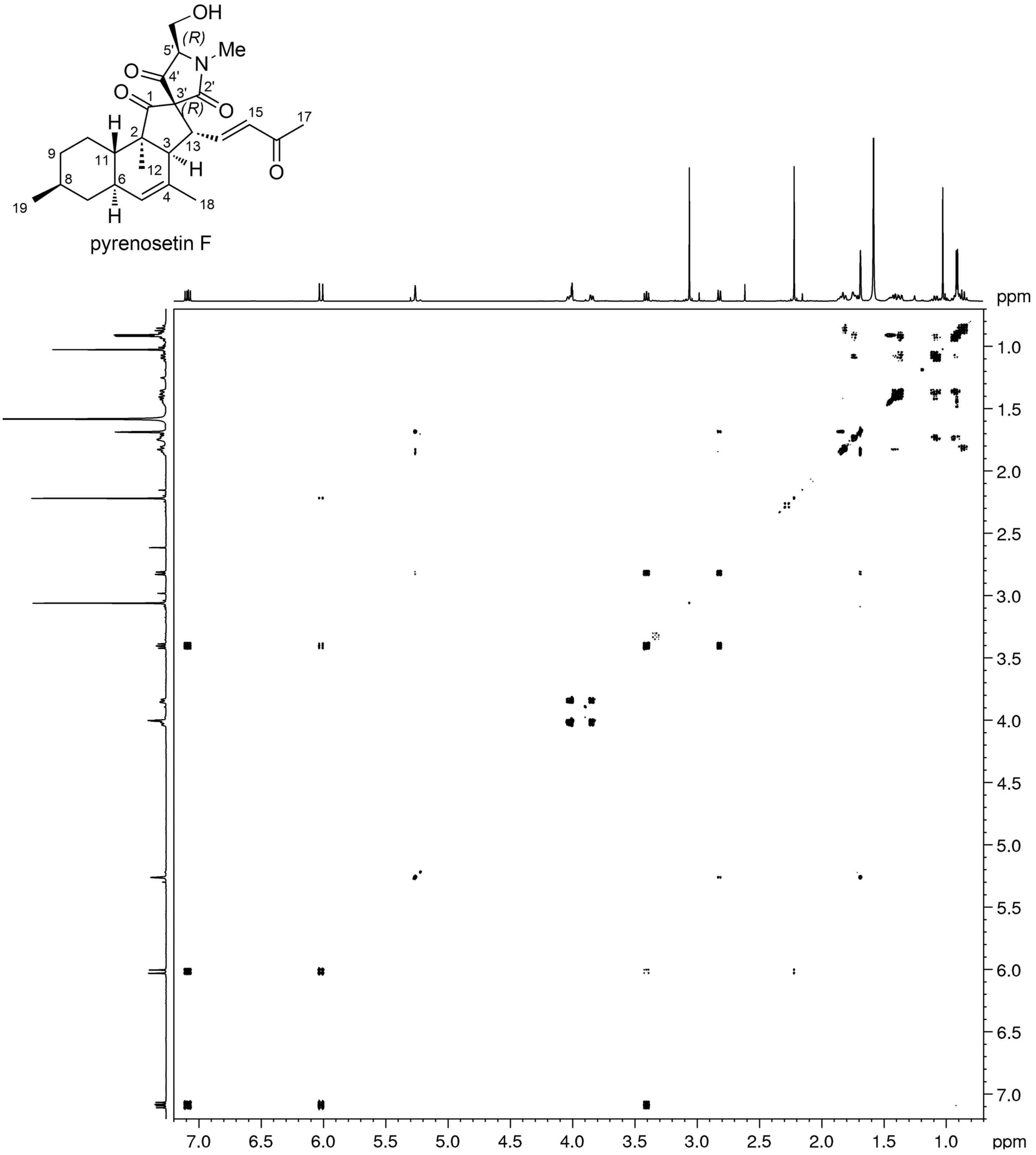

Figure S16. COSY spectrum of pyrenosetin F (**6**) (600 MHz, $CDCl_3$)

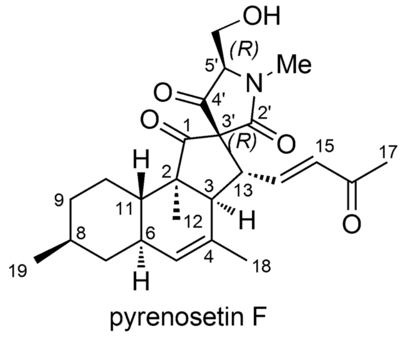

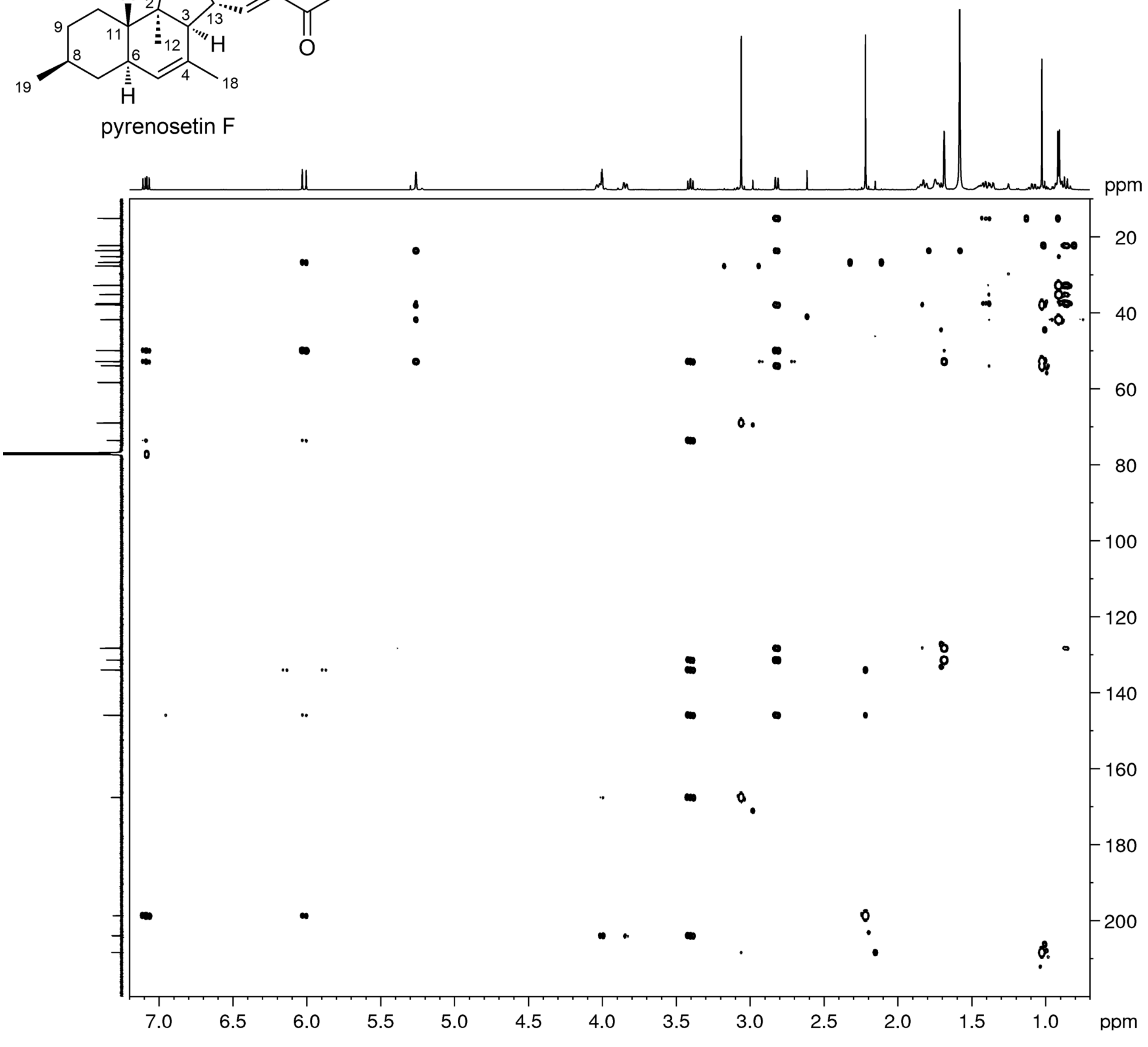

Figure S17. HMBC spectrum of pyrenosetin F (**6**) (600 MHz, $CDCl_3$)

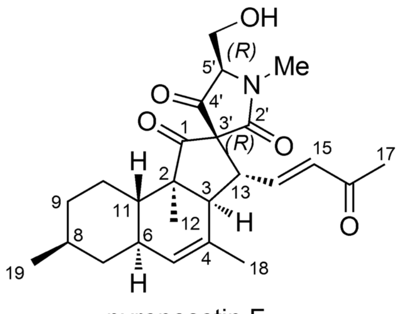

pyrenosetin F

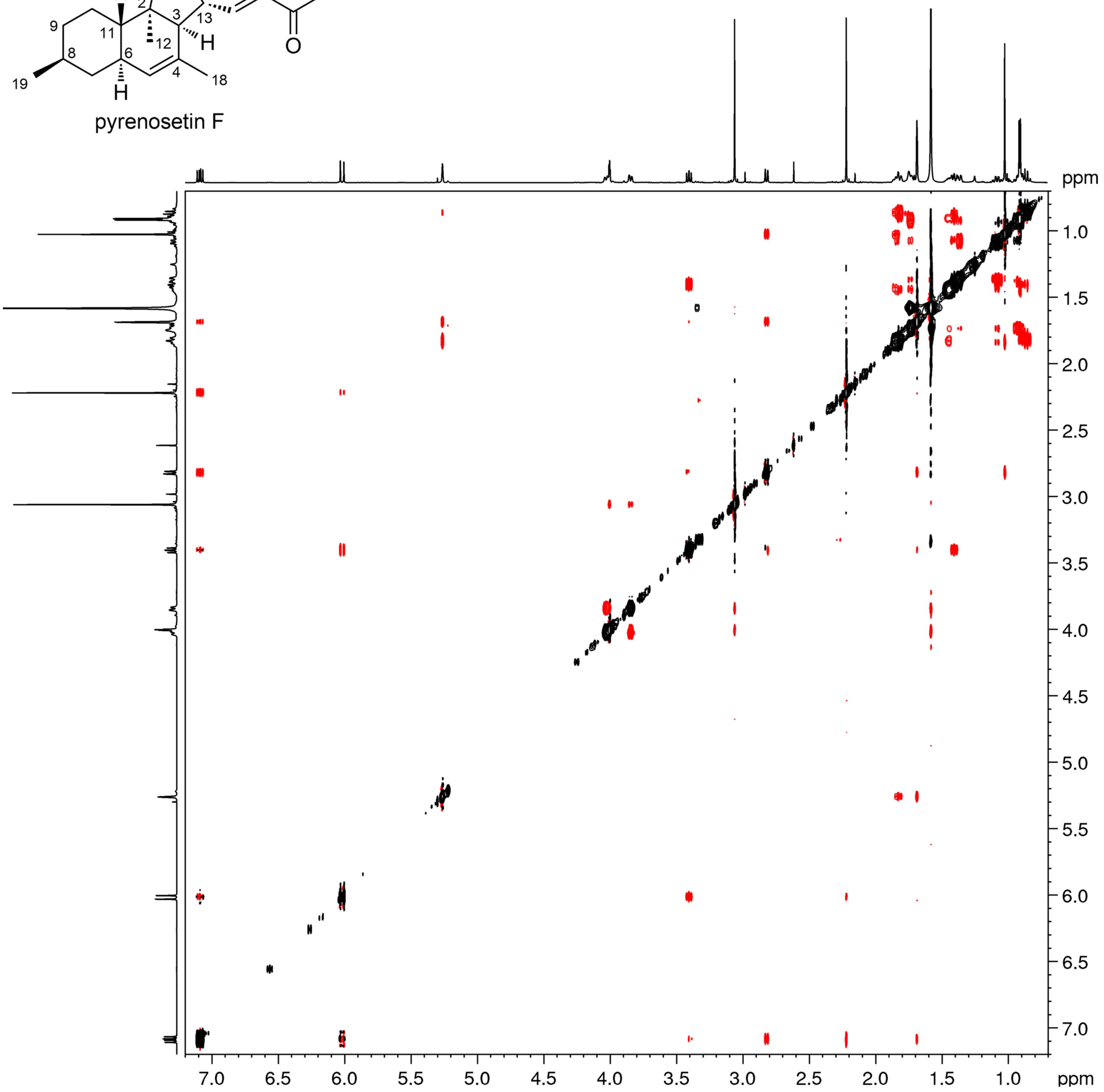

Figure S18. NOESY spectrum of pyrenosetin F (**6**) (600 MHz, $CDCl_3$)

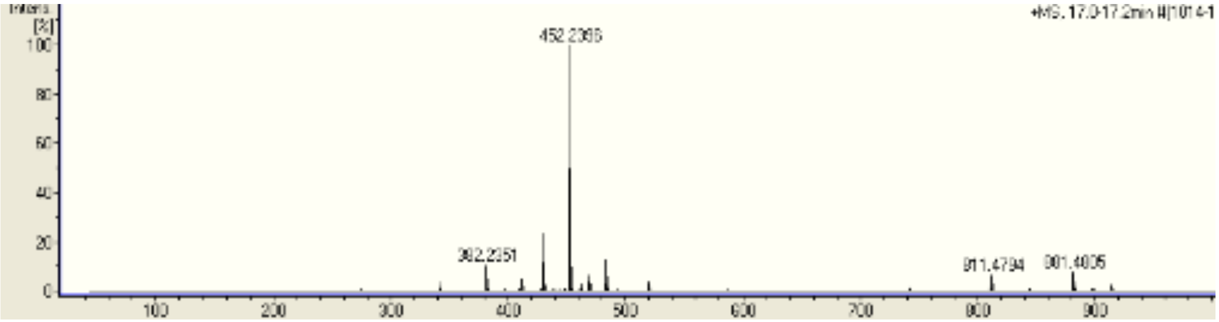

Figure S19. High-resolution ESI mass spectrum of pyrenosetin F (**6**) (positive ion mode).

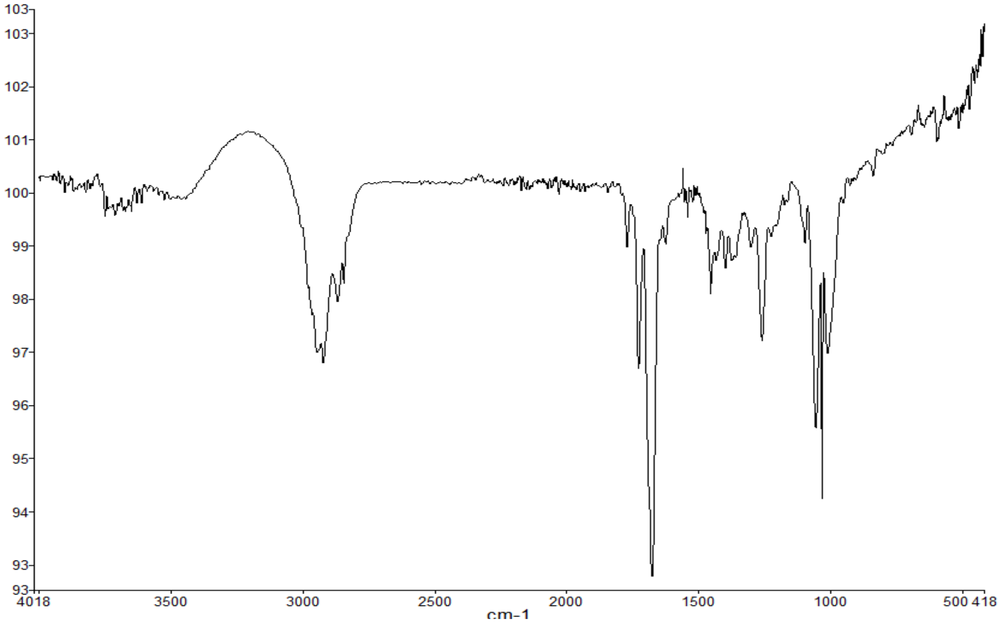

Figure S20. FT-IR spectrum of pyrenosetin F (**6**)

Functional		Solvent?		Basis Set?		Type of Data	
mPW1PW91		PCM		6-311+G(d,p)		Shielding Tensors	
	3'R,5'R	3'S,5'R	3'R,5'S	3'S,5'S	Isomer 5	Isomer 6	
sDP4+ (H data)	1.94%	30.30%	2.27%	65.49%	-	-	
sDP4+ (C data)	21.68%	0.13%	0.03%	78.16%	-	-	
sDP4+ (all data)	0.82%	0.07%	0.00%	99.11%	-	-	
uDP4+ (H data)	12.73%	11.65%	1.57%	74.05%	-	-	
uDP4+ (C data)	8.88%	0.00%	0.00%	91.12%	-	-	
uDP4+ (all data)	1.65%	0.00%	0.00%	98.35%	-	-	
DP4+ (H data)	0.47%	6.75%	0.07%	92.71%	-	-	
DP4+ (C data)	2.63%	0.00%	0.00%	97.37%	-	-	
DP4+ (all data)	0.01%	0.00%	0.00%	99.99%	-	-	

Figure S21. DP4+ results for pyrenosetin E (**5**).

Functional		Solvent?		Basis Set?		Type of Data	
mPW1PW91		PCM		6-311+G(d,p)		Shielding Tensors	
	3'R,5'R	3'S,5'R	3'R,5'S	3'S,5'S	Isomer 5	Isomer 6	
sDP4+ (H data)	13.54%	24.05%	43.28%	19.13%	-	-	
sDP4+ (C data)	84.81%	0.28%	0.23%	14.67%	-	-	
sDP4+ (all data)	79.42%	0.47%	0.70%	19.40%	-	-	
uDP4+ (H data)	92.28%	0.77%	1.54%	5.42%	-	-	
uDP4+ (C data)	15.20%	0.04%	0.00%	84.76%	-	-	
uDP4+ (all data)	75.34%	0.00%	0.00%	24.66%	-	-	
DP4+ (H data)	86.88%	1.28%	4.64%	7.20%	-	-	
DP4+ (C data)	50.90%	0.00%	0.00%	49.10%	-	-	
DP4+ (all data)	92.60%	0.00%	0.00%	7.40%	-	-	

Figure S22. DP4+ results for pyrenosetin F (**6**).

Functional		Solvent?		Basis Set?		Type of Data	
mPW1PW91		PCM		6-311+G(d,p)		Shielding Tensors	
	3'R,5'R	3'S,5'R	3'R,5'S	3'S,5'S	Isomer 5	Isomer 6	
sDP4+ (H data)	0.01%	99.32%	0.21%	0.46%	-	-	
sDP4+ (C data)	2.69%	71.00%	13.43%	12.87%	-	-	
sDP4+ (all data)	0.00%	99.88%	0.04%	0.08%	-	-	
uDP4+ (H data)	0.69%	93.80%	4.48%	1.03%	-	-	
uDP4+ (C data)	1.35%	1.62%	0.02%	97.02%	-	-	
uDP4+ (all data)	0.37%	60.24%	0.03%	39.37%	-	-	
DP4+ (H data)	0.00%	99.98%	0.01%	0.01%	-	-	
DP4+ (C data)	0.26%	8.43%	0.01%	91.29%	-	-	
DP4+ (all data)	0.00%	99.94%	0.00%	0.06%	-	-	

Figure S23. DP4+ results for pyrenosetin C (**3**).

Table S1. Putatively identified compounds by MN-based dereplication of KC subextracts of *Pyrenochaetopsis* sp. strains FVE-001 and FVE-087. Annotation considered the putative ID, retention time (t_R), *m/z* value, adduct, chemical structure, molecular formula, and MS/MS fragments.

Putative ID	t_R (min)	Precursor *m/z* (adduct)	Chemical structure	Molecular formula (Δ ppm)	MS/MS fragmentation
Stereoisomer of phomasetin	10.34	414.2643 [M+H]+	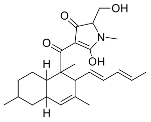	$C_{25}H_{36}NO_4$ (0.1)	396.2635; 346.1981; 328.1932; 215.1779 170.0448
Phomasetin?	10.10	414.3252 [M+H]+	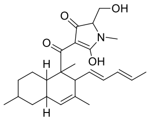	$C_{25}H_{36}NO_4$ (60.8)	396.2533; 346.2022; 243.2124; 215.1813; 170.0458
Phomasetin?	10.10	414.2649 [M+H]+	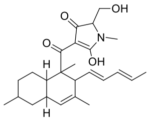	$C_{25}H_{36}NO_4$ (0.5)	396.2543; 346.2011; 215.1797; 170.0451
Phomasetin	10.10	414.2631 [M+H]+	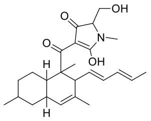	$C_{25}H_{36}NO_4$ (-13)	396.2543; 346.2026; 215.1808; 170.0460
Pyrenosetin A	7.55	412.2484 [M-H2O+H]+	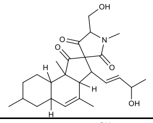	$C_{25}H_{34}NO_4$ (-0.4)	394.2369 384.2527; 210.1128
Stereoisomer of pyrenosetin A/B	7.52	412.2484 [M-H2O+H]+	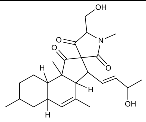	$C_{25}H_{34}NO_4$ (-0.4)	394.2378; 384.2539; 210.1138; 175.1488
Stereoisomer of pyrenosetin A/B	7.98	412.2484 [M-H2O+H]+	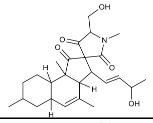	$C_{25}H_{34}NO_4$ (-0.4)	394.2367; 384.2543; 210.1136
Stereoisomer of pyrenosetin A/B	7.28	412.2484 [M-H2O+H]+	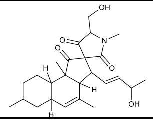	$C_{25}H_{34}NO_4$ (-0.4)	394.2382; 384.2534; 210.1134
Stereoisomer of pyrenosetin A/B	7.50	412.2479 [M-H2O+H]+	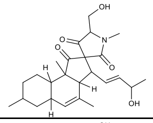	$C_{25}H_{34}NO_4$ (-0.9)	394.2382; 384.2534; 210.1131
Stereoisomer of pyrenosetin A/B	7.00	412.2483 [M-H2O+H]+	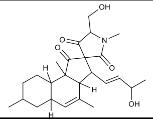	$C_{25}H_{34}NO_4$ (-0.5)	394.2365; 384.2543; 210.1124
Stereoisomer of pyrenosetin A/B	8.43	412.2490 [M-H2O+H]+	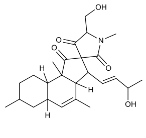	$C_{25}H_{34}NO_4$ (0.2)	394.2372; 384.2547; 210.1143; 175.1490
Stereoisomer of pyrenosetin A/B	7.79	412.2482 [M-H2O+H]+	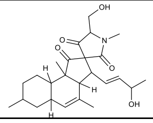	$C_{25}H_{34}NO_4$ (-0.6)	394.2373; 384.2538; 210.1138;

Pyrenosetin B	7.78	412.2488<br>[M-H_2O+H]$^+$	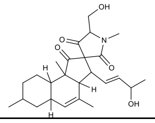	$C_{25}H_{34}NO_4$<br>(0)	394.2374;<br>384.2545;<br>210.1146
Stereoisomer of pyrenosetin A/B	8.28	412.2482<br>[M-H_2O+H]$^+$	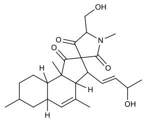	$C_{25}H_{34}NO_4$<br>(-0.6)	394.2284;<br>384.2538;<br>210.1132<br>175.1473
Stereoisomer of pyrenosetin A/B	9.15	412.2487<br>[M-H_2O+H]$^+$	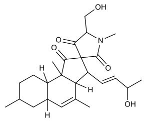	$C_{25}H_{34}NO_4$<br>(-0.1)	394.2350;<br>384.2532;<br>243.2121;<br>170.0458
Stereoisomer of pyrenosetin C	7.98	428.2425<br>[M+H]$^+$	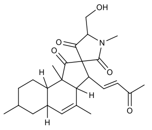	$C_{25}H_{34}NO_5$<br>(-1.2)	410.2326;<br>400.2502;<br>368.2244<br>342.2072<br>226.1078;<br>144.0652
Stereoisomer of pyrenosetin C	7.07	428.2436<br>[M+H]$^+$	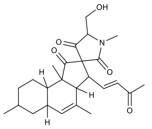	$C_{25}H_{34}NO_5$<br>(-0.1)	410.2374;<br>400.2409;<br>386.2320;<br>368.2212;<br>342.2043;<br>226.1096
Pyrenosetin C	7.87	428.2424<br>[M+H]$^+$	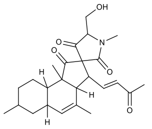	$C_{25}H_{34}NO_5$<br>(-1.3)	410.2322;<br>400.2450;<br>386.2346;<br>368.2229;<br>342.2070;<br>226.1101;<br>144.0666
Pyrenosetin D	7.01	446.2540<br>[M+H]$^+$	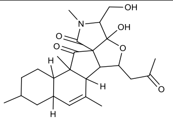	$C_{25}H_{36}NO_6$<br>(-0.3)	428.2442;<br>400.2463<br>386.2330;<br>368.2232;<br>342.2081<br>226.1077
Wakodecaline A	6.41	430.2582<br>[M-H_2O+H]$^+$	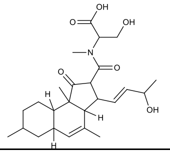	$C_{25}H_{36}NO_5$<br>(-1.3)	412.2473;<br>311.2007;<br>265.1988;<br>120.0669
Stereoisomer of wakodecaline A	6.90	430.2586<br>[M-H_2O+H]$^+$	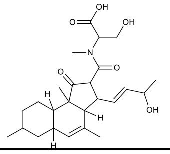	$C_{25}H_{36}NO_5$<br>(-0.4)	412.2491;<br>311.2001;<br>283.2072;<br>120.0654
Stereoisomer of wakodecaline A	6.57	430.2586<br>[M-H_2O+H]$^+$	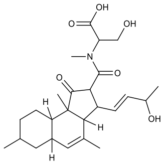	$C_{25}H_{36}NO_5$<br>(-0.4)	412.2470;<br>311.2004;<br>283.2058;<br>265.1949;<br>120.0665

Wakodecaline B	6.81	446.2538 [M+H]+	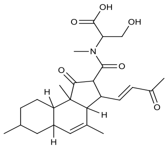	$C_{25}H_{36}NO_6$ (-0.5)	428.2430; 327.1975; 299.2016; 281.1905; 120.0666
Stereoisomer of wakodecaline B	6.93	4466.2539 [M+H]+	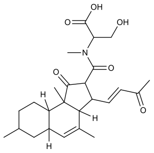	$C_{25}H_{36}NO_6$ (-0.4)	428.2425; 327.1961; 299.2015; 281.1916; 120.0666

Table S2. Experimental ^{13}C and ^{1}H NMR chemical shifts of pyrenosetins E (**5**) and Boltzmann-averaged ^{13}C and ^{1}H NMR isotropic shielding values of its four possible stereoisomers at C-3' and C-5' calculated at the mPW1PW91/6-311+G(d,p)/PCM($CHCl_3$) level of theory.

	Experimental chemical shifts	Predicted isotropic shielding values			
	pyrenosetin E	3'R,5'R	3'S,5'R	3'R,5'S	3'S,5'S
C-1	209.6	-35.4	-39.8	-41.1	-34.8
C-2	54.3	126.8	125.9	126.5	126.2
C-3	53.4	131.2	130.1	131.2	129.9
C-4	132.1	44.7	45.2	45.1	44.9
C-5	127.8	51.8	51.5	51.5	51.8
C-6	37.6	145.4	145.4	145.3	145.7
C-7	42.0	143.5	143.6	143.5	143.6
C-8	33.0	151.08	151.06	151.23	151.01
C-9	35.4	150.20	150.35	150.22	150.29
C-10	25.3	159.68	159.57	159.72	159.81
C-11	37.8	146.20	147.18	145.92	147.03
C-12	15.4	170.91	171.20	171.09	171.00
C-13	49.4	129.91	129.99	129.42	132.42
C-14	131.2	47.93	50.69	49.96	45.76
C-15	136.8	41.31	40.63	40.63	42.92
C-16	69.0	113.85	114.21	114.00	113.65
C-17	22.8	164.54	163.64	164.60	164.50
C-18	23.9	161.27	161.09	161.01	161.35
C-19	22.4	164.68	164.78	164.79	164.66
C-2'	169.1	12.59	12.55	13.33	11.83
C-3’	74.4	106.55	107.17	107.46	105.62
C-4'	204.8	-33.10	-36.12	-35.34	-32.83
C-5’	68.8	113.91	114.00	113.08	114.59
C-6’	57.9	123.91	123.31	122.85	125.38
C-7’	28.0	159.19	159.10	159.36	159.11
H-3	2.57	28.86	29.12	28.80	29.16
H-5	5.22	26.14	26.11	26.12	26.14
H-6	1.82	29.84	29.88	29.85	29.86
H-7a	1.79	30.02	30.02	30.00	30.03
H-7b	0.85	30.94	30.91	30.95	30.89
H-8	1.42	30.30	30.27	30.28	30.30
H-9a	1.72	30.13	30.15	30.12	30.14
H-9b	0.97	30.83	30.76	30.83	30.76
H-10a	1.43	30.40	30.45	30.38	30.44
H-10b	1.05	30.71	30.75	30.72	30.73
H-11	1.56	30.40	30.14	30.41	30.13
H-12	0.98	30.82	30.85	30.82	30.85
H-13	3.42	28.40	28.26	28.43	28.21
H-14	5.82	25.43	25.67	25.47	25.53
H-15	5.55	25.99	25.85	25.93	25.98
H-16	4.17	27.60	27.57	27.55	27.64
H-17	1.18	30.64	30.63	30.62	30.63
H-18	1.70	29.99	29.99	29.99	29.99
H-19	0.89	30.86	30.85	30.86	30.86
H-5’	3.85	27.86	28.25	28.20	27.98
H-6’a	4.10	27.84	27.70	27.61	27.62
H-6'b	3.87	27.88	27.85	27.83	27.96
H-7’	3.09	28.72	28.70	28.68	28.69

Table S3. Experimental ^{13}C and ^{1}H NMR chemical shifts of pyrenosetins F (**6**) and C (**3**) and Boltzmann-averaged ^{13}C and ^{1}H NMR isotropic shielding values of their four possible stereoisomers at C-3' and C-5' calculated at the mPW1PW91/6-311+G(d,p)/PCM($CHCl_3$) level of theory.

	Experimental chemical shifts		Predicted isotropic shielding values			
	pyrenosetin F (**6**)	pyrenosetin C (**3**)	3'R,5'R	3'S,5'R	3'R,5'S	3'S,5'S
C-1	208.5	212.1	-33.7	-36.8	-38.3	-32.8
C-2	54.1	54.7	127.0	126.5	126.6	127.0
C-3	52.9	53.6	130.9	130.3	131.0	130.5
C-4	131.5	130.9	45.5	45.9	45.8	45.8
C-5	128.4	128.8	50.9	50.5	50.6	50.6
C-6	37.7	37.6	145.3	145.4	145.3	145.3
C-7	41.9	41.8	143.6	143.7	143.6	143.6
C-8	32.9	32.9	151.10	151.01	151.14	151.01
C-9	35.3	35.2	150.21	150.32	150.25	150.28
C-10	25.3	25.2	159.75	159.76	159.81	159.83
C-11	38.0	37.4	146.03	146.62	146.07	146.23
C-12	15.3	15.2	170.69	170.83	170.97	170.52
C-13	50.0	50.6	131.64	131.14	130.08	132.09
C-14	146.1	144.4	29.39	31.43	30.22	30.69
C-15	134.1	133.9	48.38	49.83	48.74	49.90
C-16	198.8	197.6	-19.67	-18.92	-19.57	-19.10
C-17	26.8	27.6	158.04	156.62	157.83	156.69
C-18	23.8	23.7	161.48	161.52	161.50	161.55
C-19	22.4	22.4	164.71	164.75	164.73	164.70
C-2'	167.7	167.8	13.15	13.13	13.74	12.93
C-3’	73.7	72.7	106.62	107.09	107.71	106.02
C-4'	204.1	206.4	-30.69	-31.12	-32.47	-29.64
C-5’	69.1	69.8	113.45	112.50	112.30	114.68
C-6’	58.4	60.3	125.43	120.97	122.07	122.89
C-7’	27.8	28.5	159.43	157.50	158.40	158.10
H-3	2.82	2.66	28.81	28.96	28.72	28.94
H-5	5.26	5.28	26.08	26.09	26.08	26.08
H-6	1.85	1.83	29.83	29.84	29.81	29.83
H-7a	1.82	1.82	30.01	30.01	30.00	30.01
H-7b	0.86	0.88	30.92	30.91	30.93	30.91
H-8	1.45	1.44	30.28	30.28	30.28	30.29
H-9a	1.74	1.73	30.13	30.12	30.11	30.12
H-9b	0.92	0.99	30.85	30.77	30.83	30.78
H-10a	1.37	1.41	30.44	30.39	30.41	30.36
H-10b	1.00	1.04	30.68	30.72	30.69	30.69
H-11	1.41	1.64	30.44	30.24	30.39	30.31
H-12	1.02	1.01	30.77	30.82	30.78	30.80
H-13	3.40	3.57	28.26	28.20	28.32	28.20
H-14	7.09	6.85	24.11	24.34	24.17	24.28
H-15	6.02	6.18	25.53	25.35	25.52	25.36
H-17	2.22	2.22	29.52	29.54	29.51	29.54
H-18	1.69	1.68	30.03	30.05	30.04	30.05
H-19	0.91	0.90	30.86	30.85	30.85	30.85
H-5’	4.00	3.61	27.80	28.13	28.12	27.74
H-6’a	4.03	4.10	27.71	27.59	27.59	27.96
H-6'b	3.84	3.94	27.92	27.85	27.84	27.85
H-7’	3.06	3.11	28.72	28.66	28.66	28.67

Table S4. Theoretical relative Gibbs free energies (kcal/mol) calculated at the B3LYP/TZVP/SMD($CHCl_3$)//B3LYP/6-31+G(d,p)/SMD($CHCl_3$) level, and populations according to the Boltzmann distribution (300 K) of populated (>1%) conformers of the four stereoisomers of pyrenosetins E (**5**) considered in this study.

	3'*R*,5'*R*		3'*S*,5'*R*		3'*R*,5'*S*		3'*S*,5'*S* (**5**)	
	Δ*G*	% pop.	ΔG	% pop.	ΔG	% pop.	ΔG	% pop.
1	0.00	17.1%	0.00	16.1%	0.00	37.5%	0.00	20.7%
2	0.04	16.1%	0.06	14.5%	0.75	10.5%	0.10	17.4%
3	0.14	13.4%	0.21	11.2%	0.78	10.1%	0.13	16.5%
4	0.27	10.9%	0.36	8.7%	0.88	8.4%	0.23	14.1%
5	0.57	6.5%	0.39	8.3%	1.06	6.3%	0.28	13.0%
6	0.59	6.3%	0.39	8.3%	1.12	5.7%	0.68	6.6%
7	0.72	5.1%	0.44	7.6%	1.20	4.9%	0.82	5.1%
8	0.73	5.0%	0.79	4.2%	1.29	4.2%	1.38	2.0%
9	0.81	4.4%	0.91	3.5%	1.42	3.4%	1.51	1.6%
10	0.82	4.3%	0.91	3.5%	1.44	3.3%	1.77	1.0%
11	1.00	3.2%	0.95	3.2%	1.76	1.9%		
12	1.07	2.8%	1.08	2.6%	1.79	1.8%		
13	1.10	2.7%	1.19	2.2%	2.07	1.1%		
14	1.24	2.1%	1.24	2.0%				
15			1.62	1.0%				
16			1.63	1.0%				
17			1.65	1.0%				

Table S5. Theoretical relative Gibbs free energies (kcal/mol) calculated at the B3LYP/TZVP/SMD($CHCl_3$)//B3LYP/6-31+G(d,p)/SMD($CHCl_3$) level, and populations according to the Boltzmann distribution (300 K) of populated (>1%) conformers of the four stereoisomers of pyrenosetins F (**6**) considered in this study.

	3'*R*,5'*R* (**6**)		3'*S*,5'*R* (**3**)		3'*R*,5'*S*		3'*S*,5'*S* (**5**)	
	Δ*G*	% pop.	ΔG	% pop.	ΔG	% pop.	ΔG	% pop.
1	0.00	28.1%	0.00	20.1%	0.00	27.7%	0.00	14.9%
2	0.16	21.3%	0.09	17.1%	0.33	15.9%	0.05	13.7%
3	0.60	10.2%	0.26	12.9%	0.68	8.7%	0.06	13.5%
4	0.70	8.5%	0.32	11.6%	0.69	8.7%	0.06	13.4%
5	0.72	8.4%	0.37	10.8%	0.72	8.2%	0.09	12.7%
6	0.83	6.9%	0.50	8.6%	0.83	6.8%	0.50	6.4%
7	0.92	5.9%	0.69	6.3%	0.91	5.9%	0.55	5.9%
8	1.08	4.6%	0.72	6.0%	0.91	5.9%	0.61	5.3%
9	1.22	3.6%	0.81	5.1%	1.07	4.5%	0.64	5.0%
10	1.40	2.6%	0.82	5.1%	1.12	4.2%	0.70	4.6%
11			0.95	4.1%	1.24	3.4%	0.70	4.5%
12			0.97	3.9%			0.73	4.4%
13							0.77	4.0%
14							1.63	1.0%

Table S6. Cartesian coordinates of the six lowest-energy conformers of the 3'*R*,5'*R* stereoisomer of pyrenosetins E at the B3LYP/TZVP/SMD($CHCl_3$)//B3LYP/6-31+G(d,p)/SMD($CHCl_3$) level.

Conformer 1				Conformer 2				Conformer 3			
C	-0.3990	-1.4319	0.9830	C	-0.5355	-1.5204	0.8922	C	-0.5353	-1.5979	0.7804
C	-1.4794	-0.4124	1.3263	C	-1.5391	-0.4461	1.3023	C	-1.5353	-0.5471	1.2575
C	-0.7108	0.9401	1.3131	C	-0.6724	0.8438	1.3732	C	-0.6647	0.7348	1.4076
C	-1.6017	2.1548	1.1349	C	-1.4632	2.1327	1.2569	C	-1.4522	2.0301	1.3737
C	-2.8655	2.0353	0.7026	C	-2.7310	2.1367	0.8204	C	-2.7210	2.0647	0.9415
C	-3.5422	0.7385	0.3487	C	-3.5059	0.9168	0.3995	C	-3.5004	0.8759	0.4472
C	-4.4352	0.8555	-0.9042	C	-4.3868	1.1731	-0.8411	C	-4.3829	1.2121	-0.7733
C	-5.1963	-0.4506	-1.1946	C	-5.2450	-0.0518	-1.2045	C	-5.2466	0.0154	-1.2104
C	-4.2182	-1.6366	-1.2765	C	-4.3597	-1.3019	-1.3586	C	-4.3669	-1.2264	-1.4428
C	-3.3016	-1.7382	-0.0458	C	-3.4547	-1.5439	-0.1392	C	-3.4607	-1.5469	-0.2426
C	-2.5400	-0.4187	0.1675	C	-2.5945	-0.3013	0.1484	C	-2.5945	-0.3278	0.1187
C	-2.0835	-0.7401	2.7003	C	-2.1694	-0.8136	2.6543	C	-2.1629	-0.9972	2.5860
C	0.4095	0.7731	0.2274	C	0.4406	0.6615	0.2871	C	0.4428	0.6116	0.3079
C	1.5641	1.7357	0.3211	C	1.6802	1.4999	0.4477	C	1.6812	1.4479	0.4828
C	2.0768	2.4064	-0.7166	C	2.3222	2.0969	-0.5613	C	2.2651	2.1411	-0.5001
C	3.2654	3.3293	-0.6295	C	3.6690	2.7569	-0.4241	C	3.6046	2.8208	-0.3805
C	2.9682	4.7202	-1.1797	C	3.7187	4.1573	-1.0241	C	3.5700	4.2853	-0.8027
C	-1.0251	3.4931	1.5241	C	-0.7755	3.4000	1.6995	C	-0.7620	3.2640	1.8989
C	-6.0551	-0.3360	-2.4582	C	-6.0912	0.2015	-2.4563	C	-6.0932	0.3491	-2.4430
C	2.0237	-1.0098	1.2943	C	1.9190	-1.2834	1.2397	C	1.9018	-1.3558	1.2275
C	0.8388	-0.7141	0.3632	C	0.7564	-0.8635	0.3267	C	0.7700	-0.9103	0.2870
C	1.2421	-1.3928	-0.9343	C	1.1350	-1.4684	-1.0162	C	1.2178	-1.4409	-1.0638
C	2.4688	-2.2679	-0.6796	C	2.3560	-2.3755	-0.8334	C	2.4457	-2.3332	-0.8775
C	3.5360	-2.0554	-1.7673	C	3.4371	-2.0974	-1.8753	C	3.5646	-1.9785	-1.8694
C	3.9849	-2.5008	1.3676	C	3.8953	-2.7582	1.1583	C	3.8973	-2.8013	1.1637
H	-0.1930	1.0334	2.2778	H	-0.1595	0.8424	2.3450	H	-0.1487	0.6716	2.3752
H	-3.4812	2.9325	0.6290	H	-3.2720	3.0833	0.7888	H	-3.2600	3.0124	0.9727
H	-4.2153	0.4769	1.1830	H	-4.1970	0.6602	1.2206	H	-4.1907	0.5711	1.2523
H	-5.1479	1.6800	-0.7703	H	-5.0346	2.0405	-0.6572	H	-5.0268	2.0688	-0.5343
H	-3.8110	1.1188	-1.7707	H	-3.7436	1.4381	-1.6931	H	-3.7402	1.5272	-1.6085
H	-5.8686	-0.6343	-0.3420	H	-5.9303	-0.2333	-0.3617	H	-5.9315	-0.2149	-0.3795
H	-4.7789	-2.5718	-1.4000	H	-4.9896	-2.1831	-1.5343	H	-5.0008	-2.0925	-1.6714
H	-3.5984	-1.5251	-2.1784	H	-3.7323	-1.1854	-2.2546	H	-3.7407	-1.0575	-2.3314
H	-2.6046	-2.5727	-0.1712	H	-2.8230	-2.4202	-0.3151	H	-2.8334	-2.4136	-0.4731
H	-3.9077	-1.9665	0.8404	H	-4.0773	-1.7766	0.7343	H	-4.0826	-1.8297	0.6166
H	-1.9905	-0.2176	-0.7636	H	-2.0290	-0.0888	-0.7705	H	-2.0318	-0.0607	-0.7878
H	-0.0449	0.8696	-0.7645	H	0.0070	0.8571	-0.6997	H	-0.0013	0.8425	-0.6666
H	2.0144	1.8511	1.3065	H	2.1110	1.5342	1.4476	H	2.1606	1.4012	1.4601
H	1.6464	2.2876	-1.7124	H	1.9203	2.0459	-1.5744	H	1.8040	2.1835	-1.4889
H	3.5739	3.4163	0.4240	H	3.9258	2.8153	0.6459	H	3.9429	2.7561	0.6659
H	2.1526	-3.3235	-0.6952	H	2.0367	-3.4226	-0.9544	H	2.1563	-3.3810	-1.0577
H	3.0426	-2.1632	-2.7420	H	4.2755	-2.7946	-1.7404	H	4.4611	-2.5575	-1.6303
H	3.9259	-1.0350	-1.6966	H	2.9930	-2.2887	-2.8592	H	3.2331	-2.2761	-2.8706
H	4.3554	-3.8208	-1.8552	H	4.2420	-0.4671	-2.6021	H	3.3161	-0.1095	-2.3952
H	-2.8388	-0.0015	2.9817	H	-2.8667	-0.0390	2.9846	H	-2.8582	-0.2435	2.9652
H	-2.5447	-1.7305	2.7068	H	-2.7048	-1.7644	2.5992	H	-2.7004	-1.9416	2.4725
H	-1.3041	-0.7314	3.4690	H	-1.3929	-0.9130	3.4197	H	-1.3847	-1.1451	3.3415
H	2.6412	4.6581	-2.2232	H	3.4351	4.1329	-2.0819	H	3.2122	4.3798	-1.8338
H	3.8690	5.3407	-1.1388	H	4.7328	4.5635	-0.9508	H	4.5746	4.7168	-0.7482
H	2.1815	5.2089	-0.5972	H	3.0362	4.8300	-0.4953	H	2.9073	4.8617	-0.1498
H	-1.7648	4.2918	1.4135	H	-1.4462	4.2617	1.6287	H	-1.4293	4.1309	1.8804
H	-0.6855	3.4858	2.5684	H	-0.4325	3.3196	2.7396	H	-0.4245	3.1156	2.9334
H	-0.1518	3.7522	0.9156	H	0.1140	3.6114	1.0951	H	0.1313	3.5101	1.3137
H	-5.4331	-0.1504	-3.3428	H	-5.4560	0.3910	-3.3305	H	-5.4583	0.5891	-3.3050
H	-6.7743	0.4875	-2.3801	H	-6.7459	1.0713	-2.3276	H	-6.7437	1.2122	-2.2600
H	-6.6219	-1.2566	-2.6395	H	-6.7262	-0.6612	-2.6894	H	-6.7324	-0.4948	-2.7276
H	3.8630	-3.5897	1.3753	H	3.8339	-3.8448	1.0331	H	3.8554	-3.8779	0.9649
H	4.9353	-2.2618	0.8863	H	4.8302	-2.4004	0.7146	H	4.8532	-2.4098	0.8010
H	3.9861	-2.1298	2.3927	H	3.8913	-2.5166	2.2213	H	3.8288	-2.6289	2.2380
H	4.5753	1.9423	-1.0556	H	4.4931	1.0437	-0.8908	H	4.5314	1.2189	-1.0413
N	2.8696	-1.8769	0.6697	N	2.7471	-2.1132	0.5465	N	2.7778	-2.1282	0.5280
O	-0.4430	-2.6298	1.1691	O	-0.6743	-2.7212	0.9985	O	-0.6841	-2.8017	0.7942
O	4.3536	2.8131	-1.4169	O	4.6548	1.9739	-1.1196	O	4.5493	2.1689	-1.2449
O	0.7043	-1.2533	-2.0124	O	0.5786	-1.2690	-2.0736	O	0.7261	-1.1679	-2.1388
O	2.1549	-0.5656	2.4309	O	2.0531	-0.9480	2.4126	O	1.9776	-1.0798	2.4210
O	4.6505	-2.9237	-1.6389	O	3.8650	-0.7434	-1.7548	O	3.9327	-0.6035	-1.8358

Table S6 (continued)

Conformer 4				Conformer 5				Conformer 6			
C	0.4042	-1.3938	-1.0796	C	0.4651	-1.4788	-0.9711	C	0.5702	-1.3986	-1.0804
C	1.4417	-0.3170	-1.3759	C	1.4972	-0.4121	-1.3284	C	1.5713	-0.2842	-1.3716
C	0.6163	1.0006	-1.3205	C	0.6621	0.9012	-1.3573	C	0.6962	0.9993	-1.3190
C	1.4546	2.2444	-1.0942	C	1.4842	2.1641	-1.1871	C	1.4737	2.2760	-1.0629
C	2.7196	2.1623	-0.6569	C	2.7481	2.1197	-0.7414	C	2.7377	2.2452	-0.6168
C	3.4476	0.8834	-0.3423	C	3.4892	0.8665	-0.3600	C	3.5185	0.9938	-0.3158
C	4.3262	0.9938	0.9214	C	4.3658	1.0546	0.8959	C	4.3854	1.1291	0.9536
C	5.1399	-0.2884	1.1726	C	5.1917	-0.2036	1.2181	C	5.2475	-0.1218	1.2014
C	4.2128	-1.5170	1.2043	C	4.2755	-1.4366	1.3184	C	4.3688	-1.3857	1.2207
C	3.3092	-1.6136	-0.0364	C	3.3748	-1.6109	0.0843	C	3.4776	-1.5096	-0.0262
C	2.4939	-0.3205	-0.2092	C	2.5468	-0.3379	-0.1624	C	2.6132	-0.2488	-0.1976
C	2.0689	-0.5693	-2.7555	C	2.1298	-0.7422	-2.6895	C	2.2176	-0.5087	-2.7464
C	-0.5004	0.7475	-0.2482	C	-0.4635	0.6972	-0.2874	C	-0.4250	0.7114	-0.2669
C	-1.6924	1.6659	-0.3068	C	-1.6707	1.5909	-0.3841	C	-1.6912	1.5142	-0.4160
C	-2.2220	2.2848	0.7545	C	-2.2354	2.1966	0.6646	C	-2.4645	1.8836	0.6075
C	-3.4456	3.1635	0.7053	C	-3.5001	3.0084	0.6017	C	-3.8498	2.4438	0.4378
C	-3.1882	4.5586	1.2651	C	-3.2963	4.4416	1.0982	C	-4.1702	3.5581	1.4289
C	0.8250	3.5698	-1.4427	C	0.8367	3.4631	-1.5970	C	0.7713	3.5755	-1.3669
C	5.9838	-0.1825	2.4469	C	6.0340	-0.0185	2.4844	C	6.0796	0.0092	2.4812
C	-2.0310	-1.0595	-1.4037	C	-1.9548	-1.1383	-1.4241	C	-1.9184	-1.1680	-1.2825
C	-0.8697	-0.7480	-0.4449	C	-0.8314	-0.8076	-0.4265	C	-0.7049	-0.8215	-0.4100
C	-1.2636	-1.4941	0.8134	C	-1.3105	-1.4622	0.8550	C	-1.0056	-1.5316	0.9073
C	-2.4111	-2.4448	0.4966	C	-2.5478	-2.3027	0.5684	C	-2.3143	-2.3131	0.7740
C	-3.5071	-2.3610	1.5811	C	-3.6781	-1.9581	1.5668	C	-3.2931	-2.0206	1.9265
C	-3.8988	-2.6650	-1.5625	C	-3.9408	-2.5972	-1.5462	C	-3.9761	-2.5152	-1.1466
H	0.1000	1.1061	-2.2847	H	0.1573	0.9491	-2.3318	H	0.1924	1.0927	-2.2917
H	3.2971	3.0811	-0.5485	H	3.3134	3.0501	-0.6745	H	3.2685	3.1880	-0.4782
H	4.1370	0.6793	-1.1793	H	4.1809	0.6244	-1.1848	H	4.2191	0.8261	-1.1516
H	5.0042	1.8517	0.8220	H	5.0354	1.9124	0.7495	H	5.0296	2.0139	0.8647
H	3.6854	1.2004	1.7912	H	3.7224	1.3025	1.7528	H	3.7319	1.3034	1.8208
H	5.8257	-0.4130	0.3200	H	5.8788	-0.3695	0.3737	H	5.9423	-0.2142	0.3519
H	4.8119	-2.4314	1.2989	H	4.8825	-2.3390	1.4649	H	5.0026	-2.2766	1.3143
H	3.5830	-1.4645	2.1047	H	3.6441	-1.3391	2.2139	H	3.7313	-1.3628	2.1168
H	2.6478	-2.4810	0.0539	H	2.7216	-2.4780	0.2218	H	2.8487	-2.4017	0.0555
H	3.9302	-1.7844	-0.9253	H	3.9991	-1.8248	-0.7928	H	4.1103	-1.6517	-0.9120
H	1.9311	-0.1751	0.7244	H	1.9806	-0.1459	0.7608	H	2.0371	-0.1335	0.7320
H	-0.0535	0.8223	0.7489	H	-0.0253	0.8117	0.7100	H	-0.0189	0.8602	0.7393
H	-2.1544	1.7928	-1.2852	H	-2.1306	1.6796	-1.3677	H	-2.0351	1.6881	-1.4344
H	-1.7777	2.1546	1.7428	H	-1.7937	2.0970	1.6572	H	-2.1569	1.6761	1.6315
H	-3.7817	3.2507	-0.3397	H	-3.8563	3.0352	-0.4399	H	-3.9695	2.8160	-0.5896
H	-2.0304	-3.4769	0.4711	H	-2.3032	-3.3689	0.6830	H	-2.0827	-3.3902	0.8037
H	-3.8428	-1.3162	1.6752	H	-3.8788	-0.8768	1.5321	H	-4.2431	-2.5376	1.7246
H	-4.3692	-2.9634	1.2805	H	-4.5940	-2.4790	1.2727	H	-2.8628	-2.4757	2.8244
H	-2.2500	-2.3744	3.0452	H	-2.5118	-1.9732	3.1065	H	-3.9872	-0.1991	1.5167
H	2.7944	0.2101	-3.0029	H	2.8476	0.0282	-2.9837	H	2.9189	0.2948	-2.9872
H	2.5713	-1.5387	-2.7935	H	2.6427	-1.7066	-2.6670	H	2.7521	-1.4608	-2.7838
H	1.2955	-0.5648	-3.5302	H	1.3576	-0.7936	-3.4636	H	1.4502	-0.5287	-3.5270
H	-2.8317	4.4976	2.2990	H	-2.9244	4.4420	2.1284	H	-4.0618	3.2007	2.4585
H	-4.1135	5.1434	1.2552	H	-4.2425	4.9959	1.0747	H	-5.1957	3.9220	1.2932
H	-2.4372	5.0846	0.6683	H	-2.5791	4.9781	0.4684	H	-3.4939	4.4067	1.2840
H	1.5304	4.3942	-1.3008	H	1.5300	4.3032	-1.4930	H	1.4281	4.4332	-1.1930
H	0.4914	3.5833	-2.4889	H	0.5034	3.4238	-2.6426	H	0.4365	3.6087	-2.4123
H	-0.0614	3.7723	-0.8316	H	-0.0530	3.6811	-0.9955	H	-0.1255	3.7056	-0.7494
H	5.3481	-0.0557	3.3320	H	5.3967	0.1529	3.3609	H	5.4343	0.1046	3.3633
H	6.6673	0.6735	2.4044	H	6.7102	0.8394	2.3936	H	6.7288	0.8920	2.4483
H	6.5885	-1.0837	2.6003	H	6.6462	-0.9045	2.6888	H	6.7188	-0.8683	2.6323
H	-3.7292	-3.7470	-1.5858	H	-3.8962	-3.6860	-1.4362	H	-3.9535	-3.6077	-1.0644
H	-4.8745	-2.4688	-1.1071	H	-4.9111	-2.2433	-1.1837	H	-4.8789	-2.1410	-0.6535
H	-3.8990	-2.2770	-2.5813	H	-3.8400	-2.3349	-2.5996	H	-4.0052	-2.2322	-2.1991
H	-4.6796	1.7159	1.1574	H	-5.2541	2.8590	1.4624	H	-5.6319	1.6600	0.8194
N	-2.8292	-2.0051	-0.8304	N	-2.8439	-1.9763	-0.8213	N	-2.7788	-1.9384	-0.5595
O	0.4975	-2.5803	-1.3100	O	0.5831	-2.6783	-1.1034	O	0.7053	-2.5764	-1.3394
O	-4.4945	2.5980	1.5116	O	-4.4525	2.3157	1.4281	O	-4.7472	1.3160	0.6238
O	-0.7869	-1.3328	1.9221	O	-0.8316	-1.2978	1.9622	O	-0.3226	-1.4905	1.9070
O	-2.1809	-0.5668	-2.5163	O	-2.0108	-0.7397	-2.5831	O	-2.0751	-0.8100	-2.4459
O	-3.0394	-2.8887	2.8065	O	-3.3479	-2.4063	2.8674	O	-3.4755	-0.6531	2.2132

Table S7. Cartesian coordinates of the six lowest-energy conformers of the 3'*S*,5'*R* stereoisomer of pyrenosetins E at the B3LYP/TZVP/SMD($CHCl_3$)//B3LYP/6-31+G(d,p)/SMD($CHCl_3$) level.

Conformer 1				Conformer 2				Conformer 3			
C	0.2457	-1.4916	-0.7207	C	0.2667	-1.3352	-0.9379	C	0.2797	-1.3251	-0.9484
C	1.2931	-0.5348	-1.2756	C	1.2761	-0.2795	-1.3511	C	1.2865	-0.2639	-1.3546
C	0.5241	0.8135	-1.3770	C	0.4260	1.0240	-1.3638	C	0.4316	1.0368	-1.3614
C	1.4239	2.0341	-1.4305	C	1.2501	2.2943	-1.2673	C	1.2501	2.3095	-1.2529
C	2.7149	1.9626	-1.0750	C	2.5265	2.2659	-0.8570	C	2.5261	2.2837	-0.8414
C	3.4178	0.7185	-0.6035	C	3.2842	1.0269	-0.4615	C	3.2882	1.0447	-0.4541
C	4.3963	0.9884	0.5587	C	4.1887	1.2455	0.7694	C	4.1907	1.2589	0.7790
C	5.1830	-0.2744	0.9539	C	5.0325	-0.0011	1.0919	C	5.0376	0.0130	1.0959
C	4.2201	-1.4387	1.2502	C	4.1306	-1.2407	1.2356	C	4.1388	-1.2297	1.2323
C	3.2177	-1.6884	0.1109	C	3.1993	-1.4436	0.0286	C	3.2099	-1.4289	0.0229
C	2.4366	-0.4015	-0.2058	C	2.3575	-0.1794	-0.2138	C	2.3649	-0.1659	-0.2146
C	1.7964	-1.0385	-2.6363	C	1.8729	-0.6193	-2.7242	C	1.8869	-0.5939	-2.7284
C	-0.4987	0.8019	-0.1881	C	-0.6361	0.8424	-0.2243	C	-0.6337	0.8460	-0.2273
C	-1.6595	1.7539	-0.3107	C	-1.8629	1.7093	-0.3314	C	-1.8716	1.6969	-0.3397
C	-2.0685	2.5762	0.6621	C	-2.3786	2.4149	0.6815	C	-2.4273	2.3520	0.6837
C	-3.2536	3.5009	0.5554	C	-3.6303	3.2489	0.5891	C	-3.6836	3.1748	0.5871
C	-2.8810	4.9600	0.7980	C	-3.3960	4.7034	0.9840	C	-3.4419	4.6387	0.9658
C	0.8199	3.3118	-1.9569	C	0.5884	3.5769	-1.7049	C	0.5805	3.5932	-1.6748
C	6.1274	-0.0074	2.1304	C	5.9037	0.2120	2.3339	C	5.9070	0.2219	2.3400
C	-1.1724	-1.1594	1.3664	C	-1.1498	-1.2699	1.1817	C	-1.1278	-1.2709	1.1766
C	-0.9266	-0.6925	-0.0730	C	-0.9458	-0.6865	-0.2266	C	-0.9325	-0.6851	-0.2319
C	-2.2082	-1.0967	-0.7904	C	-2.1987	-1.1535	-0.9719	C	-2.1863	-1.1569	-0.9734
C	-3.0933	-1.8861	0.1663	C	-2.9613	-2.1416	-0.0902	C	-2.9479	-2.1392	-0.0853
C	-3.3559	-3.3039	-0.3999	C	-2.9870	-3.5811	-0.6740	C	-2.9842	-3.5765	-0.6710
C	-2.8877	-2.3846	2.6549	C	-2.7063	-2.7848	2.3547	C	-2.6830	-2.7789	2.3588
H	-0.0694	0.7863	-2.3023	H	-0.1288	1.0464	-2.3129	H	-0.1197	1.0632	-2.3125
H	3.3330	2.8567	-1.1652	H	3.0911	3.1988	-0.8374	H	3.0860	3.2192	-0.8116
H	4.0320	0.3495	-1.4427	H	3.9584	0.7718	-1.2970	H	3.9641	0.7970	-1.2905
H	5.0932	1.7874	0.2729	H	4.8476	2.1056	0.5912	H	4.8475	2.1219	0.6065
H	3.8333	1.3594	1.4275	H	3.5640	1.5031	1.6370	H	3.5642	1.5098	1.6473
H	5.7951	-0.5634	0.0852	H	5.7003	-0.1749	0.2336	H	5.7068	-0.1545	0.2374
H	4.7938	-2.3532	1.4469	H	4.7498	-2.1349	1.3805	H	4.7603	-2.1229	1.3737
H	3.6649	-1.2160	2.1732	H	3.5203	-1.1347	2.1443	H	3.5269	-1.1298	2.1405
H	2.5354	-2.4973	0.3905	H	2.5553	-2.3116	0.2036	H	2.5680	-2.2994	0.1926
H	3.7585	-2.0271	-0.7823	H	3.8001	-1.6711	-0.8614	H	3.8127	-1.6504	-0.8673
H	1.9572	-0.0887	0.7323	H	1.8163	0.0190	0.7215	H	1.8206	0.0257	0.7202
H	0.0372	1.0262	0.7396	H	-0.1539	1.0417	0.7378	H	-0.1583	1.0487	0.7374
H	-2.2003	1.7417	-1.2574	H	-2.3587	1.7320	-1.3017	H	-2.3473	1.7364	-1.3191
H	-1.5424	2.5966	1.6179	H	-1.8993	2.3955	1.6617	H	-1.9717	2.3086	1.6740
H	-3.6940	3.4040	-0.4495	H	-4.0089	3.2126	-0.4446	H	-4.0609	3.1295	-0.4468
H	-4.0651	-1.3822	0.2704	H	-4.0018	-1.7944	-0.0143	H	-3.9847	-1.7841	0.0006
H	-2.3936	-3.7874	-0.6210	H	-3.7852	-4.1443	-0.1809	H	-3.7738	-4.1417	-0.1664
H	-3.8706	-3.9044	0.3558	H	-3.2558	-3.4940	-1.7384	H	-3.2705	-3.4866	-1.7305
H	-3.7778	-2.6638	-2.1795	H	-1.0434	-3.7886	-0.8121	H	-1.0411	-3.7799	-0.8344
H	2.5335	-0.3512	-3.0597	H	2.5708	0.1572	-3.0476	H	2.5830	0.1865	-3.0465
H	2.2513	-2.0280	-2.5502	H	2.4002	-1.5757	-2.7050	H	2.4170	-1.5488	-2.7138
H	0.9654	-1.1154	-3.3452	H	1.0811	-0.6915	-3.4767	H	1.0966	-0.6644	-3.4827

H	-2.4145	5.0787	1.7817	H	-2.9938	4.7648	2.0009	H	-3.0454	4.7116	1.9842
H	-3.7787	5.5857	0.7660	H	-4.3401	5.2565	0.9543	H	-4.3795	5.2065	0.9210
H	-2.1817	5.3151	0.0353	H	-2.6897	5.1832	0.3001	H	-2.7319	5.1124	0.2802
H	1.5642	4.1122	-2.0053	H	1.2815	4.4214	-1.6459	H	1.2684	4.4413	-1.6067
H	0.4108	3.1667	-2.9658	H	0.2310	3.5016	-2.7406	H	0.2215	3.5282	-2.7106
H	-0.0105	3.6577	-1.3317	H	-0.2873	3.8126	-1.0902	H	-0.2956	3.8159	-1.0555
H	5.5675	0.2864	3.0270	H	5.2864	0.3921	3.2227	H	5.2882	0.3956	3.2291
H	6.8338	0.7991	1.9017	H	6.5699	1.0740	2.2125	H	6.5709	1.0865	2.2239
H	6.7116	-0.8999	2.3828	H	6.5286	-0.6653	2.5374	H	6.5341	-0.6547	2.5395
H	-3.8886	-1.9777	2.8371	H	-3.7648	-2.5902	2.5570	H	-3.7379	-2.5710	2.5660
H	-2.9500	-3.4768	2.6258	H	-2.5593	-3.8582	2.1998	H	-2.5491	-3.8546	2.2074
H	-2.2236	-2.0884	3.4671	H	-2.1100	-2.4601	3.2076	H	-2.0783	-2.4577	3.2071
H	-4.4690	2.2392	1.4265	H	-4.7721	1.8152	1.2710	H	-5.4093	3.1481	1.5076
N	-2.3458	-1.8532	1.4148	N	-2.2729	-2.0374	1.1872	N	-2.2478	-2.0411	1.1864
O	0.2676	-2.7031	-0.7772	O	0.3505	-2.5305	-1.1686	O	0.3656	-2.5187	-1.1883
O	-4.2330	3.1696	1.5549	O	-4.6283	2.7465	1.4947	O	-4.6316	2.5708	1.4825
O	-2.4757	-0.8991	-1.9617	O	-2.5346	-0.8320	-2.0910	O	-2.5196	-0.8452	-2.0959
O	-0.4260	-0.9372	2.3128	O	-0.4157	-1.0524	2.1396	O	-0.3880	-1.0533	2.1305
O	-4.2066	-3.2424	-1.5280	O	-1.7959	-4.2994	-0.4597	O	-1.7889	-4.2956	-0.4792

Table S7 (continued)

Conformer 4				Conformer 5				Conformer 6			
C	0.2705	-1.3514	-0.9128	C	0.2565	-1.2711	-0.9229	C	0.2727	-1.4890	-0.7369
C	1.2880	-0.3081	-1.3403	C	1.2784	-0.2204	-1.3224	C	1.3162	-0.5216	-1.2821
C	0.4457	1.0007	-1.3774	C	0.4553	1.0999	-1.2649	C	0.5386	0.8222	-1.3767
C	1.2760	2.2677	-1.2953	C	1.3063	2.3497	-1.1425	C	1.4293	2.0499	-1.4148
C	2.5509	2.2391	-0.8803	C	2.5918	2.2819	-0.7663	C	2.7198	1.9850	-1.0563
C	3.3011	1.0019	-0.4655	C	3.3355	1.0143	-0.4404	C	3.4301	0.7415	-0.5938
C	4.2037	1.2338	0.7644	C	4.2779	1.1676	0.7716	C	4.4034	1.0091	0.5732
C	5.0399	-0.0122	1.1082	C	5.1067	-0.1056	1.0210	C	5.1965	-0.2515	0.9625
C	4.1311	-1.2448	1.2677	C	4.1858	-1.3335	1.1421	C	4.2397	-1.4234	1.2481
C	3.2020	-1.4606	0.0613	C	3.2173	-1.4718	-0.0446	C	3.2428	-1.6716	0.1037
C	2.3674	-0.1958	-0.2024	C	2.3930	-0.1842	-0.2139	C	2.4548	-0.3877	-0.2075
C	1.8862	-0.6743	-2.7061	C	1.8310	-0.5213	-2.7232	C	1.8273	-1.0126	-2.6444
C	-0.6215	0.8419	-0.2404	C	-0.5796	0.8929	-0.1057	C	-0.4900	0.7962	-0.1943
C	-1.8448	1.7129	-0.3536	C	-1.7767	1.8052	-0.1183	C	-1.6653	1.7298	-0.3218
C	-2.3615	2.4154	0.6597	C	-2.1943	2.4896	0.9506	C	-2.1247	2.5008	0.6682
C	-3.6214	3.2411	0.5816	C	-3.4008	3.3883	0.9950	C	-3.3178	3.4108	0.5547
C	-3.3830	4.7059	0.9516	C	-4.3966	2.9344	2.0704	C	-2.9472	4.8755	0.8038
C	0.6207	3.5473	-1.7508	C	0.6594	3.6593	-1.5183	C	0.8148	3.3292	-1.9248
C	5.9085	0.2149	2.3497	C	6.0168	0.0424	2.2445	C	6.1356	0.0135	2.1437
C	-1.1857	-1.2429	1.1831	C	-1.1915	-1.2671	1.1814	C	-1.1475	-1.1712	1.3544
C	-0.9455	-0.6833	-0.2285	C	-0.9403	-0.6208	-0.1893	C	-0.9040	-0.7038	-0.0854
C	-2.1892	-1.1458	-0.9935	C	-2.1939	-1.0022	-0.9816	C	-2.1879	-1.1060	-0.8002
C	-2.9847	-2.1068	-0.1118	C	-3.0131	-1.9991	-0.1625	C	-3.0891	-1.8621	0.1672
C	-3.0197	-3.5541	-0.6740	C	-3.0819	-3.4045	-0.8198	C	-3.3955	-3.2744	-0.3887
C	-2.7931	-2.7089	2.3488	C	-2.8301	-2.7719	2.2510	C	-2.8836	-2.3491	2.6568
H	-0.1044	1.0124	-2.3293	H	-0.1238	1.1692	-2.1968	H	-0.0495	0.7986	-2.3056
H	3.1197	3.1697	-0.8709	H	3.1753	3.2025	-0.7265	H	3.3310	2.8849	-1.1343
H	3.9759	0.7310	-1.2954	H	3.9814	0.7795	-1.3038	H	4.0489	0.3830	-1.4342
H	4.8678	2.0876	0.5753	H	4.9479	2.0221	0.6087	H	5.0965	1.8142	0.2952
H	3.5781	1.5077	1.6264	H	3.6827	1.4018	1.6662	H	3.8355	1.3707	1.4428
H	5.7095	-0.2024	0.2548	H	5.7465	-0.2572	0.1375	H	5.8131	-0.5312	0.0939
H	4.7452	-2.1400	1.4276	H	4.7917	-2.2437	1.2342	H	4.8182	-2.3357	1.4409
H	3.5190	-1.1222	2.1731	H	3.6037	-1.2526	2.0717	H	3.6800	-1.2100	2.1705
H	2.5531	-2.3225	0.2468	H	2.5620	-2.3344	0.1140	H	2.5643	-2.4863	0.3756
H	3.8042	-1.7040	-0.8236	H	3.7888	-1.6743	-0.9597	H	3.7888	-2.0013	-0.7898
H	1.8251	0.0197	0.7285	H	1.8822	-0.0126	0.7435	H	1.9693	-0.0844	0.7307
H	-0.1397	1.0453	0.7212	H	-0.0603	1.0154	0.8503	H	0.0372	1.0239	0.7375
H	-2.3437	1.7293	-1.3221	H	-2.3315	1.8873	-1.0493	H	-2.1800	1.7353	-1.2825
H	-1.8674	2.3983	1.6339	H	-1.6458	2.4184	1.8899	H	-1.6293	2.4970	1.6400
H	-4.0234	3.1849	-0.4363	H	-3.0524	4.4022	1.2621	H	-3.7436	3.3159	-0.4567
H	-4.0198	-1.7403	-0.0605	H	-4.0394	-1.6128	-0.0868	H	-4.0427	-1.3258	0.2758
H	-3.8327	-4.1003	-0.1860	H	-3.9078	-3.9631	-0.3687	H	-2.4477	-3.7883	-0.6057
H	-3.2697	-3.4800	-1.7439	H	-3.3295	-3.2530	-1.8821	H	-3.9284	-3.8543	0.3705
H	-1.0754	-3.7863	-0.7723	H	-1.1414	-3.6730	-0.9248	H	-3.7995	-2.6220	-2.1663
H	2.5899	0.0921	-3.0408	H	2.5386	0.2507	-3.0361	H	2.5612	-0.3178	-3.0613
H	2.4072	-1.6337	-2.6697	H	2.3353	-1.4898	-2.7544	H	2.2885	-1.9997	-2.5634
H	1.0956	-0.7539	-3.4592	H	1.0179	-0.5461	-3.4559	H	0.9990	-1.0907	-3.3563
H	-2.9691	4.7889	1.9643	H	-4.7719	1.9323	1.8391	H	-2.5016	4.9954	1.7970

H	-4.3264	5.2603	0.9201	H	-5.2479	3.6234	2.1202	H	-3.8389	5.5121	0.7486
H	-2.6774	5.1800	0.2613	H	-3.9274	2.9116	3.0597	H	-2.2337	5.2357	0.0555
H	1.3155	4.3907	-1.6970	H	1.3645	4.4911	-1.4278	H	1.5522	4.1366	-1.9629
H	0.2691	3.4615	-2.7877	H	0.2952	3.6330	-2.5541	H	0.4061	3.1939	-2.9352
H	-0.2584	3.7914	-1.1442	H	-0.2112	3.8787	-0.8906	H	-0.0182	3.6596	-1.2944
H	5.2893	0.4118	3.2337	H	5.4287	0.1976	3.1575	H	5.5712	0.2982	3.0403
H	6.5797	1.0714	2.2174	H	6.6952	0.8969	2.1377	H	6.8381	0.8254	1.9224
H	6.5279	-0.6626	2.5684	H	6.6308	-0.8531	2.3948	H	6.7242	-0.8772	2.3923
H	-3.8514	-2.4882	2.5228	H	-3.8826	-2.5415	2.4465	H	-3.8600	-1.8913	2.8513
H	-2.6656	-3.7882	2.2189	H	-2.7268	-3.8419	2.0451	H	-3.0011	-3.4367	2.6286
H	-2.2087	-2.3796	3.2082	H	-2.2352	-2.5163	3.1281	H	-2.1952	-2.0863	3.4602
H	-4.3534	2.7510	2.3298	H	-4.7757	3.9981	-0.2418	H	-4.9995	3.5914	1.5370
N	-2.3184	-1.9948	1.1769	N	-2.3444	-1.9876	1.1296	N	-2.3309	-1.8462	1.4098
O	0.3510	-2.5517	-1.1188	O	0.3191	-2.4619	-1.1838	O	0.3027	-2.7000	-0.8040
O	-4.6491	2.6685	1.4100	O	-4.0088	3.4104	-0.2950	O	-4.2680	2.9559	1.5321
O	-2.4929	-0.8412	-2.1263	O	-2.4877	-0.6227	-2.0942	O	-2.4458	-0.9300	-1.9773
O	-0.4688	-1.0190	2.1531	O	-0.4670	-1.1289	2.1614	O	-0.3908	-0.9616	2.2959
O	-1.8408	-4.2841	-0.4294	O	-1.9211	-4.1773	-0.6261	O	-4.2422	-3.1952	-1.5189

Table S8. Cartesian coordinates of the six lowest-energy conformers of the 3'*R*,5'*S* stereoisomer of pyrenosetins E at the B3LYP/TZVP/SMD($CHCl_3$)//B3LYP/6-31+G(d,p)/SMD($CHCl_3$) level.

Conformer 1				Conformer 2				Conformer 3			
C	-0.2473	-1.4204	0.7068	C	-0.2179	-1.3681	0.8864	C	-0.2539	-1.4240	0.6934
C	-1.2537	-0.4161	1.2469	C	-1.3060	-0.3897	1.3144	C	-1.2594	-0.4208	1.2391
C	-0.4325	0.9048	1.3168	C	-0.5768	0.9846	1.2996	C	-0.4363	0.8992	1.3159
C	-1.2809	2.1614	1.3367	C	-1.5097	2.1767	1.2028	C	-1.2822	2.1572	1.3368
C	-2.5783	2.1322	0.9993	C	-2.7900	2.0319	0.8310	C	-2.5801	2.1315	1.0013
C	-3.3357	0.9052	0.5704	C	-3.4484	0.7261	0.4764	C	-3.3404	0.9066	0.5711
C	-4.3208	1.1884	-0.5833	C	-4.4091	0.8485	-0.7247	C	-4.3278	1.1954	-0.5791
C	-5.1618	-0.0495	-0.9420	C	-5.1513	-0.4703	-1.0060	C	-5.1706	-0.0402	-0.9416
C	-4.2481	-1.2544	-1.2295	C	-4.1489	-1.6274	-1.1684	C	-4.2581	-1.2440	-1.2372
C	-3.2401	-1.5212	-0.0991	C	-3.1645	-1.7326	0.0085	C	-3.2482	-1.5169	-0.1100
C	-2.4040	-0.2612	0.1846	C	-2.4268	-0.3981	0.2135	C	-2.4104	-0.2591	0.1790
C	-1.7620	-0.8745	2.6235	C	-1.8273	-0.7708	2.7087	C	-1.7668	-0.8860	2.6139
C	0.5978	0.8088	0.1377	C	0.4882	0.8748	0.1523	C	0.5977	0.8054	0.1406
C	1.7805	1.7370	0.2107	C	1.6096	1.8785	0.1903	C	1.7866	1.7253	0.2145
C	2.1949	2.5127	-0.7973	C	1.9938	2.6214	-0.8538	C	2.2164	2.4783	-0.8021
C	3.4045	3.4099	-0.7405	C	3.1465	3.5918	-0.8306	C	3.4254	3.3728	-0.7496
C	3.0685	4.8663	-1.0443	C	2.7302	5.0075	-1.2165	C	3.0626	4.8439	-0.9722
C	-0.6210	3.4284	1.8204	C	-0.9543	3.5193	1.6075	C	-0.6172	3.4230	1.8168
C	-6.1134	0.2316	-2.1093	C	-6.0796	-0.3499	-2.2188	C	-6.1250	0.2470	-2.1051
C	2.2098	-1.0669	1.0036	C	2.2043	-0.8566	1.1297	C	2.1974	-1.0735	1.0199
C	0.9937	-0.6968	0.1321	C	0.9749	-0.5994	0.2436	C	0.9921	-0.7000	0.1342
C	1.3655	-1.2682	-1.2306	C	1.3688	-1.2329	-1.0819	C	1.3804	-1.2662	-1.2260
C	2.6237	-2.1220	-1.0928	C	2.6448	-2.0524	-0.8846	C	2.6418	-2.1127	-1.0781
C	2.3639	-3.6317	-1.3480	C	2.3754	-3.5452	-1.1056	C	2.3924	-3.6193	-1.3553
C	4.2730	-2.4062	0.8213	C	4.3310	-2.1013	1.0373	C	4.2693	-2.4002	0.8534
H	0.1583	0.8760	2.2423	H	-0.0110	1.0671	2.2376	H	0.1512	0.8665	2.2432
H	-3.1594	3.0523	1.0716	H	-3.4340	2.9120	0.8164	H	-3.1587	3.0531	1.0744
H	-3.9504	0.5787	1.4265	H	-4.0694	0.4245	1.3372	H	-3.9533	0.5777	1.4276
H	-4.9811	2.0197	-0.3035	H	-5.1341	1.6506	-0.5334	H	-4.9870	2.0260	-0.2943
H	-3.7572	1.5204	-1.4676	H	-3.8388	1.1481	-1.6161	H	-3.7660	1.5308	-1.4632
H	-5.7708	-0.2988	-0.0590	H	-5.7717	-0.6913	-0.1233	H	-5.7775	-0.2935	-0.0583
H	-4.8580	-2.1504	-1.4006	H	-4.6912	-2.5743	-1.2844	H	-4.8686	-2.1388	-1.4118
H	-3.6992	-1.0697	-2.1648	H	-3.5820	-1.4786	-2.0993	H	-3.7108	-1.0547	-2.1725
H	-2.5958	-2.3628	-0.3708	H	-2.4542	-2.5448	-0.1746	H	-2.6048	-2.3575	-0.3868
H	-3.7817	-1.8206	0.8074	H	-3.7155	-1.9977	0.9201	H	-3.7886	-1.8204	0.7959
H	-1.9232	0.0195	-0.7636	H	-1.9342	-0.1607	-0.7404	H	-1.9299	0.0258	-0.7681
H	0.0715	0.9890	-0.8061	H	-0.0251	0.9657	-0.8112	H	0.0745	0.9871	-0.8049
H	2.3339	1.7389	1.1493	H	2.1455	1.9653	1.1348	H	2.3375	1.7273	1.1541
H	1.6567	2.5114	-1.7467	H	1.4743	2.5339	-1.8096	H	1.6830	2.4709	-1.7537
H	3.8509	3.3452	0.2642	H	3.5802	3.6074	0.1817	H	3.9049	3.2673	0.2363
H	3.3547	-1.7780	-1.8380	H	3.3996	-1.7314	-1.6164	H	3.3827	-1.7538	-1.8062
H	1.7833	-3.7063	-2.2807	H	1.6927	-3.9067	-0.3261	H	1.8336	-3.6848	-2.3019
H	3.3236	-4.1284	-1.5205	H	1.8780	-3.6521	-2.0807	H	3.3565	-4.1132	-1.5107
H	0.9340	-3.8318	-0.0195	H	3.4361	-5.1771	-1.0674	H	0.9365	-3.8309	-0.0559
H	-2.4573	-0.1429	3.0433	H	-2.5882	-0.0632	3.0485	H	-2.4608	-0.1559	3.0385
H	-2.2680	-1.8406	2.5606	H	-2.2584	-1.7747	2.7117	H	-2.2738	-1.8512	2.5465
H	-0.9249	-0.9799	3.3209	H	-1.0090	-0.7576	3.4358	H	-0.9289	-0.9959	3.3096
H	2.5994	4.9546	-2.0301	H	2.2700	5.0151	-2.2103	H	2.5635	4.9748	-1.9382
H	3.9820	5.4694	-1.0436	H	3.6069	5.6627	-1.2394	H	3.9655	5.4671	-0.9651

H	2.3829	5.2720	-0.2945	H	2.0123	5.4117	-0.4967	H	2.3980	5.2109	-0.1833
H	-1.3327	4.2588	1.8532	H	-1.7209	4.2984	1.5561	H	-1.3265	4.2554	1.8517
H	-0.2068	3.2948	2.8287	H	-0.5671	3.4899	2.6348	H	-0.1987	3.2894	2.8232
H	0.2155	3.7229	1.1776	H	-0.1176	3.8228	0.9692	H	0.2172	3.7142	1.1695
H	-5.5570	0.4879	-3.0194	H	-5.5113	-0.1276	-3.1306	H	-5.5707	0.5074	-3.0154
H	-6.7856	1.0680	-1.8851	H	-6.8142	0.4522	-2.0824	H	-6.7962	1.0826	-1.8753
H	-6.7345	-0.6427	-2.3364	H	-6.6318	-1.2809	-2.3918	H	-6.7470	-0.6259	-2.3349
H	4.1534	-3.4863	0.9518	H	4.3524	-3.1787	1.2144	H	4.1671	-3.4843	0.9650
H	5.1246	-2.2137	0.1607	H	5.1459	-1.8470	0.3513	H	5.1263	-2.1818	0.2080
H	4.4566	-1.9459	1.7923	H	4.4666	-1.5683	1.9787	H	4.4316	-1.9525	1.8341
H	4.5829	2.0806	-1.5585	H	4.4065	2.2904	-1.5679	H	5.0538	3.5340	-1.8211
N	3.0654	-1.8225	0.2627	N	3.0552	-1.6903	0.4696	N	3.0598	-1.8284	0.2880
O	-0.3725	-2.6339	0.7193	O	-0.2312	-2.5730	1.0332	O	-0.3835	-2.6372	0.6929
O	4.3664	3.0086	-1.7317	O	4.1464	3.1973	-1.7865	O	4.3137	2.9100	-1.7802
O	0.7634	-1.0855	-2.2662	O	0.7755	-1.1208	-2.1326	O	0.7846	-1.0871	-2.2658
O	2.3681	-0.7322	2.1734	O	2.3719	-0.3973	2.2560	O	2.3400	-0.7431	2.1934
O	1.7579	-4.2933	-0.2633	O	3.6233	-4.2276	-1.0801	O	1.7616	-4.2940	-0.2921

Table S8 (continued)

Conformer 4				Conformer 5				Conformer 6			
C	-0.2906	-1.3291	1.0058	C	-0.2601	-1.3373	0.7211	C	-0.2618	-1.3552	0.7040
C	-1.3091	-0.2526	1.3651	C	-1.2611	-0.3081	1.2259	C	-1.2661	-0.3367	1.2246
C	-0.4844	1.0641	1.2806	C	-0.4484	1.0208	1.1951	C	-0.4534	0.9922	1.2239
C	-1.3281	2.3119	1.1019	C	-1.3061	2.2707	1.1575	C	-1.3087	2.2438	1.2091
C	-2.6132	2.2365	0.7262	C	-2.6126	2.2124	0.8613	C	-2.6134	2.1933	0.9033
C	-3.3609	0.9622	0.4418	C	-3.3722	0.9562	0.5324	C	-3.3726	0.9442	0.5460
C	-4.3039	1.0827	-0.7735	C	-4.3949	1.1624	-0.6046	C	-4.3893	1.1731	-0.5920
C	-5.1365	-0.1942	-0.9859	C	-5.2351	-0.1008	-0.8628	C	-5.2300	-0.0836	-0.8784
C	-4.2182	-1.4266	-1.0721	C	-4.3190	-1.3134	-1.1066	C	-4.3145	-1.2928	-1.1409
C	-3.2507	-1.5333	0.1186	C	-3.2754	-1.5051	0.0064	C	-3.2766	-1.5071	-0.0267
C	-2.4206	-0.2451	0.2543	C	-2.4420	-0.2248	0.1901	C	-2.4424	-0.2317	0.1856
C	-1.8655	-0.5155	2.7733	C	-1.7273	-0.6875	2.6412	C	-1.7386	-0.7445	2.6298
C	0.5820	0.8134	0.1570	C	0.5561	0.8550	0.0011	C	0.5531	0.8508	0.0295
C	1.7689	1.7389	0.1513	C	1.7287	1.7971	-0.0308	C	1.7172	1.8035	0.0149
C	2.2229	2.3811	-0.9308	C	2.0679	2.5313	-1.0949	C	2.0543	2.5393	-1.0483
C	3.4403	3.2692	-0.9448	C	3.2604	3.4570	-1.1691	C	3.2251	3.4822	-1.1229
C	3.1339	4.6783	-1.4418	C	4.2996	2.9541	-2.1721	C	4.1858	3.0918	-2.2539
C	-0.6805	3.6335	1.4321	C	-0.6446	3.5715	1.5399	C	-0.6458	3.5328	1.6269
C	-6.0476	-0.0786	-2.2122	C	-6.2250	0.1030	-2.0141	C	-6.2141	0.1435	-2.0305
C	2.1574	-0.9533	1.2885	C	2.1883	-0.9300	1.0014	C	2.1834	-0.9469	1.0113
C	0.9688	-0.6828	0.3516	C	0.9717	-0.6415	0.0987	C	0.9736	-0.6456	0.1040
C	1.3569	-1.4181	-0.9179	C	1.3608	-1.3007	-1.2182	C	1.3760	-1.2781	-1.2215
C	2.6276	-2.2197	-0.6739	C	2.6342	-2.1180	-1.0152	C	2.6513	-2.0938	-1.0242
C	2.3423	-3.7296	-0.8730	C	2.4010	-3.6454	-1.1679	C	2.4239	-3.6184	-1.2086
C	4.2411	-2.2787	1.2970	C	4.2825	-2.2297	0.9173	C	4.2821	-2.2390	0.9207
H	0.0769	1.1620	2.2198	H	0.1632	1.0532	2.1065	H	0.1566	1.0059	2.1367
H	-3.1925	3.1577	0.6536	H	-3.1988	3.1316	0.8933	H	-3.1990	3.1122	0.9502
H	-4.0067	0.7563	1.3125	H	-3.9568	0.6787	1.4262	H	-3.9623	0.6499	1.4310
H	-4.9709	1.9438	-0.6349	H	-5.0540	2.0041	-0.3542	H	-5.0486	2.0110	-0.3293
H	-3.7088	1.2896	-1.6750	H	-3.8619	1.4452	-1.5242	H	-3.8513	1.4725	-1.5035
H	-5.7757	-0.3192	-0.0979	H	-5.8142	-0.3018	0.0521	H	-5.8138	-0.3012	0.0296
H	-4.8257	-2.3380	-1.1390	H	-4.9251	-2.2227	-1.2061	H	-4.9215	-2.1991	-1.2607
H	-3.6367	-1.3725	-2.0044	H	-3.8001	-1.1801	-2.0674	H	-3.7908	-1.1420	-2.0965
H	-2.5993	-2.4031	-0.0108	H	-2.6319	-2.3566	-0.2342	H	-2.6328	-2.3547	-0.2801
H	-3.8246	-1.7054	1.0384	H	-3.7871	-1.7541	0.9449	H	-3.7934	-1.7732	0.9044
H	-1.9059	-0.0991	-0.7064	H	-1.9900	0.0020	-0.7864	H	-1.9861	0.0132	-0.7844
H	0.0857	0.8762	-0.8175	H	0.0022	0.9560	-0.9389	H	0.0000	0.9624	-0.9100
H	2.2923	1.8546	1.0999	H	2.3450	1.8411	0.8647	H	2.3171	1.8663	0.9188
H	1.7160	2.2651	-1.8904	H	1.4634	2.4849	-2.0018	H	1.4590	2.4826	-1.9599
H	3.8509	3.3285	0.0754	H	2.9002	4.4403	-1.5185	H	2.8290	4.4889	-1.3474
H	3.4006	-1.9260	-1.3976	H	3.3649	-1.8121	-1.7770	H	3.3867	-1.7703	-1.7742
H	3.2158	-4.3087	-0.5596	H	1.8353	-3.7941	-2.1009	H	1.8679	-3.7504	-2.1500
H	1.4933	-4.0224	-0.2389	H	3.3699	-4.1387	-1.2918	H	3.3953	-4.1071	-1.3322

H	1.3875	-3.4636	-2.5396	H	0.9519	-3.7761	0.1483	H	0.9624	-3.7770	0.0911
H	-2.5746	0.2640	3.0644	H	-2.4180	0.0621	3.0363	H	-2.4305	-0.0024	3.0368
H	-2.3690	-1.4835	2.8280	H	-2.2256	-1.6597	2.6498	H	-2.2375	-1.7164	2.6171
H	-1.0531	-0.5203	3.5071	H	-0.8703	-0.7444	3.3198	H	-0.8846	-0.8152	3.3108
H	2.6998	4.6451	-2.4468	H	4.6922	1.9839	-1.8509	H	4.6071	2.0990	-2.0659
H	4.0545	5.2689	-1.4849	H	5.1307	3.6626	-2.2416	H	5.0088	3.8128	-2.3248
H	2.4286	5.1815	-0.7738	H	3.8571	2.8387	-3.1662	H	3.6723	3.0759	-3.2211
H	-1.3900	4.4599	1.3287	H	-1.3652	4.3947	1.5514	H	-1.3578	4.3638	1.6256
H	-0.3006	3.6377	2.4625	H	-0.1906	3.5023	2.5374	H	-0.2256	3.4458	2.6378
H	0.1780	3.8398	0.7838	H	0.1622	3.8359	0.8477	H	0.1885	3.7958	0.9678
H	-5.4603	0.0492	-3.1300	H	-5.6997	0.3086	-2.9551	H	-5.6838	0.3664	-2.9647
H	-6.7237	0.7802	-2.1289	H	-6.8977	0.9463	-1.8189	H	-6.8866	0.9838	-1.8224
H	-6.6641	-0.9765	-2.3363	H	-6.8447	-0.7878	-2.1689	H	-6.8341	-0.7433	-2.2055
H	4.2162	-3.3494	1.5228	H	4.1924	-3.3006	1.1255	H	4.1933	-3.3142	1.1057
H	5.0859	-2.0773	0.6293	H	5.1287	-2.0620	0.2433	H	5.1350	-2.0548	0.2594
H	4.3727	-1.7241	2.2263	H	4.4534	-1.6955	1.8521	H	4.4415	-1.7239	1.8682
H	4.6248	1.8351	-1.5481	H	3.3017	3.9804	0.7101	H	4.6377	4.1042	0.0643
N	2.9995	-1.8381	0.6817	N	3.0606	-1.7199	0.3194	N	3.0650	-1.7202	0.3211
O	-0.3948	-2.5210	1.2058	O	-0.3838	-2.5489	0.7946	O	-0.3849	-2.5684	0.7514
O	4.4309	2.7373	-1.8420	O	3.9338	3.6042	0.0799	O	3.8900	3.4945	0.1385
O	0.7326	-1.4222	-1.9626	O	0.7593	-1.2059	-2.2660	O	0.7831	-1.1659	-2.2727
O	2.3068	-0.4663	2.4045	O	2.3317	-0.5134	2.1468	O	2.3156	-0.5545	2.1663
O	2.1260	-4.0187	-2.2406	O	1.7877	-4.2388	-0.0478	O	1.8008	-4.2346	-0.1064

Table S9. Cartesian coordinates of the six lowest-energy conformers of the 3*S*,5'*S* stereoisomer of pyrenosetins E at the B3LYP/TZVP/SMD($CHCl_3$)//B3LYP/6-31+G(d,p)/SMD($CHCl_3$) level.

Conformer 1				Conformer 2				Conformer 3			
C	-0.5355	-1.1797	1.2984	C	-0.5237	-1.3175	1.1506	C	-0.5095	-1.3077	1.1360
C	-1.5227	-0.0260	1.4371	C	-1.4995	-0.1742	1.4008	C	-1.5146	-0.1904	1.3938
C	-0.6372	1.2261	1.1898	C	-0.6091	1.0917	1.2550	C	-0.6555	1.0985	1.2576
C	-1.4095	2.4602	0.7629	C	-1.3815	2.3616	0.9500	C	-1.4576	2.3514	0.9608
C	-2.6769	2.3758	0.3339	C	-2.6550	2.3195	0.5323	C	-2.7298	2.2823	0.5431
C	-3.4677	1.1001	0.2174	C	-3.4543	1.0631	0.3135	C	-3.4987	1.0084	0.3161
C	-4.3380	1.0574	-1.0562	C	-4.3439	1.1379	-0.9452	C	-4.3905	1.0711	-0.9418
C	-5.2089	-0.2104	-1.1187	C	-5.2254	-0.1140	-1.1044	C	-5.2415	-0.2004	-1.1106
C	-4.3386	-1.4702	-0.9575	C	-4.3621	-1.3882	-1.0683	C	-4.3477	-1.4536	-1.0843
C	-3.4439	-1.4192	0.2921	C	-3.4475	-1.4527	0.1662	C	-3.4315	-1.5054	0.1496
C	-2.5714	-0.1528	0.2748	C	-2.5658	-0.1947	0.2467	C	-2.5801	-0.2273	0.2397
C	-2.1619	-0.0413	2.8325	C	-2.1209	-0.3105	2.7985	C	-2.1327	-0.3524	2.7902
C	0.4722	0.7814	0.1763	C	0.4868	0.7284	0.1953	C	0.4454	0.7678	0.1950
C	1.7461	1.5829	0.2389	C	1.7460	1.5529	0.2627	C	1.6885	1.6155	0.2567
C	2.5201	1.8706	-0.8106	C	2.4417	1.9739	-0.7981	C	2.3879	2.0169	-0.8093
C	3.8669	2.5253	-0.6755	C	3.7752	2.6719	-0.7006	C	3.7353	2.6843	-0.7122
C	4.0965	3.6464	-1.6856	C	3.8402	3.9424	-1.5410	C	3.8650	3.9157	-1.6007
C	-0.6984	3.7854	0.8769	C	-0.6683	3.6703	1.1809	C	-0.7725	3.6743	1.1956
C	-6.0441	-0.2593	-2.4022	C	-6.0812	-0.0455	-2.3734	C	-6.0988	-0.1426	-2.3791
C	0.8178	-1.6113	-0.7813	C	0.9222	-1.5989	-0.8869	C	0.9682	-1.5301	-0.9010
C	0.7056	-0.7310	0.4734	C	0.7423	-0.7940	0.4046	C	0.7433	-0.7458	0.3987
C	1.9633	-1.1030	1.2580	C	1.9592	-1.1947	1.2344	C	1.9647	-1.1177	1.2346
C	2.6306	-2.3032	0.5916	C	2.6767	-2.3549	0.5363	C	2.6738	-2.2999	0.5649
C	4.1407	-2.0953	0.3050	C	4.1818	-2.0898	0.3764	C	4.1924	-2.1308	0.4856
C	2.1581	-3.5114	-1.5973	C	2.2507	-3.5207	-1.6916	C	2.4179	-3.3395	-1.7459
H	-0.1241	1.4559	2.1354	H	-0.0834	1.2350	2.2105	H	-0.1331	1.2477	2.2141
H	-3.2026	3.2935	0.0672	H	-3.1811	3.2592	0.3597	H	-3.2775	3.2103	0.3745
H	-4.1669	1.0605	1.0703	H	-4.1409	0.9506	1.1698	H	-4.1821	0.8729	1.1719
H	-4.9763	1.9502	-1.0942	H	-4.9757	2.0346	-0.8946	H	-5.0439	1.9518	-0.8841
H	-3.6861	1.0999	-1.9409	H	-3.7053	1.2541	-1.8330	H	-3.7552	1.2097	-1.8287
H	-5.9017	-0.1743	-0.2632	H	-5.9042	-0.1487	-0.2379	H	-5.9193	-0.2582	-0.2444
H	-4.9789	-2.3607	-0.9207	H	-5.0081	-2.2748	-1.0988	H	-4.9722	-2.3553	-1.1215
H	-3.7026	-1.5808	-1.8478	H	-3.7418	-1.4243	-1.9758	H	-3.7270	-1.4678	-1.9922
H	-2.8203	-2.3179	0.3355	H	-2.8307	-2.3558	0.1215	H	-2.7932	-2.3931	0.0983
H	-4.0732	-1.4291	1.1918	H	-4.0632	-1.5367	1.0712	H	-4.0450	-1.6109	1.0539
H	-1.9989	-0.1795	-0.6626	H	-2.0082	-0.1414	-0.6989	H	-2.0240	-0.1532	-0.7054
H	0.0681	0.8267	-0.8401	H	0.0551	0.8196	-0.8069	H	0.0092	0.8482	-0.8061
H	2.0813	1.8744	1.2332	H	2.1342	1.7557	1.2602	H	2.0713	1.8380	1.2519
H	2.2270	1.5705	-1.8171	H	2.0865	1.7638	-1.8087	H	2.0411	1.7823	-1.8169
H	3.9714	2.9206	0.3451	H	3.9586	2.9308	0.3549	H	3.9060	2.9789	0.3366
H	2.5373	-3.1569	1.2807	H	2.5455	-3.2626	1.1448	H	2.4797	-3.1909	1.1827
H	4.5937	-1.6975	1.2236	H	4.5971	-1.8712	1.3633	H	4.5511	-1.8952	1.4966
H	4.5872	-3.0791	0.1216	H	4.6798	-2.9880	-0.0108	H	4.6462	-3.0801	0.1774
H	4.4478	-0.3539	-0.6045	H	4.1100	-1.1419	-1.3364	H	5.4491	-1.1933	-0.6768
H	-2.8546	0.7953	2.9568	H	-2.8091	0.5137	3.0036	H	-2.8413	0.4529	3.0012
H	-2.7051	-0.9722	3.0108	H	-2.6645	-1.2525	2.9019	H	-2.6528	-1.3082	2.8868
H	-1.3917	0.0459	3.6060	H	-1.3401	-0.2923	3.5659	H	-1.3528	-0.3201	3.5581
H	3.9955	3.2689	-2.7085	H	3.6414	3.7161	-2.5943	H	3.6679	3.6568	-2.6466
H	5.0997	4.0735	-1.5726	H	4.8361	4.3912	-1.4697	H	4.8787	4.3241	-1.5345
H	3.3703	4.4527	-1.5385	H	3.1025	4.6743	-1.1972	H	3.1571	4.6923	-1.2941
H	-1.3528	4.6139	0.5895	H	-1.3266	4.5216	0.9829	H	-1.4488	4.5122	1.0009
H	-0.3553	3.9632	1.9050	H	-0.3123	3.7486	2.2168	H	-0.4170	3.7569	2.2314
H	0.1939	3.8230	0.2409	H	0.2159	3.7689	0.5407	H	0.1086	3.7938	0.5545
H	-5.4009	-0.2952	-3.2902	H	-5.4525	-0.0093	-3.2718	H	-5.4713	-0.0840	-3.2771
H	-6.6883	0.6230	-2.4948	H	-6.7189	0.8461	-2.3787	H	-6.7580	0.7333	-2.3774
H	-6.6888	-1.1456	-2.4245	H	-6.7338	-0.9218	-2.4615	H	-6.7300	-1.0338	-2.4743
H	3.0938	-3.2598	-2.1064	H	3.2631	-3.4309	-2.0993	H	3.4103	-3.0812	-2.1294
H	2.2586	-4.4916	-1.1196	H	2.1497	-4.5029	-1.2164	H	2.4408	-4.3578	-1.3428
H	1.3506	-3.5477	-2.3289	H	1.5324	-3.4377	-2.5073	H	1.6977	-3.2914	-2.5628
H	5.7235	1.8400	-0.8000	H	4.7553	0.9754	-0.6785	H	4.5584	0.9092	-0.6971
N	1.8257	-2.5043	-0.6056	N	1.9668	-2.4644	-0.7330	N	2.0002	-2.3989	-0.7224
O	-0.6541	-2.2943	1.7635	O	-0.6597	-2.4759	1.4850	O	-0.6181	-2.4715	1.4627
O	4.8328	1.4651	-0.8737	O	4.8137	1.8066	-1.1813	O	4.7497	1.7588	-1.1319
O	2.3812	-0.5577	2.2574	O	2.3160	-0.7066	2.2839	O	2.3249	-0.6041	2.2710
O	0.0821	-1.5308	-1.7611	O	0.2130	-1.4995	-1.8815	O	0.2980	-1.4039	-1.9204
O	4.3968	-1.3029	-0.8289	O	4.4496	-0.9617	-0.4469	O	4.5131	-1.0992	-0.4464

Table S9 (continued)

Conformer 4				Conformer 5				Conformer 6			
C	-0.5395	-1.2371	1.2423	C	0.3665	-1.5321	-0.7966	C	-0.5343	-1.4961	0.9004
C	-1.5240	-0.0870	1.4232	C	1.3744	-0.5091	-1.3070	C	-1.5196	-0.4076	1.3214
C	-0.6382	1.1730	1.2170	C	0.5555	0.8135	-1.3446	C	-0.6471	0.8824	1.3355
C	-1.4112	2.4211	0.8342	C	1.4094	2.0676	-1.3395	C	-1.4363	2.1714	1.2090
C	-2.6800	2.3516	0.4063	C	2.7041	2.0275	-0.9926	C	-2.7170	2.1699	0.8124
C	-3.4722	1.0813	0.2493	C	3.4548	0.7895	-0.5829	C	-3.5090	0.9435	0.4466
C	-4.3475	1.0825	-1.0217	C	4.4276	1.0416	0.5881	C	-4.4244	1.1738	-0.7742
C	-5.2207	-0.1813	-1.1232	C	5.2616	-0.2079	0.9239	C	-5.2990	-0.0547	-1.0821
C	-4.3516	-1.4469	-1.0076	C	4.3430	-1.4188	1.1690	C	-4.4253	-1.3137	-1.2289
C	-3.4519	-1.4389	0.2394	C	3.3462	-1.6532	0.0216	C	-3.4866	-1.5301	-0.0304
C	-2.5774	-0.1738	0.2612	C	2.5169	-0.3832	-0.2350	C	-2.6115	-0.2856	0.1982
C	-2.1586	-0.1501	2.8198	C	1.8916	-0.9309	-2.6908	C	-2.1099	-0.7424	2.6997
C	0.4685	0.7598	0.1878	C	-0.4684	0.7007	-0.1619	C	0.4268	0.6656	0.2218
C	1.7372	1.5694	0.2530	C	-1.6498	1.6335	-0.2180	C	1.6661	1.5194	0.2781
C	2.4884	1.9015	-0.8011	C	-2.0677	2.3813	0.8097	C	2.2996	1.9800	-0.8057
C	3.8354	2.5639	-0.6756	C	-3.2673	3.2927	0.7705	C	3.6449	2.6589	-0.7839
C	4.0188	3.7527	-1.6135	C	-2.9096	4.7431	1.0801	C	3.6530	3.9851	-1.5361
C	-0.7011	3.7421	0.9941	C	0.7564	3.3458	-1.8026	C	-0.7278	3.4470	1.5909
C	-6.0610	-0.1863	-2.4043	C	6.1999	0.0404	2.1093	C	-6.1780	0.1723	-2.3163
C	0.8670	-1.6024	-0.8188	C	-1.1197	-1.3918	1.2466	C	1.1095	-1.4741	-1.0531
C	0.7128	-0.7557	0.4529	C	-0.8537	-0.8033	-0.1447	C	0.7496	-0.8554	0.3003
C	1.9545	-1.1312	1.2602	C	-2.0816	-1.2306	-0.9312	C	1.9225	-1.2587	1.1867
C	2.6487	-2.3126	0.5858	C	-2.8419	-2.2822	-0.1304	C	2.7975	-2.2602	0.4287
C	4.1603	-2.0793	0.3453	C	-4.3554	-1.9741	-0.1340	C	4.2900	-1.9136	0.5625
C	2.2495	-3.4663	-1.6471	C	-2.6134	-3.0622	2.2871	C	2.7783	-3.0204	-2.0046
H	-0.1229	1.3705	2.1686	H	-0.0362	0.8097	-2.2714	H	-0.1077	0.9065	2.2934
H	-3.2065	3.2781	0.1738	H	3.2882	2.9473	-1.0419	H	-3.2541	3.1182	0.7707
H	-4.1680	1.0132	1.1030	H	4.0783	0.4829	-1.4402	H	-4.1769	0.7114	1.2937
H	-4.9846	1.9770	-1.0272	H	5.0936	1.8778	0.3372	H	-5.0621	2.0493	-0.5932
H	-3.6990	1.1538	-1.9070	H	3.8549	1.3520	1.4742	H	-3.8046	1.4137	-1.6505
H	-5.9100	-0.1729	-0.2643	H	5.8807	-0.4348	0.0418	H	-5.9617	-0.2103	-0.2163
H	-4.9930	-2.3373	-0.9977	H	4.9504	-2.3198	1.3225	H	-5.0649	-2.1953	-1.3633
H	-3.7197	-1.5288	-1.9040	H	3.7839	-1.2588	2.1025	H	-3.8231	-1.2247	-2.1449
H	-2.8299	-2.3395	0.2510	H	2.6954	-2.4987	0.2652	H	-2.8650	-2.4140	-0.2029
H	-4.0779	-1.4773	1.1404	H	3.8960	-1.9307	-0.8872	H	-4.0846	-1.7362	0.8669
H	-2.0087	-0.1695	-0.6791	H	2.0299	-0.1297	0.7170	H	-2.0750	-0.1021	-0.7436
H	0.0558	0.8208	-0.8245	H	0.0622	0.8751	0.7798	H	-0.0468	0.8070	-0.7554
H	2.0885	1.8314	1.2501	H	-2.1956	1.6720	-1.1602	H	2.1006	1.6893	1.2631
H	2.1689	1.6366	-1.8109	H	-1.5361	2.3481	1.7621	H	1.8832	1.7981	-1.7979
H	3.9805	2.8855	0.3610	H	-3.7237	3.2411	-0.2307	H	3.9326	2.8429	0.2650
H	2.5461	-3.1841	1.2507	H	-2.6910	-3.2687	-0.5941	H	2.6491	-3.2616	0.8645
H	4.5941	-1.7376	1.2946	H	-4.8726	-2.6662	0.5367	H	4.6152	-2.2095	1.5660
H	4.6201	-3.0439	0.1042	H	-4.5159	-0.9536	0.2484	H	4.8705	-2.4938	-0.1593
H	4.4602	-0.2780	-0.4385	H	-4.4013	-1.5814	-2.0231	H	4.3642	-0.0502	1.1325
H	-2.8513	0.6813	2.9746	H	2.6003	-0.1969	-3.0833	H	-2.8000	0.0387	3.0298
H	-2.7005	-1.0870	2.9681	H	2.3850	-1.9048	-2.6508	H	-2.6445	-1.6950	2.6830
H	-1.3856	-0.0887	3.5929	H	1.0622	-1.0071	-3.4016	H	-1.3134	-0.8211	3.4470
H	3.8805	3.4503	-2.6590	H	-2.4271	4.8187	2.0604	H	3.3452	3.8365	-2.5767
H	5.0228	4.1744	-1.5050	H	-3.8159	5.3568	1.0951	H	4.6610	4.4123	-1.5351
H	3.2864	4.5361	-1.3926	H	-2.2288	5.1458	0.3243	H	2.9696	4.7013	-1.0693
H	-1.3568	4.5795	0.7371	H	1.4706	4.1746	-1.8148	H	-1.3976	4.3094	1.5213
H	-0.3569	3.8835	2.0274	H	0.3482	3.2336	-2.8160	H	-0.3459	3.3945	2.6192
H	0.1905	3.8031	0.3590	H	-0.0831	3.6304	-1.1590	H	0.1381	3.6387	0.9464
H	-5.4214	-0.1939	-3.2956	H	5.6330	0.2739	3.0191	H	-5.5659	0.3350	-3.2120
H	-6.7039	0.6996	-2.4651	H	6.8763	0.8809	1.9153	H	-6.8237	1.0495	-2.1922
H	-6.7073	-1.0703	-2.4533	H	6.8165	-0.8409	2.3208	H	-6.8243	-0.6918	-2.5096
H	3.1909	-3.1872	-2.1312	H	-3.5978	-2.7783	2.6727	H	3.7734	-2.6804	-2.3086
H	2.3578	-4.4564	-1.1924	H	-2.6463	-4.1087	1.9654	H	2.8396	-4.0707	-1.6978
H	1.4580	-3.4982	-2.3963	H	-1.8773	-2.9512	3.0836	H	2.0999	-2.9306	-2.8532
H	4.9083	1.3892	-1.8434	H	-4.4447	1.9704	1.6037	H	4.5749	0.9457	-0.9881
N	1.8785	-2.4907	-0.6380	N	-2.2086	-2.2099	1.1804	N	2.2509	-2.2071	-0.9223
O	-0.6665	-2.3712	1.6549	O	0.4432	-2.7389	-0.8848	O	-0.6774	-2.6942	1.0281
O	4.8842	1.5840	-0.8925	O	-4.2249	2.8992	1.7679	O	4.6133	1.8162	-1.4230
O	2.3456	-0.5994	2.2774	O	-2.4420	-0.8013	-2.0127	O	2.1678	-0.8498	2.3032
O	0.1593	-1.5023	-1.8172	O	-0.4387	-1.1726	2.2409	O	0.4633	-1.3215	-2.0835
O	4.4283	-1.2085	-0.7298	O	-4.9034	-2.1586	-1.4238	O	4.5828	-0.5383	0.3255

Table S10. Cartesian coordinates of the six lowest-energy conformers of the 3'*R*,5'*R* stereoisomer of pyrenosetins F at the B3LYP/TZVP/SMD($CHCl_3$)//B3LYP/6-31+G(d,p)/SMD($CHCl_3$) level.

Conformer 1				Conformer 2				Conformer 3			
C	0.3840	-1.4239	-0.9371	C	-0.4042	-1.3530	1.0723	C	0.3873	-1.4767	-0.8976
C	1.3948	-0.3426	-1.3064	C	-1.3941	-0.2305	1.3584	C	1.4035	-0.4127	-1.3008
C	0.5632	0.9701	-1.2263	C	-0.5318	1.0528	1.1978	C	0.5692	0.9005	-1.2950
C	1.3984	2.2245	-1.0563	C	-1.3376	2.3123	0.9431	C	1.4000	2.1632	-1.1744
C	2.6866	2.1595	-0.6895	C	-2.6235	2.2515	0.5679	C	2.6807	2.1190	-0.7791
C	3.4451	0.8914	-0.4054	C	-3.4064	0.9839	0.3555	C	3.4354	0.8675	-0.4200
C	4.3962	1.0243	0.8023	C	-4.3410	1.0590	-0.8699	C	4.3611	1.0582	0.7997
C	5.2373	-0.2469	1.0168	C	-5.2061	-0.2062	-1.0129	C	5.2010	-0.1988	1.0892
C	4.3263	-1.4838	1.1175	C	-4.3207	-1.4656	-1.0237	C	4.2923	-1.4341	1.2252
C	3.3516	-1.6032	-0.0659	C	-3.3612	-1.5291	0.1765	C	3.3423	-1.6094	0.0288
C	2.5135	-0.3205	-0.2039	C	-2.4990	-0.2570	0.2420	C	2.5031	-0.3372	-0.1818
C	1.9433	-0.6102	-2.7169	C	-1.9635	-0.3900	2.7761	C	1.9756	-0.7444	-2.6879
C	-0.4940	0.7152	-0.0929	C	0.5253	0.7107	0.0867	C	-0.5014	0.6973	-0.1630
C	-1.6902	1.6216	-0.0979	C	1.7537	1.5749	0.0928	C	-1.6886	1.6108	-0.2206
C	-2.1273	2.2923	0.9808	C	2.2771	2.1516	-1.0013	C	-2.1333	2.3428	0.8133
C	-3.3263	3.1630	1.0163	C	3.4979	2.9922	-1.0159	C	-3.3289	3.2216	0.6924
C	-4.1570	3.3465	-0.2376	C	4.2489	3.2475	0.2767	C	-3.7410	3.9664	1.9433
C	0.7360	3.5398	-1.3825	C	-0.6476	3.6311	1.1873	C	0.7438	3.4574	-1.5860
C	6.1562	-0.1186	2.2358	C	-6.1085	-0.1376	-2.2492	C	6.0923	-0.0127	2.3213
C	-1.2322	-1.5187	1.0086	C	1.0648	-1.6085	-0.9578	C	-1.2545	-1.4850	1.0260
C	-0.8706	-0.7781	-0.2675	C	0.8399	-0.7948	0.3102	C	-0.8766	-0.8022	-0.2762
C	-2.0593	-1.1033	-1.1906	C	2.0638	-1.1404	1.1697	C	-2.0536	-1.1696	-1.1996
C	-2.3236	-2.5401	0.6923	C	2.2180	-2.5859	-0.7238	C	-2.3339	-2.5273	0.7393
C	-3.4203	-2.5755	1.7669	C	3.2439	-2.5273	-1.8643	C	-3.4243	-2.5473	1.8197
C	-3.9071	-2.7309	-1.2984	C	3.8319	-2.8615	1.2309	C	-3.9085	-2.7925	-1.2506
H	-0.0041	1.0591	-2.1627	H	0.0330	1.1880	2.1307	H	0.0126	0.9434	-2.2407
H	3.2591	3.0852	-0.6224	H	-3.1747	3.1831	0.4350	H	3.2518	3.0476	-0.7483
H	4.0852	0.6852	-1.2801	H	-4.0607	0.8476	1.2335	H	4.0941	0.6234	-1.2709
H	5.0576	1.8879	0.6534	H	-4.9858	1.9436	-0.7850	H	5.0233	1.9166	0.6258
H	3.8067	1.2338	1.7068	H	-3.7370	1.1975	-1.7785	H	3.7523	1.3063	1.6815
H	5.8707	-0.3743	0.1250	H	-5.8522	-0.2630	-0.1228	H	5.8545	-0.3632	0.2183
H	4.9393	-2.3914	1.1858	H	-4.9522	-2.3629	-1.0417	H	4.9069	-2.3351	1.3458
H	2.7060	-2.4761	0.0725	H	-2.7316	-2.4214	0.1000	H	2.6967	-2.4780	0.1922
H	3.9202	-1.7773	-0.9885	H	-3.9425	-1.6339	1.1017	H	3.9301	-1.8214	-0.8736
H	2.0051	-0.1696	0.7597	H	-1.9769	-0.1795	-0.7225	H	1.9781	-0.1454	0.7655
H	0.0019	0.7973	0.8800	H	0.0468	0.7837	-0.8952	H	-0.0133	0.8186	0.8099
H	-1.5954	2.2262	1.9286	H	1.8076	2.0270	-1.9752	H	-1.6225	2.3180	1.7741
H	-2.2312	1.6905	-1.0386	H	2.2402	1.6984	1.0574	H	-2.2358	1.6609	-1.1598
H	-1.8608	-3.5384	0.6390	H	1.8129	-3.6073	-0.6584	H	-1.8587	-3.5213	0.7137
H	3.7511	-1.4268	2.0535	H	-3.7339	-1.4803	-1.9539	H	3.6979	-1.3390	2.1460
H	-4.1595	-3.3380	1.5049	H	4.0311	-3.2753	-1.6958	H	-4.1742	-3.3018	1.5654
H	-2.9495	-2.8800	2.7104	H	2.7270	-2.7893	-2.7916	H	-2.9496	-2.8562	2.7602
H	-3.5658	-0.7317	2.3852	H	4.3034	-1.0084	-1.2598	H	-3.5282	-0.6942	2.4088
H	2.4510	-1.5761	-2.7691	H	-2.4914	-1.3400	2.8871	H	2.4847	-1.7110	-2.6869
H	1.1265	-0.6232	-3.4457	H	-1.1553	-0.3706	3.5143	H	1.1711	-0.7918	-3.4288
H	2.6467	0.1710	-3.0167	H	-2.6541	0.4231	3.0145	H	2.6835	0.0231	-3.0116
H	-4.5354	2.3849	-0.6007	H	3.6152	3.7793	0.9950	H	-3.9421	3.2601	2.7580
H	-3.5575	3.7859	-1.0425	H	5.1316	3.8516	0.0611	H	-4.6314	4.5666	1.7494
H	-4.9976	4.0052	-0.0140	H	4.5567	2.3083	0.7487	H	-2.9250	4.6166	2.2819
H	-0.1158	3.7441	-0.7240	H	-1.3320	4.4708	1.0348	H	1.4393	4.2982	-1.5059
H	1.4398	4.3721	-1.2889	H	-0.2612	3.6891	2.2137	H	0.3915	3.4046	-2.6247
H	0.3447	3.5387	-2.4086	H	0.2116	3.7725	0.5217	H	-0.1354	3.6859	-0.9735
H	6.8272	0.7431	2.1422	H	-6.7611	0.7426	-2.2200	H	6.7138	-0.8976	2.5007
H	6.7783	-1.0124	2.3613	H	-6.7488	-1.0241	-2.3245	H	5.4900	0.1572	3.2225
H	5.5748	0.0122	3.1569	H	-5.5138	-0.0793	-3.1692	H	6.7628	0.8465	2.2040
H	-3.9511	-2.3441	-2.3166	H	3.9823	-2.4072	2.2103	H	-4.8507	-2.5555	-0.7466
H	-3.7775	-3.8179	-1.3286	H	3.5732	-3.9188	1.3568	H	-3.9552	-2.4307	-2.2778
H	-4.8457	-2.4957	-0.7863	H	4.7634	-2.7942	0.6597	H	-3.7660	-3.8786	-1.2543
N	-2.7785	-2.1092	-0.6277	N	2.7496	-2.1530	0.5667	N	-2.7897	-2.1393	-0.5937
O	0.4848	-2.6149	-1.1403	O	-0.5126	-2.5184	1.3886	O	0.4916	-2.6758	-1.0439
O	-3.6317	3.7227	2.0658	O	3.8848	3.4761	-2.0756	O	-3.9466	3.3406	-0.3601
O	-0.7722	-1.3145	2.1120	O	0.4413	-1.5072	-1.9906	O	-0.8182	-1.2236	2.1275
O	-2.2791	-0.5690	-2.2740	O	2.3433	-0.6201	2.2451	O	-2.2513	-0.6937	-2.3128
O	-4.1198	-1.3486	1.8850	O	3.7757	-1.2255	-2.0423	O	-4.1100	-1.3133	1.9444

Table S10 (continued)

Conformer 4				Conformer 5				Conformer 6			
C	0.4189	-1.4745	-0.9060	C	0.4557	-1.4889	-0.8865	C	0.3269	-1.1845	-1.2234
C	1.4232	-0.3973	-1.3035	C	1.4465	-0.4033	-1.2953	C	1.3514	-0.0712	-1.4045
C	0.5731	0.9051	-1.2911	C	0.5801	0.8882	-1.3008	C	0.5320	1.2196	-1.1214
C	1.3869	2.1776	-1.1594	C	1.3742	2.1736	-1.1725	C	1.3782	2.4211	-0.7464
C	2.6678	2.1472	-0.7636	C	2.6550	2.1644	-0.7756	C	2.6592	2.2816	-0.3754
C	3.4378	0.9027	-0.4127	C	3.4423	0.9332	-0.4166	C	3.3992	0.9744	-0.2853
C	4.3607	1.0975	0.8085	C	4.3638	1.1515	0.8017	C	4.3275	0.8980	0.9450
C	5.2153	-0.1511	1.0914	C	5.2348	-0.0830	1.0959	C	5.1522	-0.4016	0.9654
C	4.3209	-1.3976	1.2205	C	4.3564	-1.3392	1.2391	C	4.2287	-1.6278	0.8453
C	3.3739	-1.5776	0.0225	C	3.4108	-1.5435	0.0439	C	3.2757	-1.5404	-0.3583
C	2.5198	-0.3145	-0.1822	C	2.5405	-0.2937	-0.1737	C	2.4524	-0.2424	-0.2971
C	2.0012	-0.7148	-2.6912	C	2.0303	-0.7300	-2.6786	C	1.9178	-0.1140	-2.8317
C	-0.4988	0.6849	-0.1643	C	-0.4960	0.6688	-0.1808	C	-0.5353	0.8066	-0.0461
C	-1.7016	1.5775	-0.2325	C	-1.7178	1.5338	-0.2728	C	-1.7331	1.7073	0.0528
C	-2.1990	2.2602	0.8103	C	-2.2814	2.1595	0.7719	C	-2.2164	2.1896	1.2092
C	-3.4171	3.1044	0.6820	C	-3.5335	2.9510	0.6288	C	-3.4177	3.0485	1.3390
C	-3.9095	3.7632	1.9522	C	-4.1109	3.5188	1.9060	C	-4.1819	3.4628	0.0973
C	0.7114	3.4669	-1.5548	C	0.6763	3.4514	-1.5659	C	0.7330	3.7792	-0.8656
C	6.1043	0.0390	2.3245	C	6.1228	0.1310	2.3258	C	6.0456	-0.4850	2.2071
C	-1.1974	-1.5114	1.0334	C	-1.1710	-1.5071	1.0585	C	-1.2078	-1.5736	0.7432
C	-0.8503	-0.8223	-0.2772	C	-0.8254	-0.8479	-0.2692	C	-0.9086	-0.6520	-0.4243
C	-2.0296	-1.1993	-1.1894	C	-1.9943	-1.2524	-1.1795	C	-2.1314	-0.8531	-1.3365
C	-2.3368	-2.5026	0.7827	C	-2.3787	-2.4256	0.8647	C	-2.3464	-2.5105	0.3609
C	-3.4310	-2.3991	1.8416	C	-3.4742	-2.1424	1.9035	C	-3.3763	-2.6214	1.5062
C	-3.9179	-2.7829	-1.1959	C	-3.8590	-2.8696	-1.1635	C	-3.9775	-2.4702	-1.5994
H	0.0187	0.9472	-2.2384	H	0.0333	0.9151	-2.2531	H	-0.0267	1.4625	-2.0359
H	3.2261	3.0833	-0.7231	H	3.1982	3.1094	-0.7366	H	3.2398	3.1773	-0.1520
H	4.0997	0.6716	-1.2648	H	4.1061	0.7033	-1.2675	H	4.0545	0.9034	-1.1701
H	5.0126	1.9647	0.6398	H	5.0042	2.0256	0.6238	H	4.9998	1.7660	0.9505
H	3.7484	1.3332	1.6914	H	3.7494	1.3878	1.6829	H	3.7219	0.9652	1.8606
H	5.8708	-0.3031	0.2196	H	5.8911	-0.2355	0.2249	H	5.8037	-0.3897	0.0776
H	4.9460	-2.2919	1.3370	H	4.9928	-2.2244	1.3640	H	4.8325	-2.5412	0.7748
H	2.7381	-2.4542	0.1811	H	2.7863	-2.4263	0.2123	H	2.6198	-2.4166	-0.3753
H	3.9648	-1.7783	-0.8805	H	4.0037	-1.7461	-0.8574	H	3.8594	-1.5677	-1.2874
H	1.9903	-0.1354	0.7649	H	2.0074	-0.1120	0.7709	H	1.9305	-0.2454	0.6708
H	-0.0188	0.8143	0.8116	H	-0.0273	0.8210	0.7974	H	-0.0513	0.7576	0.9345
H	-1.7253	2.2064	1.7884	H	-1.8469	2.0844	1.7663	H	-1.7271	1.9606	2.1544
H	-2.2193	1.6461	-1.1870	H	-2.1983	1.6198	-1.2457	H	-2.2355	1.9340	-0.8845
H	-1.9235	-3.5225	0.8141	H	-2.0535	-3.4722	0.9674	H	-1.9399	-3.5139	0.1647
H	3.7246	-1.3140	2.1411	H	3.7599	-1.2536	2.1593	H	3.6358	-1.7184	1.7674
H	-4.1853	-3.1810	1.6731	H	-4.3050	-2.8486	1.7669	H	-3.7342	-1.6146	1.7705
H	-2.9593	-2.5913	2.8153	H	-3.0478	-2.3153	2.8955	H	-4.2373	-3.2052	1.1683
H	-4.6254	-1.0037	2.4904	H	-4.3402	-0.6383	1.0173	H	-2.0433	-2.7947	2.8917
H	1.1983	-0.7681	-3.4335	H	2.7192	0.0530	-3.0066	H	2.4190	-1.0637	-3.0318
H	2.7006	0.0624	-3.0105	H	2.5640	-1.6832	-2.6698	H	1.1116	-0.0018	-3.5637
H	2.5215	-1.6755	-2.6945	H	1.2289	-0.8034	-3.4207	H	2.6312	0.6984	-2.9927
H	-4.1196	3.0042	2.7154	H	-5.0222	4.0804	1.6941	H	-4.5302	2.5872	-0.4610
H	-4.8112	4.3444	1.7521	H	-3.3791	4.1716	2.3970	H	-3.5434	4.0453	-0.5760
H	-3.1322	4.4190	2.3633	H	-4.3313	2.7054	2.6084	H	-5.0398	4.0685	0.3933
H	1.3958	4.3163	-1.4694	H	1.3484	4.3112	-1.4870	H	-0.1268	3.8842	-0.1941
H	0.3539	3.4203	-2.5919	H	0.3109	3.3980	-2.6000	H	1.4426	4.5777	-0.6296
H	-0.1674	3.6768	-0.9347	H	-0.2002	3.6498	-0.9383	H	0.3571	3.9480	-1.8836
H	6.7646	0.9067	2.2119	H	5.5177	0.2899	3.2270	H	6.7264	0.3720	2.2685
H	6.7362	-0.8394	2.4993	H	6.7716	1.0061	2.2036	H	6.6565	-1.3951	2.1979
H	5.5000	0.1969	3.2265	H	6.7664	-0.7373	2.5083	H	5.4454	-0.4982	3.1254
H	-4.8450	-2.4788	-0.6991	H	-3.6663	-3.9474	-1.1205	H	-3.8027	-3.5347	-1.7888
H	-3.9518	-2.4611	-2.2369	H	-4.8260	-2.6657	-0.6921	H	-4.9159	-2.3565	-1.0481
H	-3.8282	-3.8736	-1.1557	H	-3.8987	-2.5499	-2.2049	H	-4.0586	-1.9422	-2.5497
N	-2.7668	-2.1587	-0.5691	N	-2.7853	-2.1390	-0.5089	N	-2.8593	-1.9020	-0.8627
O	0.5375	-2.6718	-1.0595	O	0.5896	-2.6861	-1.0246	O	0.4013	-2.3207	-1.6377
O	-3.9939	3.2623	-0.3887	O	-4.0757	3.1244	-0.4580	O	-3.7778	3.4136	2.4545
O	-0.6695	-1.3163	2.1058	O	-0.5972	-1.3321	2.1105	O	-0.6708	-1.5447	1.8348
O	-2.2296	-0.7412	-2.3102	O	-2.1546	-0.8737	-2.3347	O	-2.3741	-0.1983	-2.3448
O	-3.9929	-1.0978	1.7642	O	-3.9126	-0.7942	1.8723	O	-2.8216	-3.3049	2.6127

Table S11. Cartesian coordinates of the six lowest-energy conformers of the 3'*S*,5'*R* stereoisomer of pyrenosetins F at the B3LYP/TZVP/SMD($CHCl_3$)//B3LYP/6-31+G(d,p)/SMD($CHCl_3$) level

Conformer 1				Conformer 2				Conformer 3			
C	0.1944	-1.3147	-0.8536	C	0.1452	-1.3537	-0.8089	C	0.1868	-1.1975	-1.0020
C	1.2177	-0.2857	-1.3039	C	1.2492	-0.4178	-1.2878	C	1.1858	-0.1129	-1.3612
C	0.4053	1.0420	-1.2917	C	0.5597	0.9767	-1.2901	C	0.3512	1.1905	-1.2061
C	1.2616	2.2913	-1.2147	C	1.5227	2.1470	-1.2367	C	1.1890	2.4416	-1.0220
C	2.5512	2.2287	-0.8523	C	2.8058	1.9761	-0.8866	C	2.4842	2.3662	-0.6834
C	3.2929	0.9687	-0.4955	C	3.4372	0.6604	-0.5193	C	3.2488	1.0905	-0.4534
C	4.2555	1.1589	0.6954	C	4.4263	0.7821	0.6586	C	4.2178	1.1829	0.7439
C	5.0804	-0.1098	0.9775	C	5.1409	-0.5495	0.9512	C	5.0638	-0.0939	0.8991
C	4.1538	-1.3261	1.1575	C	4.1135	-1.6773	1.1576	C	4.1574	-1.3365	0.9695
C	3.1667	-1.5010	-0.0085	C	3.1018	-1.7806	0.0040	C	3.1631	-1.4163	-0.2012
C	2.3473	-0.2153	-0.2124	C	2.3937	-0.4318	-0.2110	C	2.3239	-0.1296	-0.2759
C	1.7495	-0.6443	-2.6997	C	1.7295	-0.8455	-2.6830	C	1.7093	-0.3211	-2.7894
C	-0.6253	0.8805	-0.1204	C	-0.4762	0.9210	-0.1128	C	-0.6577	0.9049	-0.0384
C	-1.8146	1.7928	-0.1694	C	-1.5635	1.9527	-0.1523	C	-1.8851	1.7710	-0.0315
C	-2.2647	2.5057	0.8748	C	-1.9242	2.7128	0.8935	C	-2.3690	2.3798	1.0632
C	-3.4710	3.3723	0.7681	C	-3.0304	3.7042	0.7947	C	-3.5967	3.2105	1.1041
C	-3.8748	4.1127	2.0232	C	-3.3263	4.5018	2.0452	C	-4.3944	3.4346	-0.1652
C	0.6135	3.5914	-1.6197	C	0.9891	3.4943	-1.6547	C	0.5142	3.7642	-1.2860
C	6.0107	0.0763	2.1805	C	6.0968	-0.4284	2.1423	C	6.0002	-0.0072	2.1085
C	-2.2546	-1.0306	-0.9470	C	-2.2684	-0.8074	-0.9782	C	-2.2814	-1.0189	-0.8561
C	-1.0056	-0.6293	-0.1539	C	-1.0088	-0.5387	-0.1516	C	-0.9775	-0.6173	-0.1596
C	-1.2788	-1.2221	1.2378	C	-1.3699	-1.1119	1.2226	C	-1.0871	-1.3270	1.2023
C	-3.0822	-2.0005	-0.1058	C	-3.1828	-1.7415	-0.1878	C	-2.9791	-2.0915	-0.0218
C	-3.1467	-3.4294	-0.7128	C	-3.3282	-3.1039	-0.9000	C	-3.0513	-3.4705	-0.7343
C	-2.9356	-2.6849	2.3369	C	-3.1805	-2.3913	2.2903	C	-2.5595	-2.9533	2.3322
H	-0.1783	1.0817	-2.2223	H	-0.0250	1.0561	-2.2172	H	-0.2481	1.3087	-2.1205
H	3.1386	3.1476	-0.8475	H	3.4718	2.8396	-0.9010	H	3.0569	3.2902	-0.5970
H	3.9229	0.7009	-1.3608	H	4.0314	0.3262	-1.3868	H	3.8759	0.9157	-1.3443
H	4.9276	2.0028	0.4909	H	5.1669	1.5614	0.4356	H	4.8757	2.0526	0.6157
H	3.6761	1.4290	1.5903	H	3.8828	1.1137	1.5554	H	3.6414	1.3581	1.6639
H	5.7051	-0.2976	0.0902	H	5.7371	-0.8033	0.0608	H	5.6843	-0.1869	-0.0060
H	4.7553	-2.2366	1.2731	H	4.6343	-2.6353	1.2810	H	4.7736	-2.2440	0.9948
H	2.5091	-2.3532	0.1906	H	2.3753	-2.5701	0.2193	H	2.5195	-2.2933	-0.0771
H	3.7231	-1.7396	-0.9241	H	3.6259	-2.0772	-0.9139	H	3.7142	-1.5598	-1.1395
H	1.8510	-0.0053	0.7452	H	1.9277	-0.1657	0.7484	H	1.8331	-0.0200	0.7007
H	-0.1067	1.0310	0.8316	H	0.0595	1.0136	0.8374	H	-0.1386	1.0242	0.9172
H	-1.7542	2.4688	1.8351	H	-1.4100	2.6256	1.8485	H	-1.8611	2.2855	2.0214
H	-2.3611	1.8609	-1.1085	H	-2.1105	2.0754	-1.0855	H	-2.4061	1.8718	-0.9808
H	-4.1089	-1.6112	-0.0563	H	-4.1820	-1.2912	-0.1125	H	-4.0105	-1.7571	0.1606
H	3.5871	-1.2076	2.0926	H	3.5699	-1.4964	2.0966	H	3.5957	-1.3155	1.9147
H	-3.9866	-3.9638	-0.2587	H	-3.6924	-2.9036	-1.9129	H	-3.8161	-4.0779	-0.2410
H	-3.3705	-3.3166	-1.7850	H	-2.3458	-3.5839	-0.9738	H	-3.3930	-3.2854	-1.7646
H	-1.2081	-3.7194	-0.7718	H	-3.8227	-4.4645	0.4068	H	-1.1243	-3.6611	-1.0271
H	0.9269	-0.6895	-3.4207	H	0.9001	-0.8153	-3.3971	H	0.8807	-0.3096	-3.5048
H	2.4598	0.1088	-3.0502	H	2.5109	-0.1750	-3.0502	H	2.4036	0.4752	-3.0692
H	2.2450	-1.6177	-2.6992	H	2.1218	-1.8651	-2.6719	H	2.2210	-1.2809	-2.8898
H	-4.0617	3.4029	2.8382	H	-2.4370	5.0663	2.3514	H	-3.7856	3.9376	-0.9245
H	-4.7710	4.7077	1.8401	H	-3.5744	3.8286	2.8748	H	-5.2638	4.0520	0.0659
H	-3.0589	4.7665	2.3548	H	-4.1548	5.1895	1.8687	H	-4.7284	2.4833	-0.5932
H	0.2271	3.5346	-2.6460	H	1.7732	4.2570	-1.6284	H	0.0988	3.7990	-2.3020
H	-0.2414	3.8405	-0.9808	H	0.5844	3.4572	-2.6747	H	-0.3223	3.9404	-0.5999
H	1.3243	4.4215	-1.5703	H	0.1699	3.8304	-1.0090	H	1.2167	4.5959	-1.1785
H	5.4382	0.2686	3.0963	H	6.8478	0.3524	1.9750	H	6.6264	-0.9030	2.1922
H	6.6929	0.9214	2.0319	H	6.6296	-1.3693	2.3226	H	5.4316	0.0878	3.0418
H	6.6211	-0.8176	2.3535	H	5.5531	-0.1745	3.0607	H	6.6674	0.8596	2.0368
H	-2.3542	-2.3979	3.2132	H	-4.2594	-2.2219	2.2500	H	-3.6026	-2.7815	2.6170
H	-3.9905	-2.4451	2.5058	H	-2.9933	-3.4691	2.3491	H	-2.4233	-4.0082	2.0746
H	-2.8316	-3.7618	2.1719	H	-2.7686	-1.9229	3.1857	H	-1.9097	-2.7009	3.1702
N	-2.4301	-1.9441	1.1942	N	-2.5453	-1.7923	1.1259	N	-2.2017	-2.1047	1.2085
O	0.2556	-2.5163	-1.0533	O	0.1181	-2.5613	-0.9276	O	0.2431	-2.3639	-1.3538
O	-4.1039	3.4802	-0.2767	O	-3.6667	3.8692	-0.2407	O	-3.9521	3.7050	2.1698
O	-2.5372	-0.6775	-2.0709	O	-2.5043	-0.3909	-2.0919	O	-2.6979	-0.5854	-1.9083
O	-0.5694	-1.0427	2.2217	O	-0.7016	-0.9677	2.2408	O	-0.2925	-1.1872	2.1255
O	-1.9971	-4.2024	-0.4640	O	-4.2824	-3.9420	-0.2646	O	-1.8519	-4.2035	-0.6707

Table S11 (continued)

Conformer 4				Conformer 5				Conformer 6			
C	0.1392	-1.2804	-0.9006	C	0.1949	-1.4464	-0.6662	C	0.1437	-1.3468	-0.8341
C	1.2370	-0.3109	-1.3206	C	1.2363	-0.4954	-1.2443	C	1.2501	-0.4083	-1.3011
C	0.5427	1.0770	-1.2198	C	0.4840	0.8648	-1.3044	C	0.5620	0.9866	-1.2915
C	1.5042	2.2441	-1.1013	C	1.3935	2.0772	-1.3616	C	1.5273	2.1547	-1.2291
C	2.7914	2.0537	-0.7779	C	2.6953	1.9842	-1.0539	C	2.8091	1.9788	-0.8768
C	3.4285	0.7190	-0.5012	C	3.3999	0.7228	-0.6335	C	3.4371	0.6595	-0.5168
C	4.4258	0.7674	0.6752	C	4.4269	0.9611	0.4932	C	4.4217	0.7702	0.6659
C	5.1442	-0.5789	0.8770	C	5.2122	-0.3172	0.8374	C	5.1329	-0.5650	0.9505
C	4.1204	-1.7200	1.0181	C	4.2465	-1.4739	1.1530	C	4.1031	-1.6930	1.1438
C	3.1002	-1.7516	-0.1325	C	3.1977	-1.6932	0.0499	C	3.0955	-1.7853	-0.0145
C	2.3895	-0.3929	-0.2552	C	2.4207	-0.3923	-0.2165	C	2.3908	-0.4334	-0.2208
C	1.7090	-0.6391	-2.7450	C	1.6816	-0.9880	-2.6296	C	1.7341	-0.8234	-2.6987
C	-0.4792	0.9411	-0.0362	C	-0.5028	0.8420	-0.0848	C	-0.4722	0.9235	-0.1130
C	-1.5809	1.9618	-0.0158	C	-1.6408	1.8161	-0.1507	C	-1.5608	1.9541	-0.1484
C	-1.9465	2.6474	1.0797	C	-2.0049	2.6278	0.8540	C	-1.9213	2.7115	0.8993
C	-3.0473	3.6384	1.1396	C	-3.1642	3.5541	0.7286	C	-3.0280	3.7026	0.8033
C	-3.8365	3.9613	-0.1136	C	-3.4644	4.4167	1.9337	C	-3.3247	4.4962	2.0561
C	0.9640	3.6157	-1.4205	C	0.7830	3.3711	-1.8388	C	0.9972	3.5057	-1.6395
C	6.1079	-0.5322	2.0671	C	6.2050	-0.0806	1.9800	C	6.0843	-0.4551	2.1463
C	-2.2884	-0.7536	-0.9262	C	-2.2491	-1.0084	-0.7496	C	-2.2705	-0.8061	-0.9623
C	-0.9945	-0.5217	-0.1461	C	-0.9680	-0.6416	-0.0066	C	-1.0022	-0.5372	-0.1540
C	-1.2795	-1.1967	1.2009	C	-1.2401	-1.1330	1.4195	C	-1.3377	-1.1224	1.2224
C	-3.1532	-1.7524	-0.1602	C	-3.1531	-1.8101	0.1775	C	-3.1743	-1.7425	-0.1580
C	-3.3414	-3.0587	-0.9604	C	-3.4195	-3.2139	-0.4218	C	-3.3523	-3.0854	-0.8753
C	-2.9981	-2.6153	2.2461	C	-2.9912	-2.3567	2.6587	C	-3.1193	-2.4466	2.2977
H	-0.0533	1.2153	-2.1332	H	-0.1382	0.8603	-2.2104	H	-0.0234	1.0743	-2.2175
H	3.4554	2.9182	-0.7429	H	3.3201	2.8731	-1.1477	H	3.4768	2.8412	-0.8836
H	4.0172	0.4428	-1.3926	H	3.9751	0.3616	-1.5031	H	4.0343	0.3309	-1.3845
H	5.1638	1.5607	0.4972	H	5.1221	1.7562	0.1927	H	5.1646	1.5500	0.4521
H	3.8880	1.0401	1.5949	H	3.9034	1.3248	1.3895	H	3.8752	1.0955	1.5632
H	5.7349	-0.7741	-0.0318	H	5.7862	-0.5998	-0.0590	H	5.7323	-0.8125	0.0604
H	4.6440	-2.6826	1.0761	H	4.8152	-2.3986	1.3128	H	4.6220	-2.6526	1.2613
H	2.3758	-2.5540	0.0387	H	2.5169	-2.4985	0.3426	H	2.3665	-2.5750	0.1924
H	3.6173	-1.9891	-1.0713	H	3.6994	-2.0233	-0.8689	H	3.6222	-2.0758	-0.9327
H	1.9315	-0.1898	0.7229	H	1.9810	-0.0883	0.7438	H	1.9221	-0.1749	0.7392
H	0.0634	0.9941	0.9127	H	0.0618	1.0225	0.8354	H	0.0647	1.0141	0.8365
H	-1.4279	2.4979	2.0252	H	-1.4551	2.6328	1.7930	H	-1.4066	2.6223	1.8540
H	-2.1116	2.1152	-0.9528	H	-2.2246	1.8494	-1.0696	H	-2.1086	2.0789	-1.0809
H	-4.1479	-1.3142	0.0019	H	-4.1210	-1.2991	0.2797	H	-4.1660	-1.2788	-0.0554
H	3.5832	-1.6009	1.9704	H	3.7308	-1.2592	2.1005	H	3.5561	-1.5190	2.0820
H	-3.7373	-2.7839	-1.9435	H	-2.4583	-3.7006	-0.6412	H	-3.6552	-2.8723	-1.9108
H	-2.3691	-3.5443	-1.1021	H	-3.9476	-3.8253	0.3158	H	-2.3903	-3.6131	-0.8953
H	-3.8201	-4.4853	0.2816	H	-3.8201	-2.5325	-2.1923	H	-4.3573	-4.7206	-0.5465
H	0.8733	-0.5698	-3.4491	H	2.4139	-0.3063	-3.0701	H	2.1255	-1.8434	-2.6958
H	2.4808	0.0621	-3.0730	H	2.1237	-1.9854	-2.5726	H	0.9069	-0.7860	-3.4150
H	2.1108	-1.6531	-2.8057	H	0.8242	-1.0419	-3.3084	H	2.5171	-0.1502	-3.0576
H	-3.1827	4.3764	-0.8886	H	-2.5932	5.0347	2.1830	H	-2.4357	5.0600	2.3645
H	-4.6105	4.6901	0.1317	H	-3.6657	3.7868	2.8088	H	-3.5730	3.8204	2.8835
H	-4.3037	3.0617	-0.5285	H	-4.3254	5.0571	1.7359	H	-4.1533	5.1843	1.8812
H	0.1632	3.9107	-0.7328	H	0.3406	3.2522	-2.8367	H	0.1757	3.8380	-0.9947
H	1.7503	4.3742	-1.3641	H	-0.0246	3.7122	-1.1811	H	1.7820	4.2672	-1.6046
H	0.5354	3.6446	-2.4312	H	1.5319	4.1668	-1.8926	H	0.5965	3.4765	-2.6615
H	6.6435	-1.4815	2.1835	H	6.7869	-0.9842	2.1955	H	6.8372	0.3258	1.9884
H	5.5697	-0.3386	3.0033	H	5.6844	0.2057	2.9023	H	6.6148	-1.3983	2.3211
H	6.8564	0.2593	1.9452	H	6.9125	0.7205	1.7362	H	5.5372	-0.2078	3.0645
H	-2.7836	-3.6889	2.1920	H	-3.0638	-3.4476	2.6114	H	-4.1216	-2.0366	2.4632
H	-2.5443	-2.2210	3.1566	H	-2.3386	-2.0803	3.4871	H	-3.2063	-3.5254	2.1536
H	-4.0808	-2.4709	2.2834	H	-3.9908	-1.9418	2.8284	H	-2.4924	-2.2366	3.1646
N	-2.4445	-1.8956	1.1088	N	-2.4221	-1.8117	1.4368	N	-2.5062	-1.8153	1.1383
O	0.1016	-2.4728	-1.1232	O	0.2115	-2.6578	-0.7147	O	0.1077	-2.5514	-0.9769
O	-3.3042	4.1876	2.2070	O	-3.8396	3.6154	-0.2932	O	-3.6641	3.8704	-0.2318
O	-2.5874	-0.2593	-1.9926	O	-2.4971	-0.7784	-1.9187	O	-2.5250	-0.3859	-2.0701
O	-0.5628	-1.1157	2.1923	O	-0.5038	-0.9361	2.3794	O	-0.6489	-0.9778	2.2272
O	-4.2878	-3.9257	-0.3533	O	-4.2567	-3.1238	-1.5576	O	-4.3508	-3.8240	-0.1817

Table S12. Cartesian coordinates of the six lowest-energy conformers of the 3'*R*,5'*S* stereoisomer of pyrenosetins F at the B3LYP/TZVP/SMD($CHCl_3$)//B3LYP/6-31+G(d,p)/SMD($CHCl_3$) level

Conformer 1				Conformer 2				Conformer 3			
C	-0.1945	-1.3040	0.7829	C	-0.1884	-1.3781	0.6661	C	-0.1610	-1.3171	0.8571
C	-1.1852	-0.2585	1.2691	C	-1.1945	-0.3791	1.2186	C	-1.2436	-0.3376	1.2977
C	-0.3720	1.0659	1.1848	C	-0.3938	0.9563	1.2396	C	-0.5273	1.0416	1.2296
C	-1.2254	2.3174	1.1209	C	-1.2569	2.2022	1.2549	C	-1.4683	2.2265	1.1313
C	-2.5343	2.2528	0.8377	C	-2.5648	2.1467	0.9652	C	-2.7587	2.0649	0.8044
C	-3.2994	0.9889	0.5539	C	-3.3190	0.8987	0.5948	C	-3.4201	0.7466	0.5059
C	-4.3291	1.1610	-0.5825	C	-4.3550	1.1425	-0.5225	C	-4.4217	0.8333	-0.6645
C	-5.1750	-0.1074	-0.7932	C	-5.1894	-0.1154	-0.8217	C	-5.1651	-0.4960	-0.8871
C	-4.2651	-1.3316	-1.0011	C	-4.2677	-1.3101	-1.1249	C	-4.1623	-1.6521	-1.0534
C	-3.2141	-1.4891	0.1103	C	-3.2108	-1.5394	-0.0315	C	-3.1378	-1.7220	0.0915
C	-2.3759	-0.2059	0.2421	C	-2.3846	-0.2616	0.1963	C	-2.4015	-0.3790	0.2366
C	-1.6368	-0.5901	2.7002	C	-1.6457	-0.8197	2.6210	C	-1.7145	-0.6908	2.7173
C	0.6066	0.8664	-0.0269	C	0.6026	0.8458	0.0310	C	0.4988	0.9078	0.0481
C	1.7911	1.7878	-0.0643	C	1.7613	1.7964	0.0447	C	1.6034	1.9209	0.0237
C	2.1706	2.4779	-1.1519	C	2.1180	2.5693	-0.9928	C	1.9359	2.6520	-1.0518
C	3.3568	3.3640	-1.2282	C	3.2973	3.4758	-0.9246	C	3.0619	3.6250	-1.0199
C	4.2336	3.5501	-0.0062	C	3.5843	4.2929	-2.1647	C	3.3245	4.3844	-2.3016
C	-0.5525	3.6255	1.4541	C	-0.5960	3.4883	1.6838	C	-0.9061	3.5806	1.4854
C	-6.1700	0.0612	-1.9458	C	-6.1943	0.1273	-1.9522	C	-6.1337	-0.4110	-2.0711
C	1.2923	-1.3282	-1.2546	C	1.4293	-1.2475	-1.2782	C	1.3994	-1.2163	-1.1450
C	1.0086	-0.6340	0.0741	C	1.0380	-0.6468	0.0672	C	1.0120	-0.5537	0.1715
C	2.2786	-0.9310	0.8958	C	2.2598	-0.9645	0.9544	C	2.2579	-0.7714	1.0466
C	2.5547	-2.1755	-1.1180	C	2.7012	-2.0739	-1.1085	C	2.6766	-2.0259	-0.9388
C	2.2839	-3.7010	-1.2293	C	2.4735	-3.5938	-1.3309	C	2.4046	-3.5367	-1.1057
C	4.3228	-2.2980	0.7048	C	4.3495	-2.2666	0.8181	C	4.3985	-1.9784	0.9594
H	0.2584	1.1230	2.0824	H	0.2253	0.9557	2.1461	H	0.0699	1.1504	2.1447
H	-3.1177	3.1741	0.8451	H	-3.1562	3.0602	1.0357	H	-3.4083	2.9409	0.7883
H	-3.8781	0.7423	1.4603	H	-3.8922	0.5820	1.4827	H	-4.0094	0.4644	1.3950
H	-4.9840	2.0125	-0.3553	H	-5.0167	1.9690	-0.2319	H	-5.1445	1.6363	-0.4689
H	-3.8015	1.4118	-1.5143	H	-3.8339	1.4652	-1.4359	H	-3.8836	1.1131	-1.5821
H	-5.7497	-0.2750	0.1310	H	-5.7560	-0.3574	0.0911	H	-5.7546	-0.6971	0.0211
H	-4.8754	-2.2412	-1.0647	H	-4.8683	-2.2191	-1.2548	H	-4.7028	-2.6043	-1.1260
H	-2.5754	-2.3507	-0.1063	H	-2.5640	-2.3757	-0.3133	H	-2.4293	-2.5350	-0.0950
H	-3.7190	-1.7037	1.0610	H	-3.7110	-1.8301	0.9012	H	-3.6556	-1.9658	1.0281
H	-1.9331	-0.0148	-0.7460	H	-1.9434	0.0081	-0.7743	H	-1.9444	-0.1638	-0.7400
H	0.0459	0.9756	-0.9609	H	0.0465	0.9811	-0.9030	H	-0.0460	0.9602	-0.9004
H	1.5999	2.4153	-2.0769	H	1.5466	2.5570	-1.9190	H	1.3833	2.5541	-1.9843
H	2.3722	1.8523	0.8526	H	2.3649	1.8377	0.9497	H	2.1875	2.0469	0.9333
H	3.2405	-1.8984	-1.9309	H	3.4325	-1.7327	-1.8547	H	3.4236	-1.7308	-1.6879
H	-3.7523	-1.2338	-1.9694	H	-3.7604	-1.1339	-2.0850	H	-3.6282	-1.5255	-2.0068
H	1.6424	-3.8519	-2.1114	H	1.9041	-3.7015	-2.2673	H	1.6584	-3.8567	-0.3698
H	3.2320	-4.2113	-1.4245	H	3.4446	-4.0748	-1.4824	H	1.9861	-3.6821	-2.1071
H	0.9504	-3.7865	0.2064	H	1.0326	-3.7942	-0.0158	H	3.7292	-4.5532	-0.1004
H	-0.7740	-0.6215	3.3730	H	-2.1366	-1.7952	2.5933	H	-2.1344	-1.6986	2.7568
H	-2.3271	0.1699	3.0755	H	-0.7827	-0.8972	3.2897	H	-0.8733	-0.6518	3.4167
H	-2.1314	-1.5630	2.7454	H	-2.3386	-0.0920	3.0515	H	-2.4718	0.0169	3.0650
H	3.6624	3.9896	0.8190	H	4.4647	4.9187	-2.0106	H	3.5325	3.6853	-3.1208
H	5.0633	4.2113	-0.2611	H	2.7210	4.9236	-2.4100	H	4.1695	5.0628	-2.1735
H	4.6280	2.5909	0.3458	H	3.7468	3.6314	-3.0244	H	2.4338	4.9552	-2.5914
H	0.2536	3.8627	0.7506	H	0.2194	3.7766	1.0110	H	-0.0840	3.8711	0.8217
H	-1.2668	4.4539	1.4373	H	-1.3150	4.3123	1.7145	H	-1.6754	4.3566	1.4294
H	-0.0963	3.5897	2.4523	H	-0.1517	3.3864	2.6828	H	-0.4961	3.5803	2.5040
H	-5.6487	0.2325	-2.8958	H	-6.8703	0.9571	-1.7154	H	-6.8677	0.3911	-1.9308
H	-6.8383	0.9133	-1.7759	H	-6.8097	-0.7611	-2.1355	H	-6.6863	-1.3486	-2.2018
H	-6.7939	-0.8319	-2.0669	H	-5.6817	0.3744	-2.8902	H	-5.5971	-0.2097	-3.0066
H	4.5617	-1.7634	1.6242	H	5.1955	-2.0652	0.1531	H	4.6734	-1.2273	1.7013
H	4.2204	-3.3651	0.9245	H	4.5181	-1.7750	1.7764	H	4.3710	-2.9565	1.4521
H	5.1299	-2.1534	-0.0206	H	4.2618	-3.3460	0.9764	H	5.1495	-2.0035	0.1661
N	3.0774	-1.7651	0.1789	N	3.1259	-1.7342	0.2431	N	3.1047	-1.6174	0.3979
O	-0.3027	-2.5106	0.9208	O	-0.3000	-2.5921	0.6882	O	-0.1615	-2.5190	1.0247
O	3.6134	3.9350	-2.2842	O	3.9985	3.5574	0.0780	O	3.7403	3.8043	-0.0141
O	0.6242	-1.2344	-2.2605	O	0.8319	-1.0994	-2.3218	O	0.7942	-1.1286	-2.1916
O	2.5146	-0.4804	2.0129	O	2.4073	-0.5837	2.1112	O	2.4425	-0.2774	2.1545
O	1.7553	-4.2703	-0.0553	O	1.8698	-4.2422	-0.2371	O	3.5946	-4.3079	-1.0259

Table S12 (continued)

Conformer 4				Conformer 5				Conformer 6			
C	-0.1668	-1.2380	0.9643	C	-0.2233	-1.1760	1.1037	C	-0.1690	-1.2343	0.9651
C	-1.2421	-0.2229	1.3324	C	-1.2237	-0.0613	1.3842	C	-1.2441	-0.2180	1.3316
C	-0.5162	1.1420	1.1632	C	-0.3997	1.2317	1.1269	C	-0.5174	1.1464	1.1614
C	-1.4509	2.3233	0.9875	C	-1.2442	2.4610	0.8514	C	-1.4510	2.3280	0.9820
C	-2.7429	2.1469	0.6747	C	-2.5426	2.3551	0.5333	C	-2.7429	2.1520	0.6687
C	-3.4109	0.8150	0.4647	C	-3.3039	1.0627	0.4128	C	-3.4118	0.8201	0.4615
C	-4.4101	0.8281	-0.7111	C	-4.2907	1.0597	-0.7736	C	-4.4111	0.8318	-0.7143
C	-5.1588	-0.5100	-0.8460	C	-5.1339	-0.2273	-0.8184	C	-5.1605	-0.5061	-0.8471
C	-4.1610	-1.6790	-0.9334	C	-4.2233	-1.4686	-0.8053	C	-4.1633	-1.6758	-0.9326
C	-3.1392	-1.6776	0.2161	C	-3.2130	-1.4543	0.3541	C	-3.1416	-1.6731	0.2169
C	-2.3976	-0.3310	0.2731	C	-2.3755	-0.1644	0.3200	C	-2.3993	-0.3269	0.2721
C	-1.7188	-0.4686	2.7719	C	-1.7275	-0.1663	2.8316	C	-1.7214	-0.4620	2.7712
C	0.5010	0.9208	-0.0129	C	0.6100	0.8549	-0.0158	C	0.5015	0.9235	-0.0126
C	1.6231	1.9150	-0.0949	C	1.8016	1.7586	-0.1504	C	1.6253	1.9159	-0.0932
C	1.9669	2.5639	-1.2194	C	2.2052	2.2985	-1.3119	C	1.9799	2.5546	-1.2203
C	3.0884	3.5251	-1.3420	C	3.3925	3.1699	-1.4822	C	3.1058	3.5105	-1.3423
C	3.9366	3.8481	-0.1283	C	4.2398	3.5280	-0.2779	C	3.9397	3.8478	-0.1226
C	-0.8805	3.6959	1.2433	C	-0.5773	3.8041	1.0145	C	-0.8792	3.7008	1.2335
C	-6.1251	-0.5000	-2.0348	C	-6.0875	-0.2373	-2.0175	C	-6.1269	-0.4975	-2.0359
C	1.3045	-1.3016	-1.0914	C	1.2749	-1.4893	-0.9137	C	1.3048	-1.2982	-1.0905
C	0.9929	-0.5404	0.1896	C	0.9952	-0.6201	0.3010	C	0.9918	-0.5380	0.1911
C	2.2728	-0.7232	1.0214	C	2.2463	-0.8246	1.1709	C	2.2715	-0.7218	1.0233
C	2.5853	-2.1115	-0.8906	C	2.5447	-2.2945	-0.6798	C	2.5800	-2.1130	-0.8866
C	2.3007	-3.6161	-0.9586	C	2.2276	-3.8103	-0.7146	C	2.2866	-3.6280	-0.9436
C	4.3829	-1.9939	0.9237	C	4.2895	-2.2098	1.1742	C	4.3760	-2.0040	0.9284
H	0.0870	1.3098	2.0660	H	0.2057	1.4221	2.0237	H	0.0842	1.3154	2.0650
H	-3.3875	3.0233	0.5992	H	-3.1210	3.2675	0.3845	H	-3.3867	3.0287	0.5905
H	-4.0033	0.5962	1.3695	H	-3.9177	0.9555	1.3234	H	-4.0044	0.6033	1.3666
H	-5.1299	1.6453	-0.5705	H	-4.9499	1.9349	-0.7035	H	-5.1303	1.6497	-0.5752
H	-3.8692	1.0441	-1.6440	H	-3.7287	1.1655	-1.7130	H	-3.8700	1.0460	-1.6476
H	-5.7507	-0.6478	0.0724	H	-5.7415	-0.2525	0.0997	H	-5.7524	-0.6421	0.0716
H	-4.7059	-2.6315	-0.9442	H	-4.8357	-2.3774	-0.7522	H	-4.7086	-2.6279	-0.9419
H	-2.4334	-2.5038	0.0846	H	-2.5687	-2.3370	0.2942	H	-2.4365	-2.5000	0.0869
H	-3.6595	-1.8574	1.1657	H	-3.7523	-1.5234	1.3075	H	-3.6622	-1.8511	1.1667
H	-1.9369	-0.1842	-0.7143	H	-1.8974	-0.1261	-0.6695	H	-1.9380	-0.1819	-0.7154
H	-0.0459	0.9326	-0.9612	H	0.0735	0.8375	-0.9699	H	-0.0436	0.9353	-0.9619
H	1.4126	2.4055	-2.1429	H	1.6532	2.1129	-2.2318	H	1.4330	2.3893	-2.1471
H	2.1876	2.0723	0.8211	H	2.3646	1.9419	0.7618	H	2.1829	2.0795	0.8259
H	3.2982	-1.8640	-1.6899	H	3.2697	-2.0852	-1.4788	H	3.2913	-1.8639	-1.6881
H	-3.6242	-1.6179	-1.8916	H	-3.6760	-1.5186	-1.7582	H	-3.6264	-1.6165	-1.8909
H	1.6688	-3.9019	-0.1082	H	3.1133	-4.3718	-0.4041	H	1.6515	-3.9003	-0.0969
H	1.7427	-3.8067	-1.8869	H	1.4195	-4.0270	-0.0016	H	1.7330	-3.8268	-1.8715
H	3.3608	-5.2459	-0.8563	H	1.1650	-3.6913	-2.3332	H	3.9564	-4.3168	-1.6745
H	-2.4689	0.2700	3.0664	H	-2.4234	0.6445	3.0627	H	-2.1524	-1.4597	2.8825
H	-2.1497	-1.4664	2.8823	H	-2.2313	-1.1194	3.0082	H	-0.8814	-0.3825	3.4687
H	-0.8786	-0.3896	3.4692	H	-0.8885	-0.0978	3.5315	H	-2.4714	0.2771	3.0646
H	3.3268	4.2944	0.6650	H	5.0681	4.1604	-0.6011	H	4.3899	2.9473	0.3087
H	4.7193	4.5508	-0.4183	H	4.6369	2.6287	0.2053	H	3.3212	4.3069	0.6565
H	4.3952	2.9431	0.2843	H	3.6463	4.0642	0.4708	H	4.7284	4.5440	-0.4123
H	-0.0698	3.9389	0.5471	H	0.2525	3.9379	0.3113	H	-1.6468	4.4742	1.1350
H	-1.6488	4.4688	1.1469	H	-1.2873	4.6210	0.8548	H	-0.4538	3.7704	2.2435
H	-0.4556	3.7627	2.2538	H	-0.1545	3.9130	2.0221	H	-0.0686	3.9410	0.5361
H	-6.6821	-1.4416	-2.1033	H	-6.7106	-1.1391	-2.0232	H	-6.8567	0.3162	-1.9525
H	-5.5861	-0.3645	-2.9807	H	-5.5324	-0.2123	-2.9635	H	-6.6844	-1.4388	-2.1028
H	-6.8554	0.3131	-1.9501	H	-6.7575	0.6301	-2.0033	H	-5.5878	-0.3639	-2.9820
H	5.1581	-1.7784	0.1804	H	4.2545	-3.2514	1.5081	H	5.1585	-1.7705	0.1973
H	4.5625	-1.4003	1.8203	H	5.0882	-2.1013	0.4325	H	4.5482	-1.4282	1.8379
H	4.4229	-3.0575	1.1670	H	4.5008	-1.5701	2.0313	H	4.4144	-3.0721	1.1508
N	3.0742	-1.6302	0.3996	N	3.0171	-1.7953	0.6050	N	3.0717	-1.6302	0.4010
O	-0.1638	-2.4204	1.2364	O	-0.3088	-2.3336	1.4536	O	-0.1671	-2.4165	1.2373
O	3.3159	4.0480	-2.4294	O	3.6731	3.5904	-2.6009	O	3.3486	4.0174	-2.4341
O	0.6482	-1.2837	-2.1095	O	0.5742	-1.5792	-1.9040	O	0.6514	-1.2778	-2.1110
O	2.5115	-0.1508	2.0814	O	2.4902	-0.2220	2.2116	O	2.5115	-0.1500	2.0832
O	3.5475	-4.3007	-0.9506	O	1.9184	-4.2242	-2.0311	O	3.4592	-4.4204	-0.8494

Table S13. Cartesian coordinates of the six lowest-energy conformers of the 3'*S*,5'*S* stereoisomer of pyrenosetins F at the B3LYP/TZVP/SMD($CHCl_3$)//B3LYP/6-31+G(d,p)/SMD($CHCl_3$) level.

Conformer 1				Conformer 2				Conformer 3			
C	0.4335	-1.4802	-0.9098	C	-0.3659	-1.2852	1.0911	C	0.3274	-1.4586	-0.8155
C	1.3995	-0.3732	-1.3200	C	-1.3603	-0.1659	1.3733	C	1.3290	-0.4186	-1.3052
C	0.5201	0.9097	-1.2666	C	-0.5190	1.1233	1.1566	C	0.5190	0.9092	-1.2626
C	1.3076	2.1988	-1.1336	C	-1.3469	2.3644	0.8814	C	1.3762	2.1593	-1.2134
C	2.5970	2.1918	-0.7654	C	-2.6401	2.2765	0.5384	C	2.6792	2.0973	-0.9032
C	3.4036	0.9607	-0.4523	C	-3.4112	0.9937	0.3829	C	3.4347	0.8372	-0.5783
C	4.3479	1.1562	0.7524	C	-4.3755	1.0208	-0.8216	C	4.4431	1.0279	0.5739
C	5.2404	-0.0750	0.9914	C	-5.2273	-0.2585	-0.9062	C	5.2794	-0.2398	0.8248
C	4.3819	-1.3470	1.1139	C	-4.3260	-1.5065	-0.9014	C	4.3612	-1.4569	1.0387
C	3.4125	-1.5279	-0.0660	C	-3.3376	-1.5228	0.2767	C	3.3306	-1.6318	-0.0890
C	2.5220	-0.2832	-0.2242	C	-2.4908	-0.2387	0.2837	C	2.5010	-0.3477	-0.2606
C	1.9516	-0.6553	-2.7259	C	-1.8967	-0.2923	2.8072	C	1.8050	-0.7803	-2.7205
C	-0.5158	0.6429	-0.1169	C	0.5207	0.7618	0.0378	C	-0.4746	0.7431	-0.0598
C	-1.7288	1.5235	-0.1093	C	1.7264	1.6573	-0.0190	C	-1.6459	1.6796	-0.0461
C	-2.1608	2.2032	0.9645	C	2.2337	2.1613	-1.1560	C	-2.0517	2.3664	1.0332
C	-3.3840	3.0499	0.9148	C	3.4244	3.0398	-1.2419	C	-3.2410	3.2613	0.9878
C	-3.7892	3.7146	2.2124	C	4.1494	3.4495	0.0256	C	-3.5940	3.9704	2.2762
C	0.5970	3.4775	-1.5011	C	-0.6680	3.6986	1.0660	C	0.7126	3.4604	-1.5893
C	6.1507	0.1135	2.2092	C	-6.1595	-0.2377	-2.1219	C	6.2536	-0.0528	1.9924
C	-2.0127	-1.2529	-1.1703	C	2.0981	-1.0882	1.0947	C	-2.1227	-1.1306	-0.9044
C	-0.8460	-0.8667	-0.2616	C	0.8556	-0.7319	0.2876	C	-0.8794	-0.7538	-0.1121
C	-1.1680	-1.5847	1.0503	C	1.0398	-1.5760	-0.9850	C	-1.1345	-1.4061	1.2526
C	-2.8360	-2.3421	-0.4762	C	2.6574	-2.4040	0.5488	C	-2.8778	-2.2178	-0.1480
C	-4.3305	-1.9899	-0.4667	C	4.1917	-2.4317	0.5094	C	-4.3903	-1.9028	-0.1110
C	-2.6872	-3.3557	1.8594	C	2.3646	-3.5192	-1.7283	C	-2.6236	-3.1188	2.2255
H	-0.0565	0.9564	-2.2006	H	0.0594	1.2974	2.0758	H	-0.0964	0.9526	-2.1723
H	3.1346	3.1398	-0.7237	H	-3.2054	3.1970	0.3885	H	3.2655	3.0168	-0.9193
H	4.0523	0.7618	-1.3225	H	-4.0429	0.8759	1.2798	H	4.0303	0.5709	-1.4682
H	4.9734	2.0433	0.5869	H	-5.0293	1.8998	-0.7472	H	5.1061	1.8723	0.3436
H	3.7494	1.3584	1.6527	H	-3.7948	1.1393	-1.7479	H	3.8986	1.2980	1.4906
H	5.8807	-0.1919	0.1031	H	-5.8514	-0.2966	0.0005	H	5.8709	-0.4269	-0.0851
H	5.0324	-2.2267	1.1975	H	-4.9459	-2.4117	-0.8779	H	4.9674	-2.3669	1.1313
H	2.8030	-2.4231	0.0902	H	-2.6984	-2.4087	0.2100	H	2.6826	-2.4854	0.1323
H	3.9870	-1.6949	-0.9864	H	-3.8954	-1.6075	1.2184	H	3.8524	-1.8673	-1.0256
H	2.0122	-0.1385	0.7388	H	-1.9916	-0.1850	-0.6940	H	2.0416	-0.1383	0.7158
H	-0.0067	0.7428	0.8473	H	0.0217	0.7899	-0.9361	H	0.0783	0.8632	0.8775
H	-1.6230	2.1509	1.9092	H	1.7703	1.9409	-2.1160	H	-1.5166	2.2872	1.9774
H	-2.3063	1.6007	-1.0287	H	2.2045	1.8796	0.9318	H	-2.2168	1.7899	-0.9661
H	-2.6960	-3.2916	-1.0149	H	2.3199	-3.2155	1.2136	H	-2.7398	-3.1796	-0.6641
H	3.8042	-1.2984	2.0486	H	-3.7608	-1.5416	-1.8442	H	3.8304	-1.3392	1.9948
H	-4.6596	-1.8931	-1.5049	H	4.5514	-2.3022	1.5384	H	-4.8978	-2.6279	0.5316
H	-4.9007	-2.8100	-0.0082	H	4.5239	-3.4156	0.1657	H	-4.5417	-0.9028	0.3234
H	-4.3513	-0.8325	1.1001	H	4.6940	-0.6082	0.0357	H	-4.4885	-1.3947	-1.9714
H	2.6303	0.1399	-3.0456	H	-1.0748	-0.2371	3.5286	H	2.5123	-0.0367	-3.0970
H	2.4887	-1.6061	-2.7580	H	-2.5956	0.5164	3.0362	H	2.2874	-1.7603	-2.7380
H	1.1345	-0.7092	-3.4526	H	-2.4065	-1.2467	2.9574	H	0.9565	-0.8140	-3.4118
H	-4.7017	4.2959	2.0705	H	4.4878	2.5746	0.5909	H	-4.4830	4.5876	2.1372
H	-2.9851	4.3714	2.5664	H	3.4872	4.0246	0.6823	H	-2.7560	4.5985	2.6026
H	-3.9484	2.9609	2.9931	H	5.0115	4.0629	-0.2410	H	-3.7711	3.2400	3.0750
H	-0.2778	3.6592	-0.8669	H	-0.2525	3.7934	2.0782	H	0.2842	3.4056	-2.5989
H	1.2633	4.3408	-1.4131	H	0.1685	3.8316	0.3703	H	-0.1148	3.7097	-0.9153
H	0.2271	3.4371	-2.5342	H	-1.3683	4.5243	0.9088	H	1.4256	4.2899	-1.5680
H	5.5621	0.2352	3.1271	H	-5.5874	-0.1991	-3.0571	H	6.9286	0.7934	1.8189
H	6.7847	1.0012	2.1017	H	-6.8231	0.6346	-2.1027	H	6.8713	-0.9457	2.1425
H	6.8098	-0.7508	2.3512	H	-6.7893	-1.1343	-2.1559	H	5.7153	0.1387	2.9289
H	-2.6650	-4.3730	1.4534	H	1.6598	-3.4548	-2.5575	H	-1.8712	-3.0617	3.0125
H	-2.0091	-3.2913	2.7106	H	3.3784	-3.3595	-2.1087	H	-3.5955	-2.8386	2.6438
H	-3.7049	-3.1356	2.1990	H	2.3020	-4.5151	-1.2767	H	-2.6810	-4.1465	1.8513
N	-2.2415	-2.4031	0.8562	N	2.0096	-2.4998	-0.7569	N	-2.2237	-2.2185	1.1557
O	0.5811	-2.6729	-1.0715	O	-0.4574	-2.4435	1.4393	O	0.3974	-2.6609	-0.9509
O	-4.0272	3.2066	-0.1170	O	3.8059	3.4321	-2.3406	O	-3.8994	3.4168	-0.0351
O	-2.2542	-0.8027	-2.2685	O	2.5889	-0.4529	2.0045	O	-2.4917	-0.6406	-1.9558
O	-0.5379	-1.4544	2.0935	O	0.3870	-1.4399	-2.0135	O	-0.4433	-1.2329	2.2493
O	-4.5848	-0.7465	0.1642	O	4.7484	-1.4803	-0.3807	O	-4.9592	-2.0233	-1.3995

Table S13 (continued)

Conformer 4				Conformer 5				Conformer 6			
C	0.3163	-1.3876	-0.9344	C	0.3230	-1.3718	-0.9648	C	0.3149	-1.3655	-0.9138
C	1.3797	-0.3683	-1.3299	C	1.3849	-0.3441	-1.3401	C	1.3129	-0.2922	-1.3315
C	0.6233	0.9891	-1.2504	C	0.6237	1.0093	-1.2435	C	0.5073	1.0295	-1.1770
C	1.5275	2.1973	-1.1001	C	1.5236	2.2181	-1.0724	C	1.3698	2.2695	-1.0372
C	2.8130	2.0642	-0.7427	C	2.8079	2.0836	-0.7114	C	2.6764	2.1795	-0.7503
C	3.5028	0.7580	-0.4559	C	3.5004	0.7755	-0.4399	C	3.4307	0.8958	-0.5335
C	4.4705	0.8464	0.7427	C	4.4617	0.8497	0.7648	C	4.4543	0.9940	0.6168
C	5.2441	-0.4669	0.9570	C	5.2376	-0.4645	0.9648	C	5.2876	-0.2925	0.7578
C	4.2694	-1.6528	1.0725	C	4.2657	-1.6546	1.0587	C	4.3656	-1.5179	0.8928
C	3.2779	-1.7261	-0.1010	C	3.2806	-1.7141	-0.1209	C	3.3191	-1.6030	-0.2309
C	2.5093	-0.4005	-0.2379	C	2.5092	-0.3887	-0.2430	C	2.4947	-0.3059	-0.2957
C	1.8984	-0.6775	-2.7427	C	1.9116	-0.6302	-2.7547	C	1.7748	-0.5422	-2.7753
C	-0.4284	0.8040	-0.1005	C	-0.4318	0.8057	-0.0999	C	-0.4813	0.7765	0.0148
C	-1.5641	1.7832	-0.0895	C	-1.5736	1.7779	-0.0884	C	-1.6644	1.7003	0.0809
C	-1.9858	2.4403	1.0024	C	-2.0113	2.4194	1.0063	C	-2.0892	2.2930	1.2087
C	-3.1411	3.3776	0.9528	C	-3.1699	3.3528	0.9537	C	-3.2708	3.1824	1.3113
C	-3.5084	4.0555	2.2540	C	-3.5645	4.0031	2.2609	C	-4.0899	3.4884	0.0731
C	0.9335	3.5435	-1.4311	C	0.9268	3.5671	-1.3862	C	0.7068	3.5998	-1.2931
C	6.1790	-0.3813	2.1677	C	6.1658	-0.3933	2.1817	C	6.2800	-0.1987	1.9210
C	-2.1090	-0.9503	-1.1177	C	-2.1064	-0.9531	-1.1249	C	-2.1382	-1.0669	-0.8911
C	-0.8886	-0.6710	-0.2476	C	-0.8815	-0.6713	-0.2612	C	-0.8722	-0.7188	-0.1241
C	-1.2321	-1.3749	1.0690	C	-1.2074	-1.3934	1.0503	C	-1.0652	-1.4673	1.2029
C	-2.9555	-2.0285	-0.4363	C	-2.9466	-2.0336	-0.4419	C	-2.8233	-2.2412	-0.1999
C	-4.4390	-1.6596	-0.4620	C	-4.4381	-1.6515	-0.4300	C	-4.3460	-2.0040	-0.0988
C	-2.8335	-3.0521	1.9062	C	-2.7953	-3.0841	1.8867	C	-2.4729	-3.2856	2.1025
H	0.0508	1.1053	-2.1813	H	0.0543	1.1372	-2.1748	H	-0.1124	1.1463	-2.0778
H	3.4360	2.9576	-0.6868	H	3.4279	2.9780	-0.6400	H	3.2652	3.0960	-0.6996
H	4.1232	0.5121	-1.3346	H	4.1259	0.5439	-1.3189	H	4.0140	0.6959	-1.4485
H	5.1758	1.6727	0.5835	H	5.1657	1.6799	0.6206	H	5.1182	1.8511	0.4430
H	3.9007	1.0912	1.6510	H	3.8867	1.0805	1.6734	H	3.9231	1.1947	1.5585
H	5.8624	-0.6323	0.0607	H	5.8612	-0.6158	0.0698	H	5.8642	-0.4124	-0.1728
H	4.8338	-2.5914	1.1402	H	4.8324	-2.5925	1.1163	H	4.9675	-2.4352	0.9085
H	2.5868	-2.5608	0.0511	H	2.5907	-2.5525	0.0161	H	2.6692	-2.4678	-0.0647
H	3.8268	-1.9368	-1.0280	H	3.8347	-1.9105	-1.0479	H	3.8271	-1.7708	-1.1894
H	2.0183	-0.2216	0.7292	H	2.0131	-0.2248	0.7240	H	2.0455	-0.1686	0.6980
H	0.0864	0.8551	0.8644	H	0.0781	0.8529	0.8678	H	0.0697	0.8489	0.9578
H	-1.4886	2.3040	1.9607	H	-1.5255	2.2732	1.9689	H	-1.5617	2.1396	2.1485
H	-2.0961	1.9511	-1.0240	H	-2.0963	1.9545	-1.0268	H	-2.2037	1.8587	-0.8498
H	-2.8285	-2.9744	-0.9852	H	-2.8269	-2.9716	-1.0079	H	-2.6561	-3.1541	-0.7909
H	3.7063	-1.5609	2.0129	H	3.6974	-1.5773	1.9972	H	3.8481	-1.4694	1.8620
H	-4.6094	-0.7880	0.1866	H	-4.5851	-0.7873	0.2251	H	-4.8009	-2.8009	0.4960
H	-4.6884	-1.3723	-1.4925	H	-4.7144	-1.3564	-1.4504	H	-4.5313	-1.0505	0.4195
H	-6.1217	-2.5351	-0.0249	H	-5.2922	-3.3950	-0.5911	H	-4.5101	-1.3659	-1.9135
H	2.6481	0.0550	-3.0533	H	2.6600	0.1096	-3.0506	H	0.9195	-0.5242	-3.4586
H	2.3431	-1.6741	-2.7924	H	2.3602	-1.6243	-2.8172	H	2.4769	0.2303	-3.0996
H	1.0775	-0.6426	-3.4663	H	1.0941	-0.5874	-3.4817	H	2.2585	-1.5167	-2.8740
H	-3.7329	3.3060	3.0225	H	-3.8059	3.2367	3.0074	H	-3.4773	3.9857	-0.6869
H	-4.3718	4.7072	2.1109	H	-4.4249	4.6577	2.1135	H	-4.9203	4.1402	0.3486
H	-2.6601	4.6438	2.6249	H	-2.7243	4.5824	2.6628	H	-4.4835	2.5698	-0.3755
H	0.5452	3.5600	-2.4581	H	0.5420	3.5973	-2.4143	H	-0.0965	3.8017	-0.5752
H	0.0897	3.7922	-0.7773	H	0.0800	3.8039	-0.7320	H	1.4273	4.4205	-1.2287
H	1.6781	4.3397	-1.3371	H	1.6687	4.3640	-1.2782	H	0.2492	3.6268	-2.2911
H	6.7537	-1.3062	2.2933	H	5.5942	-0.2416	3.1058	H	6.9571	0.6551	1.8015
H	5.6129	-0.2151	3.0926	H	6.8790	0.4345	2.0933	H	6.8949	-1.1032	1.9936
H	6.8940	0.4431	2.0638	H	6.7424	-1.3183	2.2975	H	5.7568	-0.0760	2.8774
H	-2.8472	-4.0711	1.5055	H	-3.7902	-2.8139	2.2469	H	-3.4404	-3.0625	2.5631
H	-2.1484	-3.0022	2.7529	H	-2.8396	-4.0966	1.4710	H	-2.5093	-4.2891	1.6655
H	-3.8437	-2.7954	2.2321	H	-2.0859	-3.0627	2.7144	H	-1.7008	-3.2536	2.8716
N	-2.3567	-2.1212	0.8935	N	-2.3269	-2.1476	0.8755	N	-2.1308	-2.3070	1.0822
O	0.3449	-2.5836	-1.1310	O	0.3517	-2.5636	-1.1872	O	0.3738	-2.5516	-1.1553
O	-3.7631	3.5908	-0.0826	O	-3.7730	3.5840	-0.0890	O	-3.5712	3.6597	2.4016
O	-2.3813	-0.4231	-2.1749	O	-2.3865	-0.4261	-2.1805	O	-2.5725	-0.4996	-1.8775
O	-0.5713	-1.2853	2.0984	O	-0.5385	-1.3112	2.0750	O	-0.3486	-1.3385	2.1878
O	-5.1881	-2.7909	-0.0373	O	-5.2745	-2.6841	0.0671	O	-4.9466	-2.0491	-1.3783
